# Supplementary material for: An extended Tudor domain within Vreteno interconnects Gtsf1L and Ago3 for piRNA biogenesis in Bombyx mori
Source: EMBO J. 2023 Nov 20;42(24):e114072. doi: 10.15252/embj.2023114072 (PMC10711660; doi:10.15252/embj.2023114072)
Supplement: Supplementary file 1 — Expanded View Figures PDF [file EMBJ-42-e114072-s004.pdf]

Expanded View Figures

Figure EV1. BmGtsf1L and BmVreteno both interact with BmAgo3.

A Domain organization (top) and ClustalW alignment of GTSF proteins from different species. The alignment and conservation scores are depicted using the Jalview software. Residues that are highlighted in blue reveal a 20% identity threshold
B Western blot detection using the mouse monoclonal anti-BmGtsf1L antibody on naïve BmN4 cell extracts or on BmN4 cells that were transfected with FLAG-BmGtsf1L.
C Validation of stable integration of FLAG-Siwi, FLAG-BmAgo3, or FLAG-eGFP expression cassettes into BmN4 cells after extensive puromycin selection by Western blot using the indicated antibodies. Anti-actin probing served as a loading control.
D Control or GFP (BmGtsf1L) immunoprecipitation on BmN4 cell extracts from FLAG-PIWI stable cells that were co-transfected with BmGtsf1L-eGFP. Western blot was performed using the indicated antibodies, and anti-actin probing as well as Ponceau S staining served as loading controls.
E Nucleotide composition of small RNAs that were sequenced from input samples or from anti-HA immunoprecipitated samples.
F Pfam/SMART-based domain organization of the BmVreteno-Long and BmVreteno-Short isoforms, showing the RNA-recognition motif (RRM), Myeloid translocation protein 8, Nervy and DEAF-1 (MYND) domain, and two C-terminal Tudor domains (TD).
G Western blot using the rabbit polyclonal anti-BmVreteno antibody on cell extracts from BmN4 cells that were either untransfected or transfected with HA-BmVreteno (L)-FL. Anti-tubulin probing served as a loading control.
H Validation of anti-BmVreteno antibody specificity on cell extracts from BmN4 cells that were transfected four consecutive times with dsRNA against Luciferase (Luc) or against BmVreteno.
I IgG or anti-BmVreteno immunoprecipitation on naïve BmN4 cells, followed by Western blot detection of endogenous BmVreteno and BmAgo3.
J Reciprocal IP on BmN4 cell extracts using non-immune (n.i.) serum or anti-BmAgo3 antibodies as well as endogenous BmVreteno antibodies for Western blot detection of retrieved proteins.

© 2023 The Authors

© 2023 The Authors

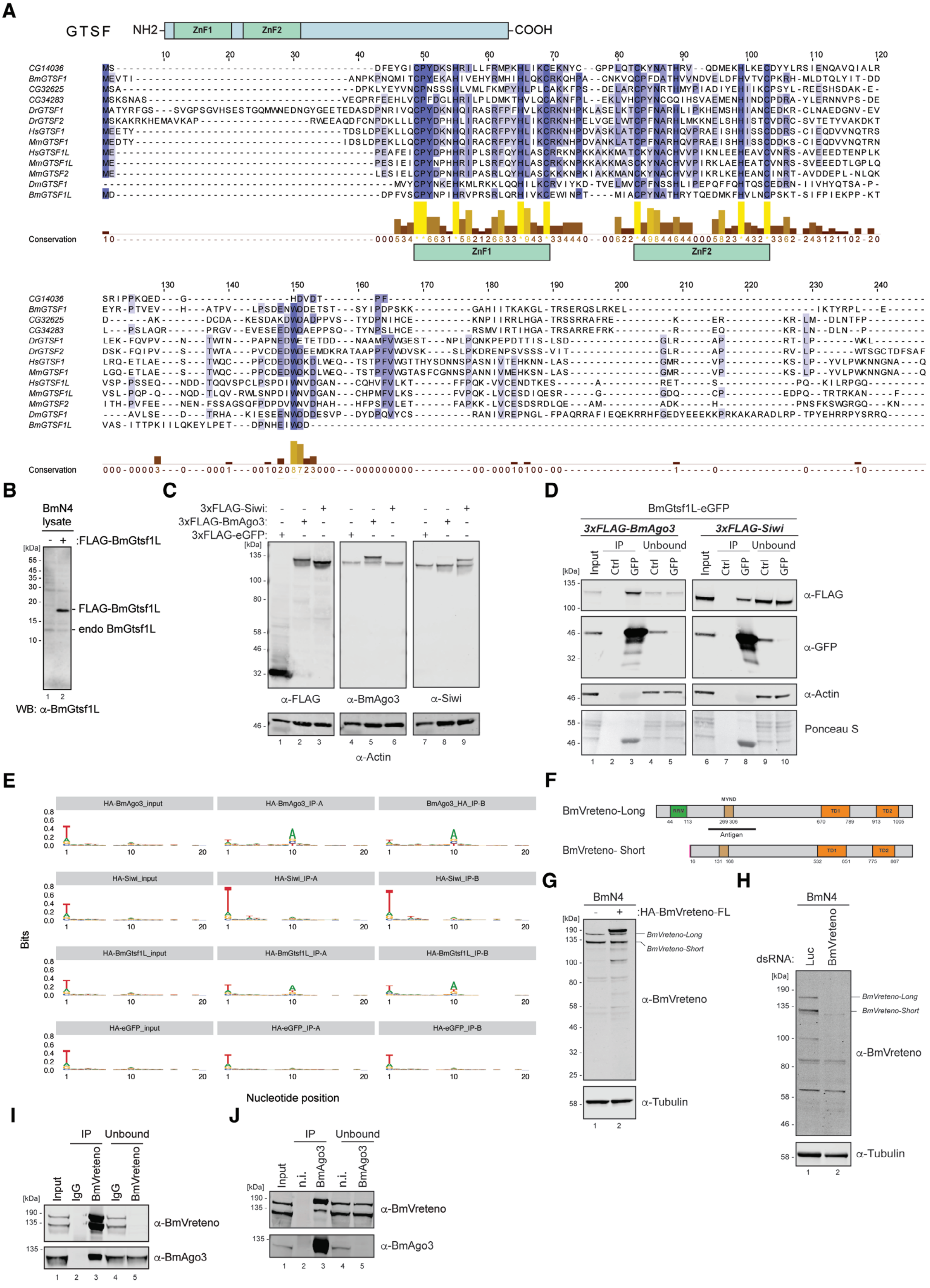

Figure EV1.

 © 2023 The Authors

Figure EV2. BmAgo3 interacts with BmVreteno eTD1 via methylated arginine residues.

A Fluorescence lifetime imaging of BmN4 cells (using all channels) transfected with either eGFP-BmVreteno, BmGtsf1L- mOrange2, or mCardinal-BmAgo3. Inset shows the zoom-in of the boxed area. Two representative images of two biological experiments are shown. Scale bars: 10 μm. Plots on the right show the normalized intensity (gray values) for each channel of the line that has been drawn in the inset frame. Quantification data can be found in Dataset EV4.

B Multiple sequence alignments of Tudor domains expressed in BmVreteno and its orthologue in *Drosophila* (DmVret). In addition, eTudor11 from the *Drosophila* Tudor protein as well as the eTudor domain of *Drosophila* Tudor-SN (p100), for which crystal structures have been resolved (PDB: 3NTH and 2WAC, respectively), were included. Alignments were performed using Clustal Omega and processed using Jalview software. Aromatic cage residues are depicted as green boxes, and the asparagine residue that is involved in directly binding to the methylated arginine residue (sDMA) is highlighted in yellow. Identical residues (*), conserved substitutions (:) or substitutions by weakly similar residues (.) are indicated below the alignment.

C Anti-FLAG immunoprecipitation of FLAG-BmAgo3 variants that were transiently expressed in the HA-BmGtsf1L-eGFP stable BmN4 cell line. The transfection of 3xFLAG-mCherry served as a control. Immunoprecipitated proteins were analyzed by Western blot using the indicated antibodies, whereas Ponceau S staining served as a loading control.

© 2023 The Authors

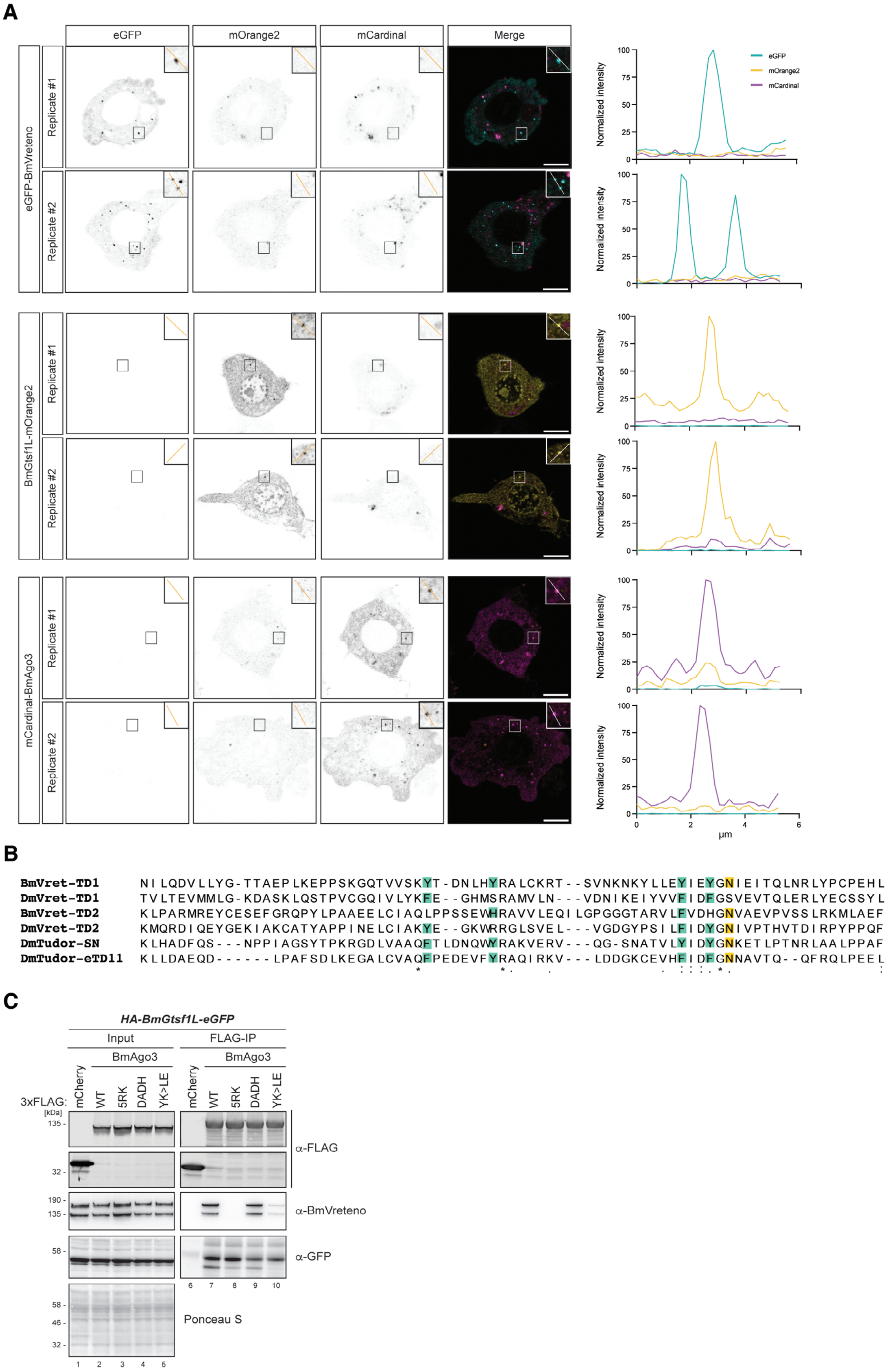

Figure EV2.

 © 2023 The Authors

Figure EV3. The BmGtsf1L C-terminus establishes an interaction with BmVreteno.

A Outlined strategy for the purification of recombinant GST-tagged BmVreteno-S, showing the profiles from the size-exclusion column (left) and the peak fractions that were analyzed by SDS-PAGE followed by Coomassie staining (right).

B Similar to panel (A), but for GST-BmVreteno-L.

C Size-exclusion profiles of BmVreteno-S (left) and BmVreteno-L (right) to compare the shift in molecular weight before (green line) and after (blue line) 3C-mediated cleavage of the GST tag.

D Pfam/SMART-based domain organization of the BmVreteno-Long isoform, showing the RNA-recognition motif (RRM), Myeloid translocation protein 8, Nervy and DEAF-1 (MYND) domain, and two C-terminal Tudor domains (TD). In addition to the full-length (FL) BmVreteno, the C-and N-terminal truncation variants are depicted and are used in panel (E) to analyze the interaction between BmGtsf1L and BmVreteno variants.

E Co-transfection of BmGtsf1L-eGFP with HA-BmVreteno truncation variants in which different domains were omitted. The transfection of HA-LacZ served as a control. BmGtsf1L was retrieved by GFP-IP, and input and elution fractions were analyzed by SDS-PAGE, followed by Western blot using the indicated antibodies. Anti-actin immunodetection served as a loading control.

© 2023 The Authors

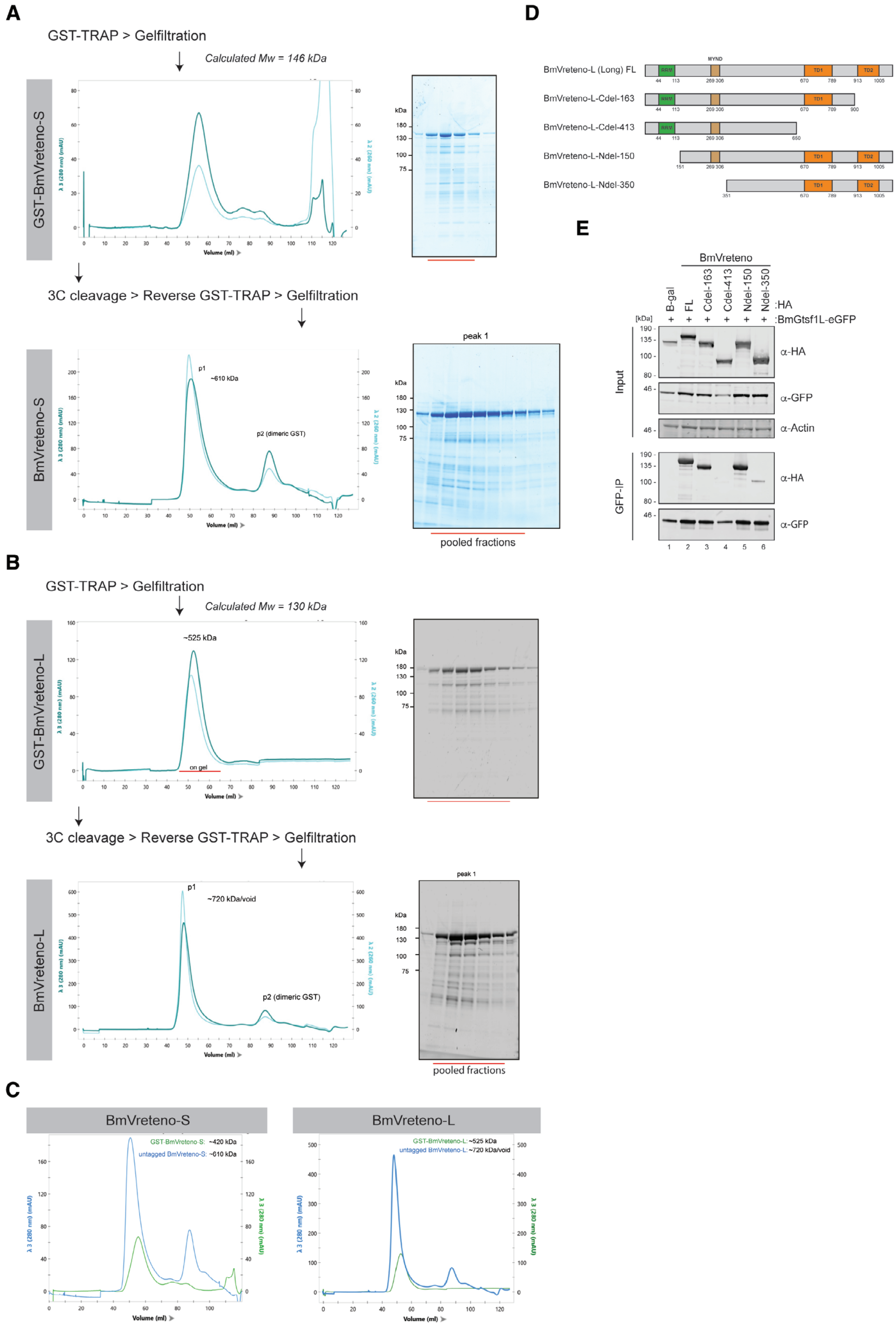

Figure EV3.

 © 2023 The Authors

Figure EV4. The hydrophobic binding pocket is unique to BmVreteno AF-eTD1 and facilitates BmGtsf1L binding.

A Superimposition of the AlphaFold structural models of all three BmVreteno AF-eTD domains. The central inset (closed circle) shows the side view of the aromatic cage, which is only present in AF eTD1 and indicated with a dashed circle. The top inset (closed circle) shows a top view of the novel interface, which is unique to AF eTD1. The hydrophobic pocket is indicated with a dashed circle, and the enlarged view additionally shows the docked C-terminal motif of BmGtsf1L.

B Snapshot on the novel hydrophobic binding pocket of BmVreteno AF-eTD1 (blue) and contacts between the residues R742, S744, K749, and I762 (shown as sticks) with BmGtsf1L C-terminal 10-AA residues (shown as yellow sticks). This snapshot displays the BmVreteno S744 residue that forms a hydrogen bond with the backbone carbonyl of BmGtsf1L I98.

C Plots showing the distances between the atoms forming the four most important inter-chain hydrogen bonds of the side chain of BmVreteno S744 in two out of the ten 1 μs simulation runs. The run presented in the upper panel displays the hydrogen bond between the side chain of S744 and the backbone carbonyl of BmGtsf1L I98. The simulation presented in the bottom panel reveals that S744 is engaged in different interactions with BmGtsf1L residues I98 and D100. Overall, the simulations reveal that some of the hydrogen bonds are transiently formed and broken.

D Single-plane confocal micrographs of BmN4 cells co-transfected with different eGFP-BmVreteno constructs (upper panel) and BmGtsf1L-mCherry (middle panel). Yellow triangles indicate a formed granule. Scale bars: 4 μm.

E Transfection of BmN4 cells with BmGtsf1L-eGFP together with HA-BmVreteno-FL. The transfection of HA-LacZ served as a control. A GFP (BmGtsf1L) immunoprecipitation was performed on BmN4 lysates, and input as well as elution samples were resolved by SDS-PAGE. Proteins were detected by Western blot using the indicated antibodies, and anti-actin probing as well as Ponceau S staining served as a loading control.

© 2023 The Authors

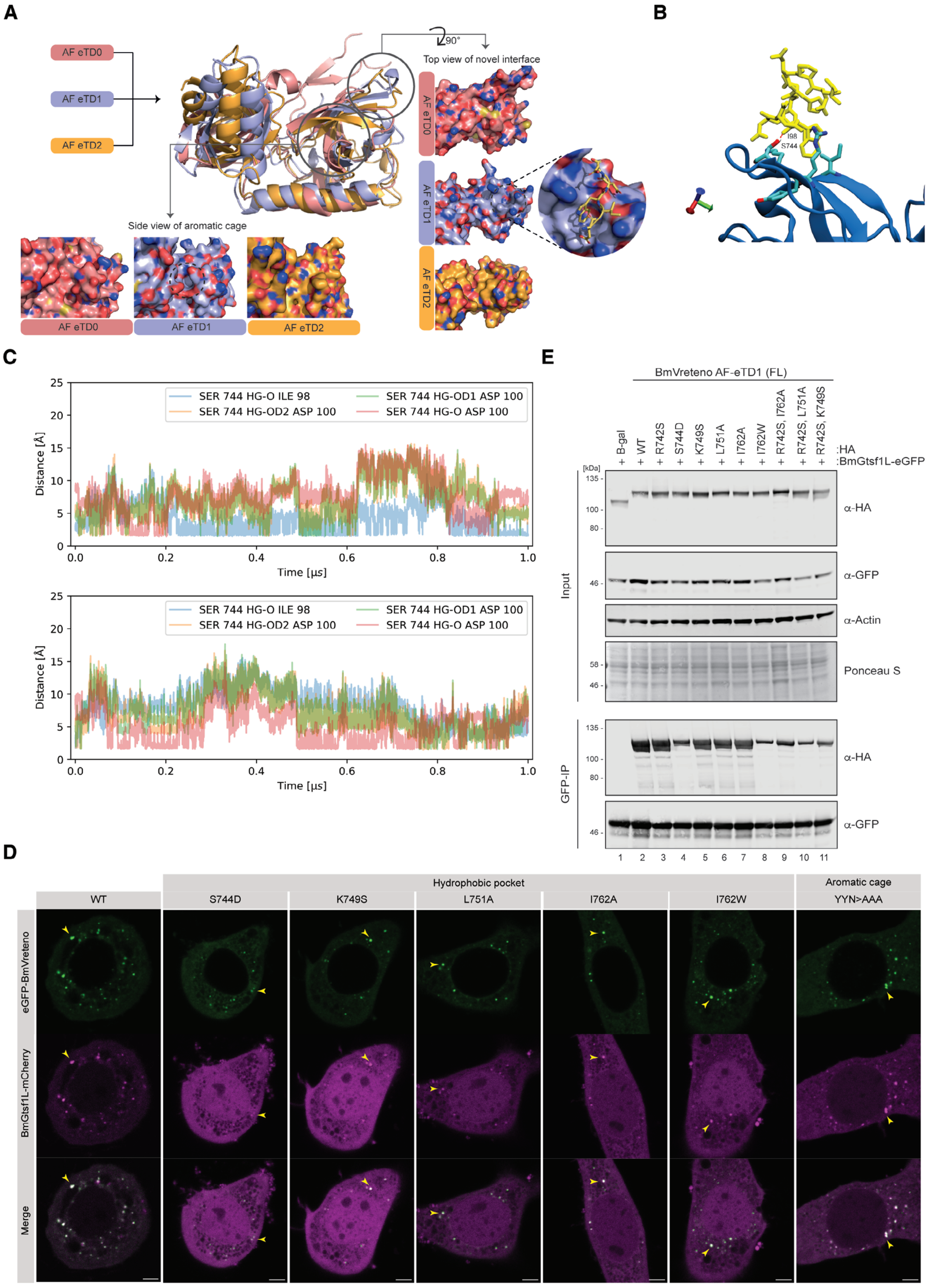

Figure EV4.

 © 2023 The Authors
