# Supplementary material for: An extended Tudor domain within Vreteno interconnects Gtsf1L and Ago3 for piRNA biogenesis in Bombyx mori
Source: EMBO J. 2023 Nov 20;42(24):e114072. doi: 10.15252/embj.2023114072 (PMC10711660; doi:10.15252/embj.2023114072)
Supplement: Supplementary file 8 — PDF+ [file EMBJ-42-e114072-s003.pdf]

Article

SOURCE DATA | TRANSPARENT PROCESS | OPEN ACCESS

THE EMBO JOURNAL

An extended Tudor domain within Vreteno interconnects Gtsf1L and Ago3 for piRNA biogenesis in *Bombyx mori*

Alfred W Bronkhorst[1,*], Chop Y Lee[2,3], Martin M Möckel[4], Sabine Ruegenberg[4], Antonio M de Jesus Domingues[1,†], Shéraz Sadouki[1], Rossana Piccinno[5], Tetsutaro Sumiyoshi[6,‡], Mikiko C Siomi[6], Lukas Stelzl[7,8], Katja Luck[3,**] & René F Ketting[1,9,***]

Abstract

Piwi-interacting RNAs (piRNAs) direct PIWI proteins to transposons to silence them, thereby preserving genome integrity and fertility. The piRNA population can be expanded in the ping-pong amplification loop. Within this process, piRNA-associated PIWI proteins (piRISC) enter a membraneless organelle called nuage to cleave their target RNA, which is stimulated by Gtsf proteins. The resulting cleavage product gets loaded into an empty PIWI protein to form a new piRISC complex. However, for piRNA amplification to occur, the new RNA substrates, Gtsf-piRISC, and empty PIWI proteins have to be in physical proximity. In this study, we show that in silkworm cells, the Gtsf1 homolog BmGtsf1L binds to piRNA-loaded BmAgo3 and localizes to granules positive for BmAgo3 and BmVreteno. Biochemical assays further revealed that conserved residues within the unstructured tail of BmGtsf1L directly interact with BmVreteno. Using a combination of AlphaFold modeling, atomistic molecular dynamics simulations, and *in vitro* assays, we identified a novel binding interface on the BmVreteno-eTudor domain, which is required for BmGtsf1L binding. Our study reveals that a single eTudor domain within BmVreteno provides two binding interfaces and thereby interconnects piRNA-loaded BmAgo3 and BmGtsf1L.

Keywords Ago3; eTudor; Gtsf; piRNA; Vreteno
Subject Categories RNA Biology; Structural Biology
DOI 10.15252/embj.2023114072 | Received 22 March 2023 | Revised 25 October 2023 | Accepted 30 October 2023 | Published online 20 November 2023
The EMBO Journal (2023) 42: e114072

Introduction

Animal germ cells utilize the Piwi-interacting (pi)RNA pathway as a mechanism to silence transposons, thereby maintaining genome stability and fertility (Czech *et al*, 2018; Ozata *et al*, 2019). A defective piRNA pathway leads to transposon derepression, DNA damage, gametogenesis defects, and sterility. The piRNA pathway can also have non-transposon targets, such as in the silk moth *Bombyx mori*, where piRNAs regulate sex determination (Kiuchi *et al*, 2014). piRNAs are about 24–31 nucleotides in size and associate with PIWI-clade Argonaute proteins to guide them to complementary targets (Ghildiyal & Zamore, 2009). Therefore, piRNAs are a key, specificity-determining component of the Piwi pathway.

In *Drosophila* germ cells, precursor piRNAs (pre-pre-piRNAs) are transcribed within large dual-strand clusters and exported from the nucleus for subsequent processing within the cytoplasm (Brennecke *et al*, 2007; Czech *et al*, 2018; Ozata *et al*, 2019). piRNAs can be loaded into different PIWI proteins. *Drosophila* expresses three PIWI proteins: Piwi, Aubergine (Aub), and Ago3. Piwi and Aub are predominantly loaded with antisense piRNAs, whereas Ago3 mostly incorporates sense piRNAs (Brennecke *et al*, 2007; Gunawardane *et al*, 2007). In the silkworm, only two cytoplasmic PIWI proteins are expressed: Siwi and BmAgo3 (Kawaoka *et al*, 2009). Siwi-associated piRNAs are mostly antisense and are responsible for the cleavage of sense transposon mRNA, whereas BmAgo3 binds sense piRNAs and triggers antisense piRNA precursor cleavage.

The current model for piRNA biogenesis suggests that cytoplasmic piRNA processing occurs through two interconnected mechanisms.

1 Biology of Non-coding RNA Group, Institute of Molecular Biology, Mainz, Germany
2 International PhD Programme on Gene Regulation, Epigenetics & Genome Stability, Mainz, Germany
3 Integrative Systems Biology Group, Institute of Molecular Biology, Mainz, Germany
4 Protein Production Core Facility, Institute of Molecular Biology, Mainz, Germany
5 Microscopy Core Facility, Institute of Molecular Biology, Mainz, Germany
6 Department of Biological Sciences, Graduate School of Science, The University of Tokyo, Tokyo, Japan
7 Faculty of Biology, Johannes Gutenberg University Mainz, Mainz, Germany
8 KOMET 1, Institute of Physics, Johannes Gutenberg University Mainz, Mainz, Germany
9 Institute of Developmental Biology and Neurobiology, Johannes Gutenberg University, Mainz, Germany
*Corresponding author. Tel: +49 6131 39 21474; E-mail: w.bronkhorst@imb-mainz.de
**Corresponding author. Tel: +49 6131 39 21440; E-mail: k.luck@imb-mainz.de
***Corresponding author. Tel: +49 6131 39 21470; E-mail: r.ketting@imb-mainz.de
†Present address: Dewpoint Therapeutics GmbH, Dresden, Germany
‡Present address: Department of Medical Innovations, Osaka Research Center for Drug Discovery, Otsuka Pharmaceutical Co., Ltd., Osaka, Japan

© 2023 The Authors. Published under the terms of the CC BY 4.0 license

One step of piRNA processing takes place within the nuage, a germline-specific phase-separated structure that surrounds the nuclear membrane. Here, piRNA-guided (called trigger piRNA) endonuclease activity of one PIWI protein (e.g., Ago3) generates the 5′ monophosphate end of a complementary piRNA precursor transcript (Han *et al*, 2015; Homolka *et al*, 2015; Mohn *et al*, 2015; Gainetdinov *et al*, 2018). This so-called responder pre-piRNA is subsequently incorporated into an unloaded PIWI protein (*Drosophila* Aub or silkworm Siwi), which is often still too long at its 3′-end. For further pre-piRNA processing, the PIWI protein then migrates to the mitochondrial outer membrane. Here, the second step of piRNA processing is mediated by the endonuclease Zucchini (Zuc), which mediates responder pre-piRNA 3′-end formation by cleaving 5′ to an available uridine (Han *et al*, 2015; Mohn *et al*, 2015). Pre-piRNA 3′-end resection is further completed by trimming and methylation to generate a mature piRISC complex (Horwich *et al*, 2007; Kawaoka *et al*, 2011; Hayashi *et al*, 2016; Izumi *et al*, 2016). The mature piRISC complex is liberated from the mitochondria into the cytosol to cleave complementary RNA, resulting in target RNA degradation. Alternatively, the mature piRISC complex (Aub or Siwi) can transit back to the nuage (now serving as a trigger piRNA) to bind complementary target RNA. This initiates a new round of PIWI-catalyzed responder piRNA biogenesis, leading to new piRISC formation. This consecutive and continuous process of responder and trigger piRNA production, which requires reciprocal cleavages by two paired PIWI proteins, is called the piRNA amplification cycle (or ping-pong loop). Thus, mature piRNAs within the ping-pong cycle are generated by the combined action of PIWI-slicing and Zuc-cleavage.

Notably, Zuc-mediated processing of the responder pre-piRNA 3′-end simultaneously generates the 5′-end of a new pre-piRNA substrate for phased piRNA biogenesis, which results in the production of trailer piRNAs (Han *et al*, 2015; Mohn *et al*, 2015). Initiator and responder piRNAs that are generated via the ping-pong cycle increase the abundance of an existing pool of piRNAs, whereas the Zuc-dependent trailer piRNAs expand the repertoire of piRNA sequences. In *Drosophila*, phased piRNAs predominantly associate with Piwi and translocate to the nucleus to induce transcriptional gene silencing through the deposition of repressive chromatin marks (Czech *et al*, 2018). Even though trailer piRNAs are produced, the silkworm does not possess a nuclear piRNA-based silencing pathway (Gainetdinov *et al*, 2018; Izumi *et al*, 2020).

Efficient piRNA amplification within the ping-pong cycle requires that PIWI-mediated target cleavage is confined to molecular surroundings that are compatible with an empty PIWI protein receiving one of the cleavage products. It has been suggested that Tudor-domain-containing proteins that reside in the nuage can provide such an environment by acting as a molecular scaffold (Chen *et al*, 2011; Siomi *et al*, 2011). Tudor domains that harbor an aromatic cage can bind to symmetrically dimethylated arginine residues (sDMAs) on client proteins but can also establish sDMA-independent protein interactions (Siomi *et al*, 2010; Chen *et al*, 2011). For example, the *Drosophila* Krimper protein makes sure that cleavage products resulting from Aub-slicing are efficiently loaded into empty Ago3 (Sato *et al*, 2015; Webster *et al*, 2015). Krimper binds sDMA-methylated piRISC-Aub via its aromatic cage-containing Tudor domain, whereas an upstream Tudor domain within Krimper establishes the sDMA-independent interaction with empty Ago3 (Sato *et al*, 2015; Webster *et al*, 2015; Huang *et al*, 2021). Likewise, the multi-Tudor domain-containing protein Qin also promotes heterotypic ping-pong between piRISC-Aub and empty, unmethylated Ago3 (Zhang *et al*, 2011, 2014). In silkworms, the handover of piRISC-Siwi-cleaved target RNA to empty BmAgo3 is mediated by the RNA-helicase Vasa (Xiol *et al*, 2014; Nishida *et al*, 2015). Notably, Vasa contains intrinsically disordered regions that are involved in the formation of phase-separated structures and seems to be the scaffold for nuage formation (Nott *et al*, 2015). Moreover, the Vasa N-terminus is strongly methylated, indicating that multivalent interactions with Tudor domain-containing proteins also contribute to nuage assembly (Kirino *et al*, 2010). Additional studies in silkworm revealed that BmVreteno brings piRNA-loaded BmAgo3 and empty Siwi together via their Tudor domains to allow new piRISC-Siwi formation (Nishida *et al*, 2020). This may involve the dimerization of two BmVreteno isoforms (BmVreteno-Long and -Short), where the BmVreteno-Long isoform anchors the RNA target through its unique RRM domain. Thus, BmVreteno also acts in the ping-pong cycle but has an opposite role compared to Krimper and Qin, as it enforces Siwi loading instead of BmAgo3 loading.

Recently, Arif *et al* (2022) reported that gametocyte-specific factor (Gtsf) proteins stimulate the catalytic activity of PIWI proteins. Gtsf proteins act in the piRNA pathway in different species, including flies, silkworms, and mice (Ipsaro & Joshua-Tor, 2022). In *Drosophila*, Gtsf1 is required for piRNA-mediated transcriptional gene silencing, but Gtsf1 is not essential for piRNA biogenesis (Dönertas *et al*, 2013; Ohtani *et al*, 2013). In contrast, mouse Gtsf1 is involved in piRNA amplification and associates with the mouse PIWI proteins Miwi2 and Mili (Yoshimura *et al*, 2018). Moreover, mouse Gtsf1 can enhance the piRNA-directed target cleavage of both Mili and Miwi *in vitro*. Likewise, silkworm Gtsf1 associates with Siwi and was found to enhance slicing activity (Chen *et al*, 2020; Arif *et al*, 2022; Izumi *et al*, 2022). We note that the conditional cleavage by PIWI proteins, which is dependent on Gtsf, provides an interesting possibility to restrict target cleavage to conditions in which an empty PIWI protein may be available to accept a cleavage product and to prevent RNA cleavage in the absence of such empty PIWI proteins. However, it is not known how the Gtsf-piRISC complex is brought in physical proximity with empty PIWI and target RNA.

In this study, we show that silkworm Gtsf1-like (BmGtsf1L), a Gtsf1 paralog, binds piRNA-loaded BmAgo3. The BmGtsf1L-BmAgo3 piRISC interaction is stimulated by BmVreteno, a protein known to aid Siwi loading following BmAgo3-mediated target cleavage. Surprisingly, we find that BmGtsf1L and BmAgo3 bind to the same eTudor domain of BmVreteno. Using AlphaFold predictions, we uncover a novel binding interface on this eTudor domain that additionally accommodates BmGtsf1L binding. Thus, a single eTudor domain within BmVreteno can serve as a molecular scaffold and interconnect BmGtsf1L and piRISC-BmAgo3 to allow efficient target cleavage only within a context that enables Siwi loading.

Results

BmGtsf1L associates with piRNA-loaded BmAgo3

A previous study showed that Gtsf1 is involved in piRNA-regulated sex determination and transposon silencing in the silkworm (Chen *et al*, 2020). The role of its paralog, BmGtsf1L, however, remained

 © 2023 The Authors

elusive. Alignment of Gtsf proteins from different species shows that BmGtsf1L possesses two conserved CHHC-type zinc (Zn) fingers followed by a short C-terminal tail (Fig EV1A). To find potential binding partners of BmGtsf1L, we transiently expressed HA-tagged BmGtsf1L in BmN4 cells and performed immunoprecipitation followed by quantitative mass spectrometry (IP/qMS). Interestingly, many of the enriched proteins, such as BmAgo3, BmVreteno, and Siwi, are known to play a role in piRNA biogenesis (Fig 1A and Dataset EV2). Next, we transiently expressed HA-BmGtsf1L together with FLAG-tagged BmAgo3, BmVreteno, or Siwi and confirmed that these candidates interact with BmGtsf1L, both in the presence or absence of RNA (Fig 1B). Endogenous BmAgo3 is also associated with transiently expressed BmGtsf1L (Fig 1C).

Next, we generated an anti-BmGtsf1L monoclonal antibody, which detected both endogenous as well as epitope-tagged BmGtsf1L (Fig EV1B). Despite the low expression levels of BmGtsf1L, we could detect endogenous BmGtsf1L specifically in BmAgo3 precipitates (Fig 1D). Unfortunately, the BmGtsf1L antibody was not suitable for immunoprecipitation assays and did not function in immunostainings. To be able to study BmGtsf1L function in further detail, we generated a BmN4 cell line stably expressing HA-BmGtsf1L-eGFP. Using this stable cell line, we confirmed that BmGtsf1L is mostly enriched in BmAgo3 IPs and hardly in Siwi IPs (Fig 1E). *Vice versa*, BmAgo3 and Siwi were both co-precipitated with BmGtsf1L, and again, we observed a stronger enrichment for BmAgo3 (Fig 1F). Using stable cell lines expressing epitope-tagged PIWI proteins (Fig EV1C), we could show that the increased affinity of BmAgo3 for BmGtsf1L was not due to differences in PIWI antibody specificities (Fig EV1D). Moreover, BmSpn-E and BmQin were also co-purified by BmGtsf1L, confirming our initial IP/qMS hits (Fig 1A and F).

To reveal the loading status of endogenous BmAgo3 that associates with BmGtsf1L, we performed BmGtsf1L immunoprecipitation followed by small RNA sequencing. BmGtsf1L-associated small RNA profiles resembled those of BmAgo3-bound small RNAs, showing a clear, defined peak of 27 nucleotides in size, a strong sense bias, and enrichment for adenine at the 10th position (Figs 1G and H, and EV1E). Together, these results indicate that BmGtsf1L associates with piRNA-loaded BmAgo3.

Interestingly, BmVreteno has been shown to also interact with piRNA-loaded BmAgo3, and to do so as a dimer (Nishida *et al*, 2020). Using an independently generated anti-BmVreteno antibody that also detects the BmVreteno-Long (L) and BmVreteno-Short (S) isoforms (Fig EV1F–H), we confirm that BmVreteno retrieves BmAgo3 (Fig EV1I). Consistently, both BmVreteno isoforms were also found in BmAgo3 precipitates (Fig EV1J). Next, we assessed the interaction between BmGtsf1L and endogenous BmVreteno. This revealed that BmGtsf1L also brings down both isoforms of endogenous BmVreteno (Fig 1I). Likewise, BmGtsf1L is also co-precipitated by BmVreteno (Fig 1J). Together, these data suggest that BmVreteno, BmGtsf1L, and piRNA-loaded BmAgo3 form a complex.

BmGtsf1L resides in BmAgo3 bodies

Nishida *et al* (2020) recently described that the formation of BmAgo3 bodies is dependent on BmVreteno. To further analyze whether BmGtsf1L also resides in BmAgo3 bodies we exploited fluorescent lifetime imaging microscopy (FLIM). Using this technique, we observed the co-localization of BmGtsf1L to BmAgo3-BmVreteno

Figure 1. BmGtsf1L associates with piRNA-loaded BmAgo3.

A Anti-HA immunoprecipitation on BmN4 cell lysates where either HA-BmGtsf1L or HA-eGFP was ectopically expressed. The experiment was performed using technical duplicates to perform quantitative mass-spectrometry-based detection of peptides using stable dimethyl isotope labeling. A scatterplot showing the $\log_2$ converted normalized ratio data for the individual label pairs. The threshold was set to 2-fold enrichment; known piRNA factors are indicated (red dots) as well as the bait protein (BmGtsf1L, green dot).

B Anti-HA immunoprecipitation on BmN4 lysates made from cells that were transfected with the indicated constructs either in presence or absence of RNase A/T1. BmGtsf1L was immunoprecipitated followed by Western blot detection using the indicated antibodies. Expression of 3xFLAG-eGFP served as a negative control.

C Anti-HA immunoprecipitation of HA-BmGtsf1L or HA-eGFP from BmN4 cell lysates followed by immunodetection of endogenous BmAgo3. Anti-tubulin probing served as a loading control.

D Immunoprecipitation using the indicated endogenous antibodies or using non-immune serum (n.i.) as a control in the presence or absence of naïve BmN4 cell lysates. Oriole stain was used to detect the immunopurified BmAgo3 and Siwi complexes, whereas retrieval of endogenous BmGtsf1L was verified by Western blot.

E Immunoprecipitation of endogenous BmAgo3 and Siwi complexes on BmN4 cell lysates from the HA-BmGtsf1L-eGFP stable cell line, followed by Western blot detection using the indicated antibodies.

F GFP (BmGtsf1L) or control (Ctrl) immunoprecipitation on BmN4 cell extracts stably expressing HA-BmGtsf1L-eGFP was followed by Western blot detection using the indicated antibodies.

G Small RNA size profiles of input samples and from anti-HA immunopurified complexes. Immunoprecipitations were performed in technical duplicates on BmN4 cell lysates from cells that were transiently transfected, denoted in the two lines on the right-hand panels.

H Violin plot showing the $\log_2$ transformed strand bias of sense to antisense small RNAs from input and IP samples of technical duplicates. The mean strand bias is indicated with the color code, where negative and positive values represent antisense and sense bias, respectively. The boxplot inside the data distribution shows the summary of the data as follows: The top and bottom of the embedded box represent the 75th and 25th percentile of the distribution, respectively, and the line inside the box represents the median; the lines extend to the smallest or largest observation that falls within a distance of the inter-quartile range (IQR), Q1–1.5 × IQR or Q3 + 1.5 × IQR respectively.

I GFP (BmGtsf1L) and control immunoprecipitation, followed by Western blot detection of endogenous BmVreteno using an antibody that detects the two BmVreteno isoforms.

J Reciprocal immunoprecipitation using endogenous anti-BmVreteno antibody or rabbit IgG as an isotype control, followed by immunodetection using the indicated antibodies.

K Fluorescence lifetime imagining of BmN4 cells co-transfected with eGFP-BmVreteno, BmGtsf1L- mOrange2, and mCardinal-BmAgo3. Inset shows the zoom-in of the boxed area. Two representative images of two biological experiments are shown. Scale bars:10 μm. Plots on the right show the intensity gray values for each channel of the line that has been drawn in the inset frame. Quantification data can be found in Dataset EV4.

Source data are available online for this figure.

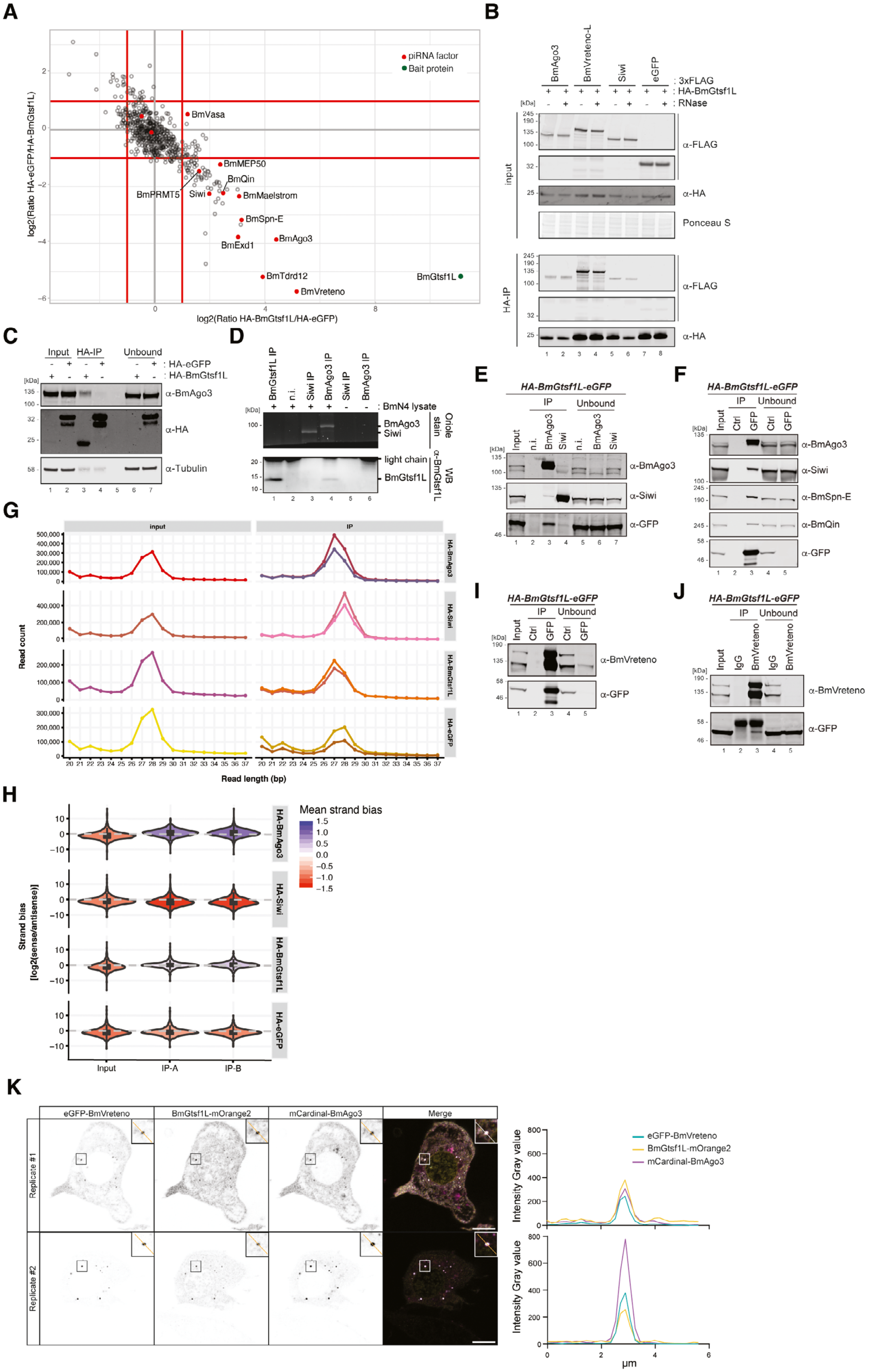

Figure 1.

    © 2023 The Authors

marked granules (Fig 1K). Single transfection of the individual proteins served as controls and revealed the specificity of each fluorescently tagged protein (Fig EV2A).

Interdependence of BmVreteno-BmAgo3-BmGtsf1L interaction

Next, we assessed the consequences of BmGtsf1L knockdown on the interactions we described above. Knockdown of BmGtsf1L followed by BmVreteno immunoprecipitation revealed that the interaction between BmVreteno and BmAgo3 does not require BmGtsf1L (Fig 2A). *Vice versa*, both isoforms of BmVreteno were also still retrieved by BmAgo3 following BmGtsf1L depletion (Fig 2B). These results indicate that the interaction between BmVreteno and BmAgo3 does not require BmGtsf1L.

To test whether BmAgo3 affects the interaction between BmGtsf1L and BmVreteno, we analyzed their association following efficient BmAgo3 knockdown. This revealed that the interaction between BmGtsf1L and BmVreteno was reduced but not fully abrogated (Fig 2C). Given that no BmAgo3 was detected in this BmGtsf1L IP, these results suggest that BmAgo3 enhances, but is

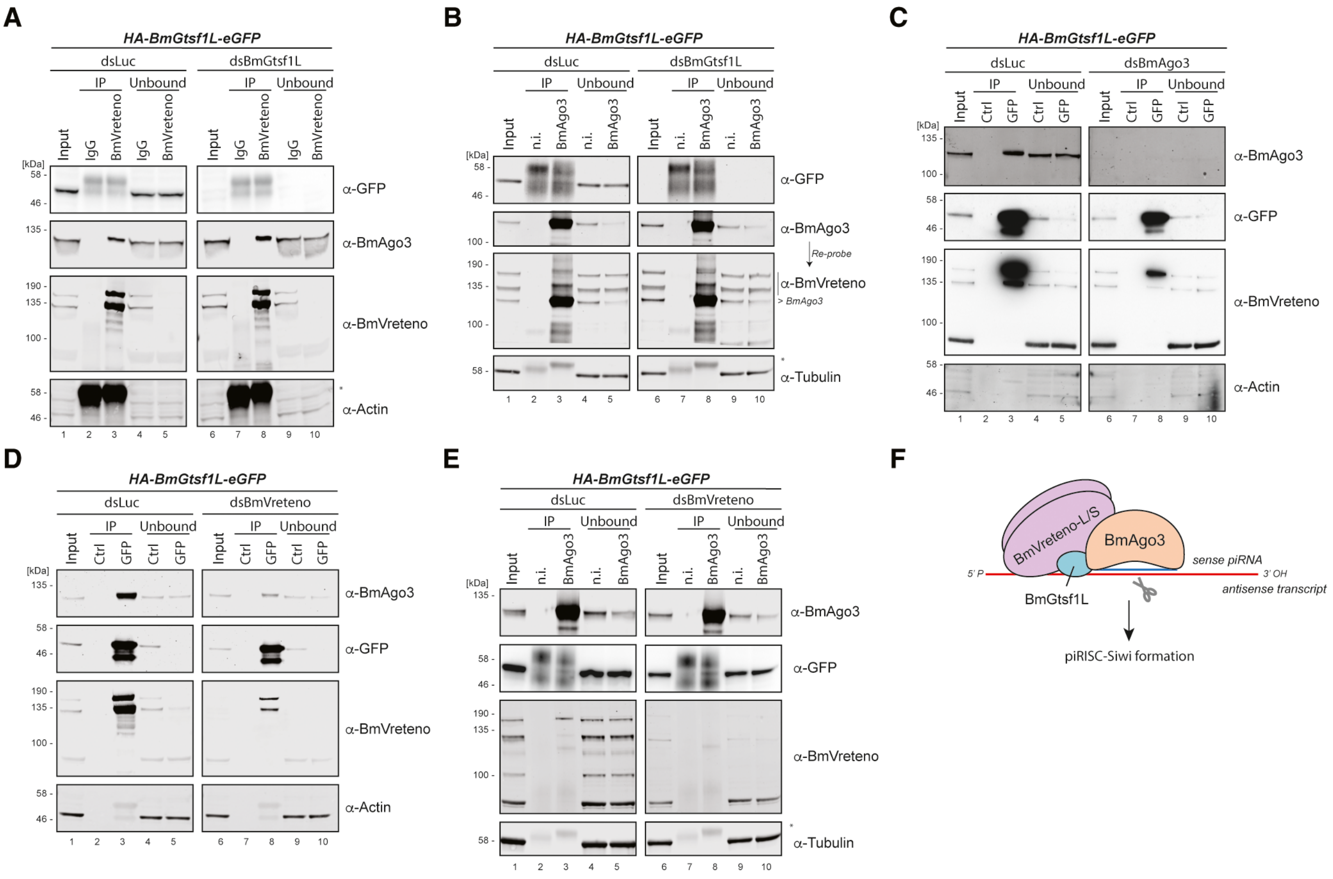

Figure 2. Interdependence of BmVreteno-BmAgo3-BmGtsf1L interaction.

dsRNA-mediated gene depletion on BmN4 cells, which stably express HA-BmGtsf1L-eGFP, followed by immunoprecipitation and Western blot detection.

A Luciferase (dsLuc) control knockdown and BmGtsf1L (dsBmGtsf1L) depletion, followed by IgG control or anti-BmVreteno immunoprecipitations and detection of retrieved proteins by Western blot using the indicated antibodies. Anti-actin detection served as the loading control. An asterisk indicates detection of the antibody light chain.

B Knockdown in BmN4 cells as described in panel (A), followed by non-immune (n.i.) serum control IP or anti-BmAgo3 IP and detection of retrieved proteins by Western blot. Anti-tubulin probing was used as a loading control. An asterisk indicates detection of the antibody light chain.

C Knockdown of luciferase (dsLuc) or BmAgo3 (dsBmAgo3) in BmN4 cells. Immunoprecipitation using GFP (BmGtsf1L) or control (Ctrl) magnetic beads, followed by Western blot detection using the indicated antibodies. Anti-actin probing was performed as a loading control.

D Immunoprecipitation and Western blot detection were performed as described in panel (C), but on BmN4 cell extracts from which endogenous BmVreteno was depleted by dsRNA transfection.

E Knockdown of endogenous BmVreteno, followed by immunoprecipitation using anti-BmAgo3 antibodies or non-immune serum as a control. Western blot detection of precipitated proteins was performed using the indicated antibodies and detection of anti-tubulin served as a loading control. Asterisk indicates detection of the antibody light chain.

F Model on the interconnection between BmGtsf1L, BmAgo3, and BmVreteno. The majority of BmGtsf1L is found in complex with BmVreteno. BmVreteno, which can stimulate the BmGtsf1L-BmAgo3 interaction, whereas BmAgo3 also fosters the BmGtsf1L-BmVreteno interaction. BmVreteno exists as a long (L) and short (S) isoform, which can interact with each other (Nishida *et al*, 2020) and is therefore schematically depicted as a heterodimer.

Source data are available online for this figure.

© 2023 The Authors

not essential for the interaction between BmGtsf1L and BmVreteno. Finally, we tested the effects of BmVreteno depletion on the BmAgo3-BmGtsf1L interaction. While the dsRNA treatment strongly affected BmVreteno levels, we were not able to fully eliminate it, as evidenced by its presence in the BmGtsf1L IPs. Nevertheless, RNAi against BmVreteno strongly diminished the amount of BmAgo3 that was brought down by BmGtsf1L (Fig 2D), indicating that BmVreteno stimulates the interaction between BmGtsf1L and BmAgo3. We note, however, that in reciprocal BmAgo3 immunoprecipitations, small amounts of BmGtsf1L could be retrieved, independent of BmVreteno knockdown status (Fig 2E). In this experiment no residual BmVreteno was detected in the IPs. We conclude that a small fraction of BmGtsf1L binds BmAgo3 independent of BmVreteno, but that the majority of BmGtsf1L is found in complex with BmVreteno and that this stimulates the BmGtsf1L-BmAgo3 interaction (Fig 2F).

BmAgo3 interacts with BmVreteno eTD1 via methylated arginine residues

BmVreteno contains two Tudor domains (TDs) that are confidently predicted by Pfam and SMART Hidden Markov Models (HMMs) (Letunic *et al*, 2021; Mistry *et al*, 2021). A third match of the Pfam Tudor HMM around residue 500 exceeded the e-value threshold and thus was not significant. Alignment of the two confidently predicted Tudor domains to those of *Drosophila* Tudor-SN and Tudor-eTD11, for which crystal structures have been resolved (Friberg *et al*, 2009; Liu *et al*, 2010), indicates that BmVreteno TD1 contains an aromatic cage (Fig EV2B). It is well known that aromatic cages within TD domains can bind to methylated arginines of client proteins and thereby mediate protein–protein interactions (Siomi *et al*, 2010; Chen *et al*, 2011). When co-expressing BmVreteno with a BmAgo3 variant that cannot be methylated at its N-terminus (5RK), we lost interaction between both (Fig EV2C), suggesting that arginine methylation is a prerequisite for its association with BmVreteno. This is in line with previous work, which showed that the aromatic cage of TD1 is involved in BmAgo3 interaction (Nishida *et al*, 2020).

In addition, a BmAgo3 piRNA-loading defective mutant (YK > LE) also revealed a strong loss of interaction with BmVreteno (Fig EV2C), which is in line with the observation that unloaded BmAgo3 does not co-localize with BmVreteno and fails to form BmAgo3 bodies (Nishida *et al*, 2020). This could indicate that BmAgo3 becomes methylated only following piRNA-binding, which has been observed for *Drosophila* Aubergine (Webster *et al*, 2015; Huang *et al*, 2021). Notably, a BmAgo3 slicing mutant (DADH) does not show loss of interaction with endogenous BmVreteno (Fig EV2C). Taken together, our data, combined with the findings of Nishida *et al* (2020), suggests that the aromatic cage of the BmVreteno TD1 domain mediates the interaction with the methylated N-terminus of BmAgo3.

The BmGtsf1L C-terminus establishes an interaction with BmVreteno

To understand how BmGtsf1L binds to BmVreteno, we tested which region of BmGtsf1L interacted with endogenous BmVreteno. At the same time, we also probed for BmAgo3. A BmGtsf1L fragment missing the N-terminal part, including the two CHHC Zn fingers, could still retrieve BmVreteno as well as BmAgo3 (Fig 3A and B). By contrast, deletion of the likely disordered BmGtsf1L C-terminus completely abolished the interaction with BmVreteno, while it allowed some interaction with BmAgo3 (Fig 3A and B). Studies in *Drosophila* showed that aromatic residues within the C-terminus of Gtsf1 regulate Piwi binding (Dönertas *et al*, 2013; Ohtani *et al*, 2013). Therefore, we checked for the presence of aromatic residues within BmGtsf1L and studied how the mutagenesis of these residues would affect its interaction with either BmAgo3 or BmVreteno. The BmGtsf1L tyrosine residue mutant (Y88A) retrieved BmAgo3 and BmVreteno to a similar extent as wildtype BmGtsf1L, whereas mutation of the conserved tryptophan residue (W99A) affected the BmAgo3 interaction and completely abrogated the interaction with BmVreteno (Figs 3A and C, and EV1A). The BmGtsf1L double point mutant (YW > AA) displayed similar effects when compared to the W99A single mutant. We could also show that BmGtsf1L (W99A) remained uniformly distributed in the nucleus and cytoplasm, even though BmAgo3 granules were still present (Fig 3D and E). We thus identified the conserved tryptophan residue (W99) within the C-terminal tail of BmGtsf1L to be essential for interaction with BmVreteno and to enhance binding to BmAgo3.

BmVreteno directly interacts with BmGtsf1L

The above results prompted us to test the hypothesis that BmVreteno and BmGtsf1L interact directly. Using an *E. coli* expression system, we succeeded in the expression and purification of full-length BmVreteno-L, BmVreteno-S, and BmGtsf1L. Notably, GST-BmVreteno was eluted as multimeric proteins from gel filtration columns and associated with nucleic acids (Fig EV3A–C). Using these proteins in GSH pull-down assays, we could show that BmGtsf1L interacts directly with both isoforms of BmVreteno (Fig 3F). Furthermore, we could recapitulate the effects of the mutations described above on the BmVreteno-BmGtsf1L interaction *in vitro*: recombinant BmGtsf1L-W99A failed to interact with BmVreteno (Fig 3G). We conclude that the C-terminal end of BmGtsf1L is sufficient to bind directly to BmVreteno and that BmGtsf1L-W99 plays a crucial role in this interaction.

BmGtsf1L binds to BmVreteno eTD1

We next analyzed which region of BmVreteno is involved in its interaction with BmGtsf1L. Using truncation analysis, we found that the C-terminal region of BmVreteno, containing the two PFAM/SMART predicted TDs, was required (Fig EV3D and E). However, purification of fragments containing individual predicted TD domains to probe binding with BmGtsf1L failed. To improve fragment design, we turned to AlphaFold as a novel artificial intelligence-based tool for protein structure prediction. To our surprise, AlphaFold confidently predicted three extended Tudor domain folds within full length BmVreteno, which are also referred to as TSN folds (Fig 4A and Dataset EV1) (Liu *et al*, 2010). Hereafter, we refer to these three domains as AF-eTD0, 1, and 2, where AF-eTD0 corresponds to the newly identified eTD domain. The prediction of this additional Tudor domain is in line with IUPred predictions suggesting that this region is structured (Fig 4A). It also overlaps with the nonsignificant Tudor HMM match from SMART/Pfam mentioned earlier. In addition, AlphaFold predicted with high confidence the structures and boundaries of the RRM and MYND domains

 © 2023 The Authors

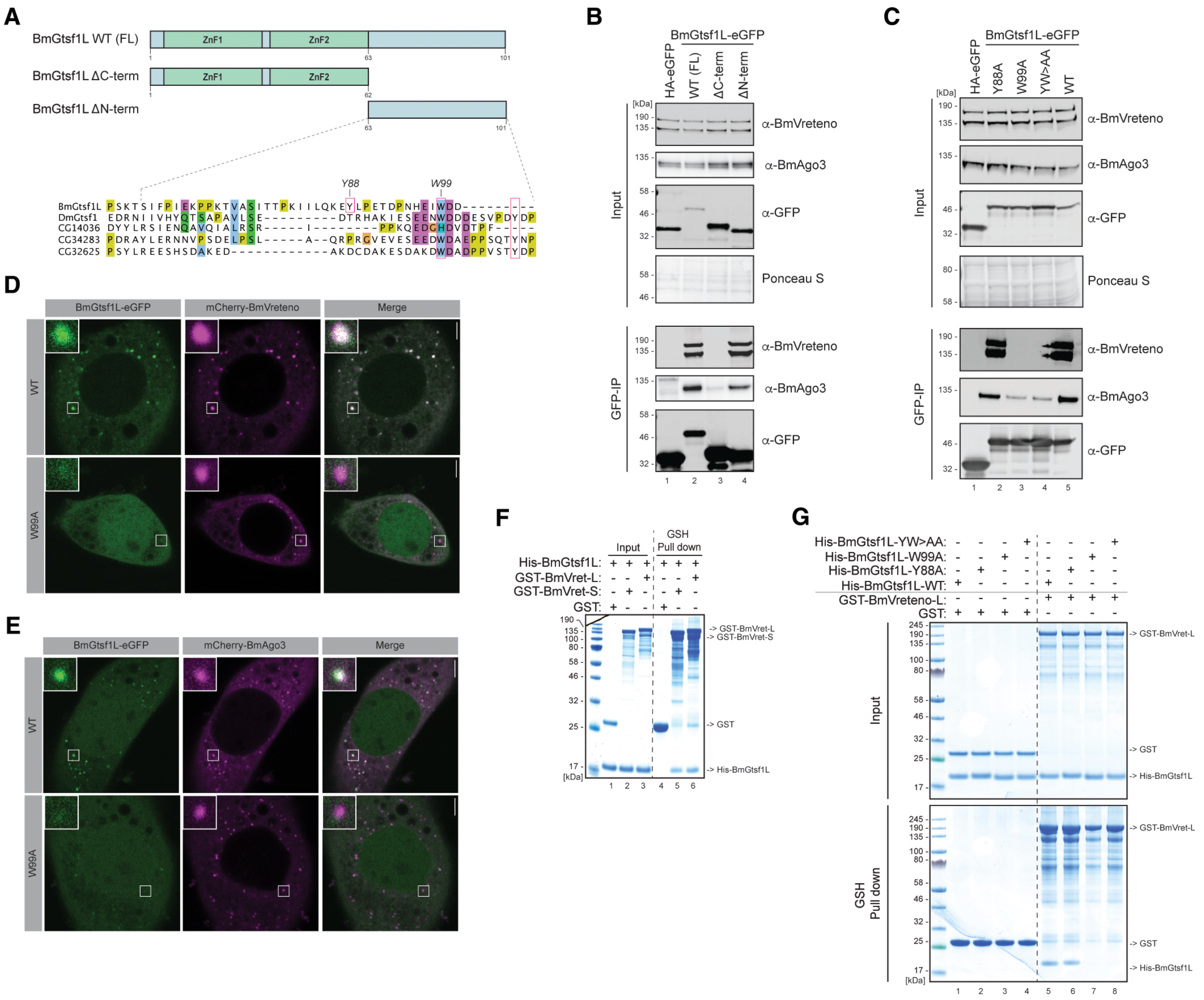

Figure 3. The BmGtsf1L C-terminus establishes a direct interaction with BmVreteno.

A Overview of BmGtsf1L domain architecture with two N-terminally located zinc fingers (ZnF1 and ZnF2, respectively). The two deletion variants used in panel (B) to address binding to BmAgo3 and BmVreteno are also depicted (top). Alignment of BmGtsf1L to GTSF proteins from *Drosophila*. Conserved tryptophan (W) and tyrosine (T) residues of DmGtsf1 that were shown to be involved in Ago3 interaction are boxed in magenta (Dönertas *et al*, 2013). BmGtsf1L contains another aromatic residue (Y88) in its C-terminus (small magenta box), which is not conserved. Clustal Omega alignment was processed with Jalview software (bottom).

B GFP-IP (BmGtsf1L) on BmN4 cell extracts from cells that were transfected with full length (FL) BmGtsf1L-eGFP and with their deletion variants. Transfection of HA-eGFP served as a control. Western blot was performed with indicated antibodies and Ponceau S staining served as a loading control.

C Same as in panel (B) but now BmGtsf1L-eGFP mutants, in which aromatic residues were substituted with alanine, were transfected.

D Single-plane confocal micrographs of BmN4 cells transfected with BmGtsf1L-eGFP wildtype (WT) or the W99A mutant together with mCherry-BmVreteno. Inset shows the zoom-in of the boxed area. Scale bars – 4 µm.

E Single-plane confocal micrographs of BmN4 cells transfected with BmGtsf1L-eGFP wildtype (WT) or the W99A mutant together with mCherry-BmAgo3. Inset shows the zoom-in of the boxed area. Scale bars – 4 µm.

F Analysis of the interaction between BmVreteno and BmGtsf1L by GSH pull-down assays. GST alone or GST-BmVreteno L/S was incubated with His-BmGtsf1L. Input and elution fractions were analyzed by SDS-PAGE followed by Coomassie staining.

G *In vitro* GSH pull-down assay for GST alone or for GST-BmVreteno-L incubated with His-tagged BmGtsf1L variants. Proteins from the SDS-PAGE gel were detected by Coomassie staining.

Source data are available online for this figure.

further upstream of the three eTD domains (Fig 4B). As indicated by the predicted aligned error matrix, AlphaFold is very uncertain about the relative orientation of the RRM, MYND, and AF-eTD0 domain to the rest of the protein (Fig 4B). This suggests that they are unlikely to establish intramolecular contacts with other regions in BmVreteno, while AlphaFold is somewhat more certain

about the predicted relative orientation of AF-eTD1 and AF-eTD2 to each other. Based on the predicted AF-eTD boundaries, we designed novel fragments carrying individual eTD domains to test if any of these AF-eTDs mediate the binding to BmGtsf1L. BmN4 cells were transfected with individual HA-eGFP-tagged AF-eTDs of BmVreteno together with mCherry-3xFLAG-BmGtsf1L. Only BmVreteno AF-eTD1 was retrieved in BmGtsf1L immunoprecipitations (Fig 4C). Likewise, recombinant BmGtsf1L was only co-precipitated with now purifiable GST-tagged BmVreteno-AF-eTD1 *in vitro*, while AF-eTD0 and AF-eTD2 could not bind BmGtsf1L (Fig 4D).

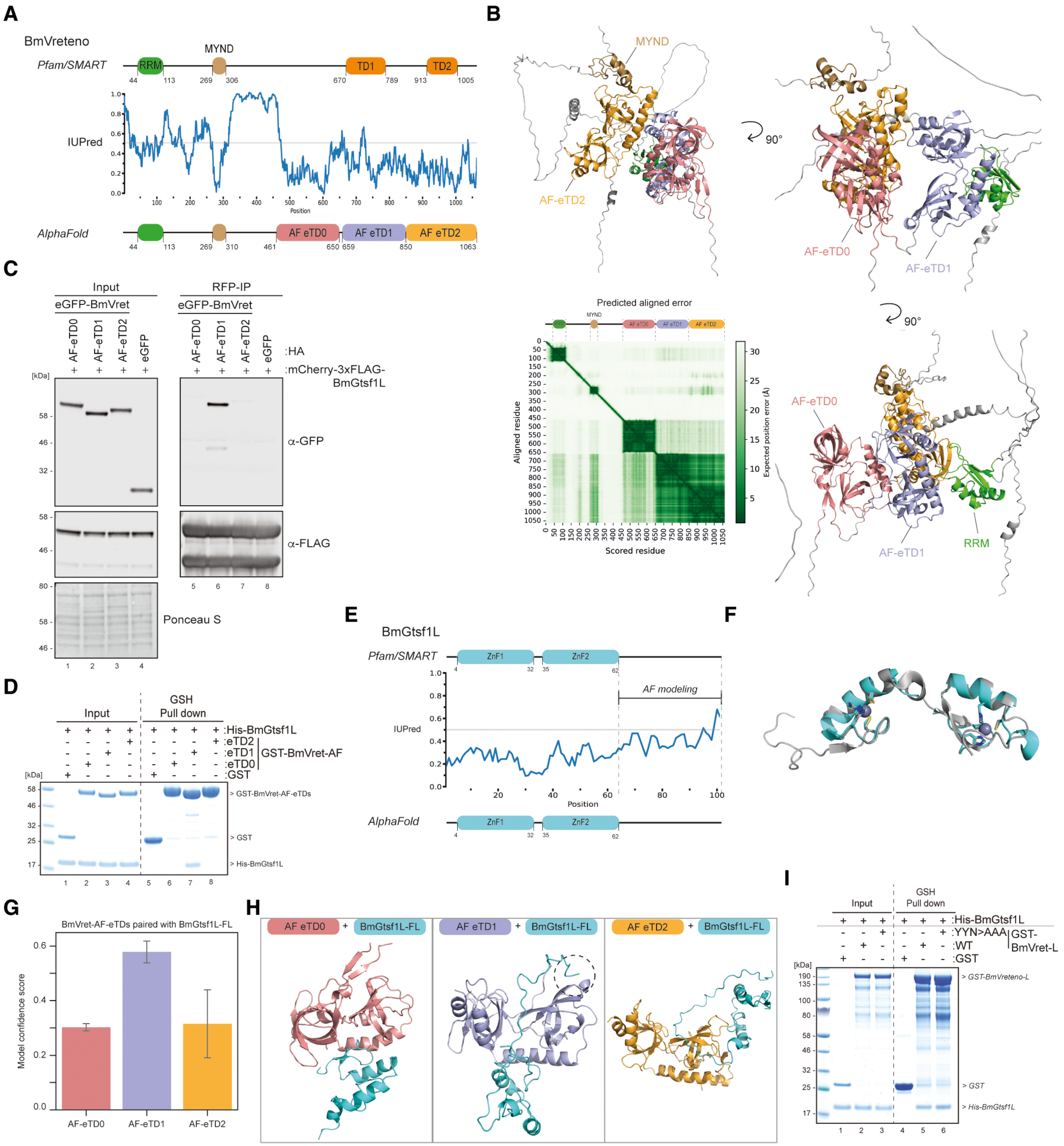

Figure 4.

    © 2023 The Authors

Figure 4. BmVreteno AF-eTD1 interacts with a C-terminal motif in BmGtsf1L.

A Domain organization of BmVreteno is based on Pfam/SMART annotations (top) or domain annotations from AlphaFold predictions (bottom). IUPred predictions (center) indicate structural disorder propensities for BmVreteno (values > 0.5 indicate disorder). Disorder scores and amino acid positions are shown on the *X*-axis and *Y*-axis, respectively. Abbreviations: RRM = RNA recognition motif; MYND = Myeloid translocation protein 8, Nervy and DEAF-1; TD = Tudor domain; AF-eTD = AlphaFold predicted extended Tudor domain.

B AlphaFold predicted the structure of full-length BmVreteno shown from different angles. Individual domains within the displayed structure are color coded as in panel (A). The BmVreteno domain organization is displayed on top of the PAE matrix. The PAE plot displays the scored residues and aligned residues on the *X*-axis and *Y*-axis, respectively. The expected position error in angstroms (Å) is color coded, where a dark green color indicates low PAE (high confidence) and a white color indicates high PAE (low confidence).

C Transfection of BmN4 cells with mCherry-3xFLAG-BmGtsf1L together with individual eTD domains of BmVreteno, carrying an HA-eGFP tag. The transfection of HA-eGFP served as a control. An RFP immunoprecipitation was performed on BmN4 lysates, and input as well as elution samples were resolved by SDS-PAGE. Proteins were detected by Western blot using the indicated antibodies, and Ponceau S staining served as a loading control.

D Analysis of the interaction between individual eTDs of BmVreteno and BmGtsf1L by GSH pull-down assays. GST alone or GST-BmVreteno-AF-eTDs were incubated with His-BmGtsf1L. Input and elution fractions were analyzed by SDS-PAGE, followed by Coomassie staining.

E Domain organization of BmGtsf1L is based on Pfam/SMART annotations (top) or domain annotations from AlphaFold predictions (bottom). IUPred predictions (center) indicate structural disorder propensities for BmGtsf1L. Disorder scores and amino acid positions are shown on the *X*-axis and *Y*-axis, respectively. The more disordered C-terminal tail was used for AlphaFold predictions (related to Fig 5). Abbreviation: ZnF1 = zinc finger 1; ZnF2 = zinc finger 2.

F Superimposition of the structure of the Zn fingers from MmGtsf1 (in gray, PDB: 6X46) with the predicted structure of the Zn fingers of BmGtsf1L by AlphaFold (in cyan). Zinc-binding residues within MmGtsf1 and BmGtsf1L that coordinate zinc ion-binding are displayed as sticks.

G Bar chart showing the mean of the different model confidence scores that were obtained from AlphaFold predictions (*Y*-axis) using individual AF-eTDs of BmVreteno (*X*-axis) that were paired with full-length BmGtsf1L (error bars indicate the standard deviation of the five predicted models).

H AlphaFold-predicted structures for each individual eTD of BmVreteno with full-length BmGtsf1L. The inset in the middle panel shows that the C-terminal tail of BmGtsf1L establishes contacts with the ordered structure of BmVreteno AF-eTD1.

I Analysis of the interaction between BmVreteno-L full-length wildtype (WT) and the aromatic cage mutant (YYN > AAA) with BmGtsf1L by GSH pull-down assays. GST alone, or GST-BmVreteno-L, was incubated with His-BmGtsf1L. Input and elution fractions were analyzed by SDS-PAGE followed by Coomassie staining.

Source data are available online for this figure.

BmVreteno AF-eTD1 interacts with a C-terminal motif in BmGtsf1L

Results from the previous sections indicate that a region around the W99 residue in the disordered C-terminal tail of BmGtsf1L can bind to AF-eTD1, pointing to the possibility that this interaction is mediated by a so-called short linear motif-folded domain interaction (Van Roey *et al*, 2014). Various reports suggest that AlphaFold has some ability to predict domain-motif interfaces between two submitted protein sequences (Akdel *et al*, 2022; Tsaban *et al*, 2022). However, prior to probing AlphaFold for interface prediction between BmVreteno and BmGtsf1L, we first tested whether AlphaFold could predict the structure of full-length BmGtsf1L with high confidence. AlphaFold confidently predicted the two N-terminal Zn-finger domains and a disordered C-terminal tail in line with Pfam/SMART domain annotations and IUPred disorder propensity predictions (Fig 4E and Dataset EV1). The superimposition of the BmGtsf1L Zn fingers with the resolved structure of mouse Gtsf1 (PDB: 6X46) showed a very similar overall structure (Fig 4F). Each MmGtsf1 Zn finger coordinates the binding of an individual zinc ion (Ipsaro *et al*, 2021). Displaying the zinc-coordinating residues of BmGtsf1L revealed that AlphaFold accurately modeled these residues, despite the fact that AlphaFold cannot model the zinc ions themselves (Fig 4F).

Encouraged by these observations, we submitted full-length BmGtsf1L and full-length BmVreteno for interface prediction by AlphaFold. Unfortunately, predicted structural models were of very low model confidence (at most 0.27) and docked the BmGtsf1L Zn finger domains between AF-eTD0 and AF-eTD2 of BmVreteno in an unlikely mode of binding that also contradicts our experimental results (Dataset EV1).

Next, we submitted sequences of individual AF-eTDs from BmVreteno with the full-length sequence of BmGtsf1L for interface prediction. Interestingly, while predictions involving AF-eTD0 and AF-eTD2 resulted again in low confidence predictions, structural models involving AF-eTD1 resulted in substantially higher model confidences (Fig 4G and Dataset EV1). Inspection of the structural models revealed that AlphaFold predicted binding of a region involving W99 in BmGtsf1L to AF-eTD1 exclusively (Fig 4H), in line with our experimental data. Interestingly, AlphaFold docked W99 of BmGtsf1L into a small hydrophobic pocket of AF-eTD1 that was different from the aromatic cage (see below). Indeed, BmGtsf1L can still interact with BmVreteno *in vitro* when the aromatic cage was disrupted (Fig 4I). This data would suggest that BmAgo3 and BmGtsf1L both bind to BmVreteno AF-eTD1 while using different interaction interfaces on AF-eTD1.

AF predicts BmGtsf1L motif binding to a novel hydrophobic pocket on the BmVreteno AF-eTD1 domain

Despite these encouraging agreements between our experimental data and AlphaFold predictions, the structural models of the interface between AF-eTD1 and full-length BmGtsf1L were still only of moderate model confidence (max. 0.64). To further gain in prediction accuracies, we *in silico* fragmented the unstructured C-terminal tail of BmGtsf1L (39 AA in length), starting off with a fragment of five residues in length at the start, middle, and end of the C-terminal tail and gradually extending these fragments by five residues in each step (Fig 5A). We submitted each fragment individually for interface prediction by AlphaFold with each of the three eTD domains of BmVreteno. This resulted in 72 prediction runs in total (Table EV1). Since the C-terminal fragments were overlapping among each other, we were able to compute the fraction of prediction runs involving a specific pair of residues from BmVreteno and BmGtsf1L where this pair of residues was predicted to be in contact with each other. This computed fraction was visualized as a heatmap between all residues

© 2023 The Authors

from BmVreteno AF-eTD domains that were observed to be at least once in contact with a residue from the C-terminal tail of BmGtsf1L and *vice versa* (Fig 5A). This residue-residue contact heat map revealed a clear hotspot of residues within AF-eTD1 and residues in BmGtsf1L, including W99, and residues close by that were consistently predicted to be in contact with each other (Fig 5A). No such hotspot was observed for AF-eTD0 and AF-eTD2 suggesting that AlphaFold specifically predicted an interface between AF-eTD1 and

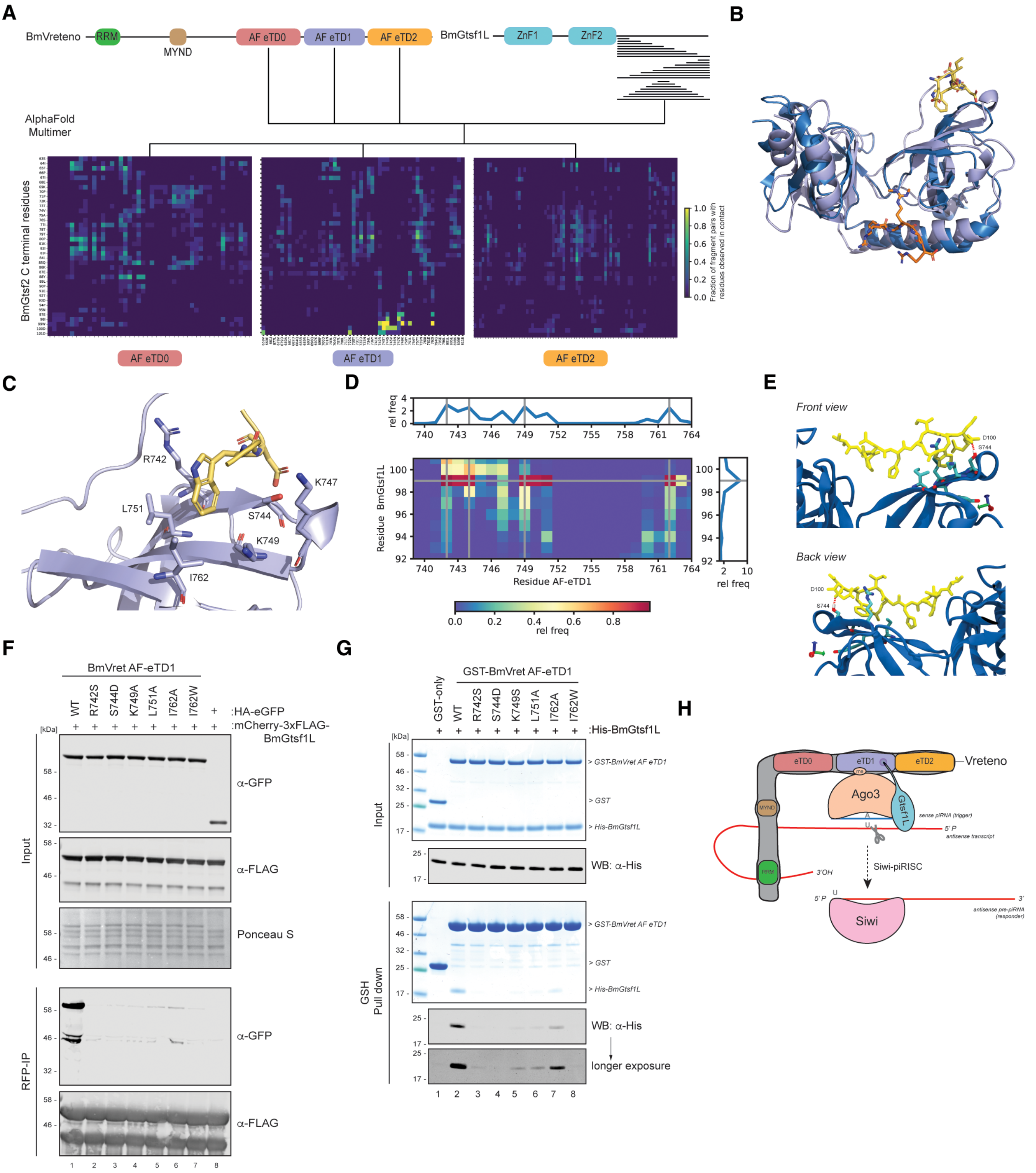

Figure 5.

 © 2023 The Authors

Figure 5. The Gtsf1L motif binds to a novel hydrophobic pocket on the BmVreteno AF-eTD1 domain.

A AlphaFold-based domain organization of BmVreteno and BmGtsf1L and a schematic overview of the fragmentation approach of the C-terminus of BmGtsf1L. BmGtsf1L fragments were paired for interface predictions with AlphaFold with each of the eTDs of BmVreteno (top). The frequency by which a pair of residues, one from BmVreteno and one from BmGtsf1L, was predicted to be in contact with each other among all fragment pairs submitted to AlphaFold that contained this residue pair is visualized as a heatmap for each individual eTD domain of BmVreteno. Only residues of BmVreteno and BmGtsf1L that were at least observed once to be in contact with a partner residue are displayed on the *X* and *Y*-axis, respectively.

B Superimposition of eTudor11 from *Drosophila* Tudor (in dark blue, PDB: 3NTH) crystallized with a peptide containing a methylated arginine residue of Aubergine (orange) with the structural model of AF-eTD1 (light blue) and the C-terminal five residue-long peptide of BmGtsf1L (in yellow). Peptide residues are represented as sticks.

C Zoom-in on the novel hydrophobic binding pocket of BmVreteno AF-eTD1 (light blue) and contacts between the hydrophobic residues (shown as sticks) with BmGtsf1L W99 and D100 residues (shown as yellow sticks).

D Contact map of BmVreteno AF-eTD1 with the BmGtsf1L 10-AA residue peptide, predicted by atomistic molecular dynamics simulations. The plot summarizes 10 runs of one microsecond each. The blue color in the heatmap indicates a low relative frequency of contacts between the BmGtsf1L-BmVreteno residues, and red indicates a high frequency of contacts. Marginal plots that display the relative frequency (rel freq) show the relative probability of a residue interacting with residues from the binding partner, which is the sum of the probability for each column (for the sum of the contacts along the AF-eTD1 sequence) or row (for the sum of the contacts along the BmGtsf1L 10-AA residue peptide sequence).

E Snapshot on the novel hydrophobic binding pocket of BmVreteno AF-eTD1 (blue) and contacts between residues R742, S744, K749, and I762 (shown as sticks) with BmGtsf1L C-terminal 10-AA residues (shown as yellow sticks). The snapshot additionally displays the BmVreteno-S744 residue that can form a hydrogen bond with the backbone carbonyl of BmGtsf1L-D100.

F Anti-RFP (BmGtsf1L) immunoprecipitation from BmN4 lysates made from cells that were transfected with HA-eGFP-tagged BmVreteno AF-eTD1 variants. Cells were co-transfected with mCherry-BmGtsf1L. The transfection of HA-eGFP served as a control. Proteins from input and elution samples were resolved by SDS-PAGE, followed by Western blot detection using the indicated antibodies. Ponceau-S staining served as a loading control.

G *In vitro* GSH pull-down assay for GST alone or for GST-BmVreteno-AF eTD1 variants incubated with His-tagged BmGtsf1L. Proteins from the input and elution fractions are separated by SDS-PAGE and detected by Coomassie staining. For more sensitive detection, a fraction of the same input and elution samples were subjected to gel electrophoresis in parallel followed by Western blot detection using anti-His antibodies.

H Model showing that a novel binding interface on BmVreteno AF-eTD1 facilitates the binding of BmGtsf1L (via the hydrophobic pocket) and BmAgo3 (aromatic cage).

Source data are available online for this figure.

a motif in BmGtsf1L involving W99. Importantly, model confidences reached 0.87 for these fragment pairs (Table EV1).

We superimposed the structural model involving AF-eTD1 and the last 5 residues of BmGtsf1L (Table EV1) with the solved structure of eTD11 of the *Drosophila* Tudor protein in complex with a synthetic peptide representing the methylated arginine residues of Aubergine (PDB: 3NTH, Fig 5B) (Liu *et al*, 2010). This clearly shows that the predicted interface between BmVreteno and BmGtsf1L does not involve the aromatic cage and lies on the opposite site of AF-eTD1. Closer inspection of the interface revealed that W99 of BmGtsf1L is predicted to bind in a hydrophobic pocket formed by the side chains of R742, K747, K749, L751, and I762 of AF-eTD1 (Fig 5C). Furthermore, the conserved D100 of BmGtsf1L is predicted to be in contact with K722 and R742, suggesting charge–charge contacts. This is also true for BmGtsf1L-D101 and BmVreteno-K747 (Fig 5C). To understand why AlphaFold predictions and experimental results suggest that BmGtsf1L motif-binding is specific to the AF-eTD1 domain of BmVreteno, we superimposed the structural models of all three AF-eTD domains. We observed that the described hydrophobic pocket as well as the aromatic cage are specific to AF-eTD1 (Fig EV4A).

To gain further confidence in the stability of the predicted interface between BmGtsf1L and BmVreteno, we performed atomistic molecular dynamics simulations using the AlphaFold structural model involving AF-eTD1 of BmVreteno and the ten last residues of BmGtsf1L as starting point. In nine out of ten 1 μs simulations, we observed that W99 anchors the BmGtsf1L motif into the predicted hydrophobic pocket of AF-eTD1 (Fig 5D). However, in one of the ten simulations, W99 moves away from the shallow hydrophobic pocket suggesting that additional contacts between both proteins are required to further stabilize the interaction. Our contact analysis of the ten simulation runs demonstrate that the flanking residues I98, D100, and D101 also contribute to anchoring the BmGtsf1L motif, whereas the remaining part of the BmGtsf1L peptide forms fewer contacts with the AF-eTD1 domain and is highly dynamic (Fig 5D and E). On average W99 is interacting with BmVreteno residues R742 and K749 as well as I762. Movie EV1, which visualizes one of the ten trajectories, shows how W99 can be "sandwiched" between the side chains of R742 and K749. The simulations also suggested an important contribution of S744 in AF-eTD1 to BmGtsf1L motif-binding by mostly interacting with W99 but also with D100 (Fig 5E). S744 forms transient hydrogen bonds with I98 and D100 of BmGtsf1L (Figs 5E and EV4B and C), with the proton of the S744 OH group interacting both with the carboxyl group of the D100 side chain and the D100 backbone carbonyl (Fig EV4C). Interestingly, W99 and D100 also display high conservation scores across orthologous Gtsf sequences (Fig EV1A), suggesting that this may be a conserved mode of binding.

Experimental verification of the AF-predicted BmGtsf1L-BmVreteno interface

We set out to further probe this predicted mode of binding using mutagenesis. To this end, we selected residues within AF-eTD1 that contribute to forming the hydrophobic pocket or/and mediate interaction with the aspartate (D100, D101) residues in the BmGtsf1L motif. Individual point mutations (R742S, S744D, K749S, L751A, and I762A) were generated for which we hypothesized that these would perturb the formation of the hydrophobic pocket. We also generated a point mutant (I762W) in which the hydrophobic pocket would be filled and as such would sterically hinder the binding of BmGtsf1L. These mutations were also designed such that an overall impact on the stability and folding of AF-eTD1 should be minimal. Immunoprecipitations on BmN4 cell extracts derived from cells that

© 2023 The Authors

were co-transfected with BmGtsf1L and the panel of AF-eTD1 mutants, showed that the interaction between BmGtsf1L and AF-eTD1 was abolished in all the variants that we tested (Fig 5F). Using recombinant GST-tagged AF-eTD1 variants in GSH pull-down assays, we also observed that the direct interaction between AF-eTD1 and BmGtsf1L was strongly impaired by these mutations (Fig 5G).

Finally, we studied the effect of these mutations on the BmGtsf1L-BmVreteno interaction in the context of full-length proteins. We assessed these interactions through co-IP experiments, as well as via subcellular localization. Mutations within the hydrophobic pocket on AF-eTD1 were introduced into full -length BmVreteno and were co-transfected with BmGtsf1L. By microscopy, it was apparent that all the hydrophobic pocket mutants resulted in nuclear BmGtsf1L localization (Fig EV4D). This indicates a loss of binding, since BmVreteno wildtype overexpression results in BmGtsf1L exclusion from the nucleus. In addition, we find that BmGtsf1L is still present in granules of BmVreteno mutants, which could be explained by the presence of endogenous BmVreteno that can form a complex with the transiently expressed BmVreteno mutant and suffices to recruit a small fraction of BmGtsf1L. Mutation of the aromatic cage did not result in nuclear BmGtsf1L (Fig EV4D), consistent with it not playing a role in BmGtsf1L binding.

Immunoprecipitations of BmGtsf1L revealed that single point mutations within the hydrophobic pocket had little effect on BmVreteno retrieval (Fig EV4E). More significant reduction in binding could be observed for the serine (S744D) and isoleucine (I762W) mutations that also showed the strongest effects in our *in vitro* assay (Figs EV4E and 5E). A stronger loss of interaction was observed when generating double mutants for the hydrophobic pocket residues that initially showed no or marginal effects when compared to wildtype BmVreteno. We note that the full-length BmVreteno that we transiently expressed likely still dimerizes or oligomerizes with endogenous BmVreteno, which in turn would still be able to interact with endogenous BmAgo3 (via the aromatic cage). This might lead to the observed residual BmGtsf1L binding. Taken together, our data reveal a novel binding interface on BmVreteno AF-eTD1 that facilitates the simultaneous binding of BmGtsf1L and BmAgo3 (Fig 5H).

Discussion

In this study, we show that one of the eTudor domains of BmVreteno acts on its own as a molecular scaffold to bring piRNA-loaded BmAgo3 and BmGtsf1L in close proximity. BmGtsf1L slightly stimulates BmAgo3-directed cleavage of an RNA target (Arif *et al*, 2022; Izumi *et al*, 2022). Concurrently, BmVreteno provides an environment that promotes the handover of the cleaved target to empty Siwi (Murakami *et al*, 2021). Therefore, we propose that the interactions that we identify may help to restrict BmAgo3 cleavage to a molecular surrounding in which its cleavage products can fuel piRNA biogenesis, and to prevent futile BmAgo3 cleavage events. As the targets of BmAgo3 are antisense transcripts, its cleavage activity will not directly contribute to transposon silencing. Only cleavage in the presence of empty Siwi protein will be beneficial to transposon silencing. Therefore, making BmAgo3 cleavage dependent on BmGtsf1L and confining this to the BmVreteno environment would represent an effective way of optimizing BmAgo3 cleavage effectivity.

While it has been revealed that BmVreteno can establish an environment where piRNA-loaded BmAgo3 and empty Siwi are brought together, it is not fully understood how empty Siwi is provided (Nishida *et al*, 2020). Using our BmGtsf1L stable cell line, we did detect an interaction between BmGtsf1L and Siwi, but clearly much weaker than the BmAgo3-BmGtsf1 interaction. Our small RNA sequencing results further suggests that BmGtsf1L interacts with unloaded Siwi. Taken together, we hypothesize that Siwi is retrieved as a tertiary interaction via BmVreteno. It is possible that, in analogy to *Drosophila* Krimper, other eTudor domains of BmVreteno may bind unloaded Siwi (Sato *et al*, 2015; Webster *et al*, 2015). Using AlphaFold modeling, we uncovered in total three eTudor domains in BmVreteno. Two of these do not have an intact aromatic cage, suggesting they may bind empty, unmethylated Siwi.

We and others have shown that BmVreteno is spliced into two isoforms: Long (L) and Short (S). In addition, we know that these two isoforms form heterodimers. *In vitro* RNA cross-linking experiments have revealed that the RRM domain contained within BmVreteno-L binds RNA (Nishida *et al*, 2020). Therefore, it is tempting to speculate that the BmVreteno heterodimer can bring in one target RNA molecule, which is then processed by BmAgo3 and whose 3′-end cleavage product is subsequently loaded onto empty Siwi. Nonetheless, a single BmVreteno-L molecule could in principle also recruit both BmAgo3, Siwi and at least one more protein, so the question of why BmVreteno heterodimerizes remains unanswered.

BmVreteno homologs that are expressed within the germ cells of flies (Vreteno), fish, and mice (Tdrd1) are all required for piRNA biogenesis but have different domain organizations (Reuter *et al*, 2009; Vagin *et al*, 2009; Handler *et al*, 2011; Huang *et al*, 2011; Zamparini *et al*, 2011). *Drosophila* and silkworm Vreteno contain an RRM domain and a MYND domain followed by two or three eTudor domains, respectively. Notably, the expression of two Vreteno isoforms seems to be restricted to silkworm. However, in addition to Vreteno, flies also express a Vreteno-like protein in their ovaries, which is called Veneno (Brosh *et al*, 2022). DmVeneno has a very similar domain architecture compared to DmVreteno but is lacking an N-terminal RRM and, as such, mimics the domain organization of the BmVreteno-S isoform (Nishida *et al*, 2020; Brosh *et al*, 2022). Vreteno and Veneno orthologs can also be found in the mosquito species *Aedes aegypti*. Here, Veneno acts as an adaptor protein that brings the ping-pong partners Piwi5 and Ago3 in close proximity for viral piRNA biogenesis (Joosten *et al*, 2019). It would be interesting to study whether Veneno and Vreteno can dimerize and if they colocalize within the nuage of flies and mosquitoes. The mouse and fish Vreteno homologs (Tdrd1) also lack an RRM domain but contain four eTudor domains instead, raising the question how target RNA is provided within these complexes to facilitate *de novo* piRISC assembly. Multivalent interactions within the nuage that are (in part) established by Tudor domains may play an important role here.

The above examples illustrate that many nuage-residing proteins contain multiple eTudor domains, which contribute to the assembly of this phase-separated structure through the formation of multivalent interactions (Chen *et al*, 2011). Importantly, the depletion of a single nuage component can affect nuage integrity and concurs with a significant reduction in piRNA levels. Interestingly, however, in

 © 2023 The Authors

C. elegans most eTudor-domain-containing proteins that reside in germ granules only harbor one eTudor domain. So how can a single eTudor domain establish a binding platform to recruit multiple proteins? In this study we reveal that a single eTudor domain (AF-eTD1) of BmVreteno can do so by establishing dual binding interfaces. The aromatic cage facilitates the binding of piRNA-loaded, methylated BmAgo3, whereas the hydrophobic pocket allows for binding of BmGtsf1L. The BmGtsf1L C-terminal residues (W99, D100) that are indispensable for binding to the BmVreteno hydrophobic pocket are broadly conserved. This indicates that Gtsf proteins might have a preserved mode of binding, which corresponds to a novel type of domain-linear motif interaction. However, more structural studies are needed to understand to which extent the hydrophobic pocket is conserved among other eTudor domains. Interestingly, we recently uncovered another, novel binding interface on an eTudor domain of the *C. elegans* protein TOFU-6 (Podvalnaya *et al*, 2023). This implicates that eTudor domains are much more versatile in establishing multivalent interactions than previously anticipated.

In this study, we developed a successful strategy based on AlphaFold for the prediction of protein interaction interfaces involving linear motifs. We note that interface predictions by AlphaFold always return the two protein fragments in contact with each other, making it most of the time very difficult to distinguish good from bad structural models simply by visual inspection. Confidence in reported structural models can be gained from metrics such as the model confidence that is computed by AlphaFold and, as we showed, the recurrent observation of residues predicted in contact with each other when alternating the length of protein fragments submitted for interface prediction. Our work also suggests that interface predictions with AlphaFold using full-length proteins might be unsuccessful but more systematic studies are needed to confirm this. Our results further indicate that AlphaFold is able to extrapolate from the training set of protein structures within the PDB to accurately predict protein interaction interfaces it has never seen before. Physics-based models such as molecular dynamics simulations as we employed here also offer a route to investigate and critically assess the importance of binding interfaces predicted by AlphaFold (preprint: Zhang *et al*, 2023).

A recent study from Arif *et al* (2022) revealed that Gtsf proteins contribute to the piRNA-guided endonuclease activity of PIWI proteins *in vitro*. The authors proposed a model in which the binding of Gtsf would induce a conformational change in the piRISC-PIWI complex upon pairing with its RNA target. In addition, while our manuscript was in preparation, another paper reported that BmGtsf1L associates with BmAgo3 and modestly enhances its slicing activity, whereas BmGtsf1 specifically increases Siwi endonuclease activity (Izumi *et al*, 2022). Thus, the stimulation of catalytic activity of PIWI proteins seems to be a conserved function of Gtsf proteins. We note, however, that a molar excess of Gtsf was required to stimulate PIWI slicing activity (Arif *et al*, 2022) and the stimulatory effect of BmGtsf1L on BmAgo3 was relatively small (Izumi *et al*, 2022). We hypothesize that the catalytic effects of Gtsf proteins on PIWI proteins are generally enhanced by eTudor domains that interact with both the loaded PIWI protein and a Gtsf protein, thereby effectively restricting PIWI cleavage to those environments that contain the required eTudor domain. Indeed, in flies and mouse, conserved aromatic residues within the C-terminus of Gtsf1, that we show to interact with an eTudor domain, contribute to PIWI binding (Dönertas *et al*, 2013; Ohtani *et al*, 2013; Yoshimura *et al*, 2018) and PIWI target cleavage kinetics (Arif *et al*, 2022). It is possible that these reported interactions are in fact mediated via, or at least stimulated by eTudor domains. To firmly test our hypothesis, piRISC kinetics will need to be studied in settings that recapitulate the interactions between PIWI, Gtsf and eTudor domains, using recombinant proteins (Dönertas *et al*, 2013; Ohtani *et al*, 2013; Yoshimura *et al*, 2018; Arif *et al*, 2022).

To conclude, our studies start to address the question of why PIWI proteins evolved the requirement of a co-factor for target cleavage. Given that other Argonaute proteins can efficiently cleave target RNA without such co-factors, it seems reasonable to pose that the Gtsf dependence of PIWI proteins serves a purpose. We propose that Gtsf proteins are required to dictate where and possibly when target RNAs are cleaved by PIWI proteins to allow for piRNA amplification. Interestingly, in flies and mouse Gtsf proteins also contribute to PIWI-induced transcriptional gene silencing. However, the exact role of Gtsf1 in this process, which does not involve target RNA cleavage, still remains elusive (De Fazio *et al*, 2011; Dönertas *et al*, 2013; Ohtani *et al*, 2013; Yoshimura *et al*, 2018). Perhaps Gtsf proteins restrict conformational changes of PIWI proteins upon target recognition to loci of strong homology, preventing the establishment of transcriptional silencing at erroneous loci. Further studies will be required to test these ideas.

Materials and Methods

BmN4 cell culture and transfection

BmN4 cells were cultured at 27°C in IPL-41 insect medium (Gibco) supplemented with 10% FBS (Gibco) and 0.5% Pen-Strep (Gibco). Twenty-four hours prior to transfection, ~4×10^6 cells were seeded in a 10-cm dish (using one 10-cm dish for each condition). Cells were transfected with plasmid DNA using X-tremeGene HP (Roche) transfection reagent, according to the manufacturer's instructions. Seventy-two hours post transfection cells were harvested, washed once in 5 ml ice-cold PBS and once more in 1 ml ice-cold PBS. Subsequently, cells were pelleted by centrifugation for 5 min at 500 *g* at 4°C and frozen at −80°C. The BmN4 cell line was obtained from R. Pillai. BmN4 cells were obtained from T. Kusakabe. Further details are available online (https://www.cellosaurus.org/CVCL_Z634). It was not authenticated and was not tested for mycoplasma.

RNAi in BmN4 cells

For preparation of dsRNA, template DNAs were prepared by PCR using primers that contained flanking T7 promoter sequences. Primers for preparation of dsRNA can be found in Supplementary Materials (Dataset EV3). dsRNA was generated by *in vitro* transcription using the HiScribe T7 kit (NEB), according to the manufacturer's instructions. Transcribed RNA was purified by phenol/chloroform extraction, precipitated with ethanol, and annealed in water. For dsRNA-mediated gene knockdown, ~2×10^6 BmN4 cells were transfected with 10 μg of dsRNA using X-tremeGene HP. Seventy-two hours after transfection, cells were again transfected with dsRNA and the dsRNA-treatment was repeatedly performed

every 3 days for at least three times for BmGtsf1L depletion and four times for BmVreteno or BmAgo3 knockdown.

Generation of stable cell lines

For the generation of 3xFLAG-eGFP, 3xFLAG-BmAgo3, and 3xFLAG-Siwi stable cell lines, ~4 × 10^6 BmN4 cells were seeded in a 10-cm dish. Cells were transfected with 10 µg of plasmid DNA (Dataset EV3) and cultured under Puromycin (Gibco) selection (5 µg/ml) for at least 4 additional weeks. Stable integration of plasmid DNA was verified by Western blot. The HA-BmGtsf1L-eGFP stable cell line was generated in a similar manner. All stable cell lines are polyclonal.

Plasmid construction

For expression of plasmids in BmN4 cells, all genes were PCR amplified using BmN4 cDNA and then cloned into the pBEMBL vector (kind git of Ramesh Pillai), which harbors an OpIE2 promoter and an OpIE2 polyA tail (Xiol *et al*, 2012). The plasmids that were used to generate stable cell lines additionally contain a puromycin cassette, where the BmA3 promoter drives the expression of the puromycin-N-acetyltransferase (*pac*) gene, followed by the OpIE2 polyA sequence.

For recombinant protein expression in *E. coli*, coding sequences were cloned into the pET28a(plus) vector that contains an N-terminal $(HIS)_6$-tag or into the pGEX-6p vector for GST-tagged protein expression (kind gift from H. Ullrich lab). All primers, vector backbones, and detailed cloning strategies can be found in Dataset EV3.

Immunoprecipitations

Directly before use, BmN4 cell pellets were thawed on ice and lysed in 1 ml IP-150 Lysis Buffer (30 mM Hepes [pH7.4], 150 mM KOAc, 2 mM $Mg(OAc)_{2,}$ and 0.1% Igepal freshly supplemented with EDTA-free protease inhibitor cocktail and 5 mM DTT) for 1 h by end-over-end rotation at 4°C. Cells were further lysed by passing the lysate 10 times through a 20-gauge syringe needle followed by five passes through a 30-gauge needle. Cell debris was pelleted by centrifugation at 17,000 *g* for 20 min at 4°C. Supernatant fractions were collected and subjected to immunoprecipitations. In case of RNase treatment, 20 µl of RNaseA/T1 (Thermo Scientific, #EN0551) were added to 1 ml of IP-150 lysis buffer prior to lysis (according to the manufacturer's instructions).

Immunoprecipitations were performed using Pierce™ Anti-HA Magnetic Beads (30 µl bead suspension per reaction, Thermo Fisher, #88836), Anti-FLAG M2 Magnetic Beads (20 µl bead suspension per reaction, Sigma, #M8823) or GFP/RFP-Trap Magnetic Agarose beads (15 µl bead suspension per reaction, Chromotek). When using endogenous antibodies, 3 µg of affinity-purified antibodies were coupled to 15 µl of pre-equilibrated Protein G Dynabeads (30 µl bead suspension, Invitrogen) for one reaction. Normal rabbit IgG (Cell Signaling, #2729) or mouse non-immune serum (n.i.) served as controls. Beads and antibodies were incubated for 1 h at 4°C by end-over-end rotation in 500 µl of IP-150 Lysis Buffer. Bead-conjugated antibodies were then washed three times in 1 ml of IP-150 Lysis Buffer. Equilibrated beads were subsequently incubated with the BmN4 cell lysate and incubated overnight by end-over-end rotation at 4°C. The next day, immunoprecipitated complexes were washed five times using 1 ml of IP-150 Lysis Buffer and were subsequently used for immunodetection using Western Blot analysis.

Western blot

Samples were prepared in 1× Novex NuPage LDS sample buffer (Invitrogen) supplemented with 100 mM DTT and were heated at 95°C for 10 min prior to resolving on a 4–12% Bis-Tris NuPage NOVEX gradient gel (Invitrogen) in 1× Novex NuPAGE MOPS SDS Running Buffer (Invitrogen) at 140 V. For the detection of endogenous BmGtsf1L, proteins were resolved on a 15% Bis-Tris polyacrylamide gel. Separated proteins were transferred to a nitrocellulose membrane (Amersham) overnight at 20 V using 1× NuPAGE Transfer Buffer (Invitrogen) supplemented with 10% methanol. The next day, the membrane was blocked for 1 h in 1× PBS-Tween (0.05%) supplemented with 5% skim milk and incubated for 1 h with primary antibodies diluted in blocking buffer (1:1,000 anti-Flag; 1:1,000 anti-GFP; 1:1,000 anti-HA; 1:1,000 anti-actin, 1:2,500 anti-tubulin; 1:1,000 for all endogenous antibodies). Subsequently, the membrane was washed three times for 5 min in PBS-Tween, prior to 1 h incubation with the secondary antibody, using 1:10,000 IRDye 800CW Goat anti-mouse and IRDye 680LT Donkey anti-rabbit IgG (LI-COR) and imaged on an Odyssey CLx imaging system (LI-COR). Secondary antibodies used for chemiluminescence-based detection were 1:1,000 rat monoclonal anti-mouse Ig HRP (Clone eB144, Mouse TrueBlot ULTRA, Rockland #18-8817-30), 1:10,000 goat anti-rabbit IgG HRP-linked antibody (Cell Signaling Technology, #7074), and 1:10,000 horse anti-mouse IgG, HRP (Cell Signaling Technology, #7076). Chemiluminescence signals were detected using ECL select Western Blotting detection reagent (Cytvia, #GERPN2235) and imaged on a Fusion FX imaging system (Vilber).

Recombinant protein purification

GST alone as well as GST-3C-BmVreteno variants and fragments were expressed from pGEX6p vectors. His6-thrombin-BmGtsf1L variants were expressed in pET vectors. Transformed plasmids were expressed in *E. coli* (BL21 DE3 codon+, Agilent) overnight at 18°C using 0.5 mM IPTG in LB media. Cells were lysed in ice-cold lysis buffer (30 mM Tris-Cl pH 8.0, 500 mM NaCl, 0.5 mM TCEP, 5% glycerol, an EDTA-free complete protease inhibitor cocktail, and an additional 10 mM imidazole pH 8.0 for BmGtsf1L purifications), using a CF1 continuous flow cell disruptor from constant systems at 29 kpsi and cleared by centrifugation at 40,000 *g* for 30 min at 4°C. Recombinant proteins were affinity-purified from cleared lysates using a NGC Quest Plus FPLC system (Biorad) and GSTrap HP (GST-tagged BmVreteno and GST), or HisTrap HP (His6-tagged BmGtsf1L) 5 ml columns (Cytiva), according to the manufacturers protocols. Eluted proteins were concentrated using Amicon spin concentrators (Merck Millipore) and subjected to gel filtration (Superdex 75 and 200 16/60 pg, Cytiva, in 25 mM Na-Hepes, 300 mM NaCl, 10% Glycerol, pH 7.4).

To obtain an untagged BmVreteno (181-386) antigen fragment for immunization, the GST-tagged fragment from the affinity step was digested with 3C protease (1:100 w/w) overnight at 4°C during dialysis (30 mM Tris-Cl pH 8.0, 500 mM NaCl, 1 mM DTT, 5%

 © 2023 The Authors

glycerol). The digested protein was re-applied to a GSTrap HP 5 ml column to absorb the free GST. The flow through from this step, containing the untagged BmVreteno (181-386) antigen was concentrated using Amicon spin concentrators and subjected to gel filtration (Superdex 75 16/60 pg in PBS). Another round of free GST absorption via GSTrap, followed by gel filtration (Superdex 75 16/60 pg in PBS) was performed to remove residual free GST from the untagged BmVreteno (181-386) antigen.

For all recombinant proteins, peak fractions after the final gel filtration were pooled and protein concentration was determined by using absorbance spectroscopy and the respective extinction coefficient at 280 nm, before aliquots were flash frozen in liquid nitrogen and stored at −80°C.

GSH pull-downs

Glutathione Sepharose 4B beads (Cityva) were equilibrated (20 µl beads suspension for each reaction) by three washes in PBS containing 0.1% Triton-X100 (PBS-T), and the resin was pelleted by mild centrifugation at 1,000 *g* for 2 min at 4°C. Next, 5 µM of GST-BmVreteno was added to the beads together with 10 µM of His-BmGtsf1L, and samples were incubated for 2 h by end-over-end rotation at 4°C. Beads were pelleted by centrifugation at 1,000 *g* for 2 min at 4°C and washed three times with PBS-T. Finally, pelleted beads were resuspended in 25 µl 1× Novex NuPage LDS sample buffer (Invitrogen) supplemented with 100 mM DTT and were heated at 95°C for 5 min prior to resolving (40% of the sample) on a 4–12% Bis-Tris NuPage NOVEX gradient gel (Invitrogen) in 1× Novex NuPAGE MES SDS Running Buffer (Invitrogen) at 180 V. Proteins on the gel were visualized by staining with InstantBlue Coomassie protein stain (Abcam).

Antibodies

Monoclonal antibodies for detection and/or immunoprecipitation of endogenous Siwi, BmAgo3, BmSpn-E, and BmQin were a kind gift from Mikiko Siomi (Nishida *et al*, 2015). Rabbit polyclonal antibodies for BmAgo3 detection were provided by Ramesh Pillai (Xiol *et al*, 2012).

The monoclonal anti-BmGtsf1L antibody was generated in the Siomi lab by immunizing mice with purified GST-tagged full-length BmGtsf1L. Fusing myeloma generated hybridomas as described previously (Nishida *et al*, 2015).

The rabbit polyclonal anti-BmVreteno antibody was generated by immunizing rabbits with the affinity-purified BmVreteno (186–381) antigen (Eurogentec). Two milliliters of sulfolink resin (Thermo Fisher Scientific) were covalently conjugated with 3 mg of GST-tagged BmVreteno (181-386) according to the manufacturer's protocol. Ten milliliters of final bleed of each rabbit serum were incubated with 1 ml of GST-BmVreteno (181-386)-conjugated sulfolink resin at 4°C overnight while rotating. After incubation, the resin was washed with PBS containing 0.1% Triton X-100, followed by a wash with PBS in a gravity-flow poly-prep column (Biorad). Elution of polyclonal antibody species was performed using low pH (100 mM Glycine-Cl, 150 mM NaCl, pH 2.3), followed by immediate neutralization of elution fractions with Tris-Cl pH 8.0. The eluted antibodies were re-buffered using a PD-10 column (PBS, 10% glycerol, 0.05% NaN_3) and concentrated to 1 mg/ml using Amicon spin concentrators before flash freezing in liquid nitrogen and storage at −80°C.

Monoclonal anti-HA was produced in house (clone 12CA5, Core Facility Protein Production). Rabbit polyclonal Anti-HA (Sigma-Aldrich, #SAB4300603), mouse monoclonal anti-Flag M2 (Sigma-Aldrich, #F3165), rabbit polyclonal anti-FLAG (Milipore, #F7425), rabbit polyclonal anti-actin (Sigma-Aldrich, #A5060), mouse monoclonal anti-alpha Tubulin (clone B-5-1-2, Sigma-Aldrich, #T6074), rabbit polyclonal anti-GFP (Origene, #TP401), and mouse monoclonal anti-GFP (clone B-2, Santa Cruz, #sc-9996) are all commercially available.

Sequence alignment

Clustal W (Larkin *et al*, 2007) and Jalview software (Waterhouse *et al*, 2009) was used for protein alignment and visualization.

Microscopy

For co-localization studies, approximately 2 × 10^4 cells were seeded per well in an 8-well µ-slide (Ibidi, #80826). The next day, cells were transfected with 100 ng of each corresponding plasmid using X-tremeGene HP. Twenty-four hours post transfection, live cells were fixed and imaged. Confocal imaging (Figs 1K and EV2A) was performed using a STELLARIS 8 FALCON microscope (Leica Microsystems, Mannheim, Germany) equipped with a White Light Laser (WLL). Images (512 × 512 pixel format, pixel size 180 nm) were acquired with a 63×/1.40NA oil immersion objective, using: Channel 0: 488 nm excitation line and the emission band ranging from 500 to 540 nm using a detector HyD X2; Channel 1: 548 nm excitation line and the emission band ranging from 560 to 590 nm using a detector HyD X4; Channel 2: 600 nm excitation line and the emission band ranging from 620 to 750 nm using a detector HyD R5. A sequential scan was performed line by line, and accumulation mode was set to 300 to have enough photon counts for fluorescence lifetime imaginging.

The Leica TCS SP5 with a 60× oil immersion objective lens was used for images in Figs 3D and E, and EV4D.

All images were processed using FIJI (Schindelin *et al*, 2012) and Adobe Illustrator software.

RNA isolation and small RNA sequencing

Per condition, one well of a 6-well plate was seeded with 6 × 10^5 BmN4 cells 24 h prior to transfection with either HA-eGFP, HA-BmAgo3, HA-Siwi, or HA-BmGtsf1L using X-tremeGene HP transfection reagent. Seventy-two hours post transfection, cells were harvested, and an anti-HA immunoprecipitation was performed as described above. The experiment was performed in duplicate. Anti-BmAgo3 and anti-Siwi IPs were performed in IP-500 (30 mM Hepes [pH 7.4], 500 mM KOAc, 2 mM $Mg(OAc)_2$, and 0.1% Igepal, freshly supplemented with an EDTA-free protease inhibitor cocktail and 5 mM DTT). Immunopurified RNAs were extracted from beads by adding 1 ml of Trizol LS (Invitrogen #10296028), according to the manufacturer's instructions. The lysate was incubated at RT for 5 min to allow complete dissociation of the nucleoprotein complex. Next, 200 µl of chloroform was added to 1 ml of lysate, followed by harsh mixing and centrifugation at 12,000 *g* for 15 min at 4°C.

© 2023 The Authors

Another round of chloroform extraction was performed, and the aqueous phase was transferred to a fresh tube to which 1 volume (500 μl) of ice-cold isopropanol was added for RNA precipitation. RNA pellets were washed twice in 1 ml of 70% ice-cold ethanol and centrifuged at 7,500 *g* for 10 min at 4°C. The RNA pellet was air-dried and dissolved in nuclease-free water.

NGS library prep was performed with NEXTflex Small RNA-Seq Kit V3 following Step A to Step G of Bioo Scientific's standard protocol (V16.06) using the NEXTFlex 3′ SR Adaptor and 5′ SR Adaptor (5′ rApp/NNNNTGGAATTCTCGGGTGCCAAGG/3ddC/ and 5′ GUUCAGAGUUCUACAGUCCGACGAUCNNNN, respectively). Libraries were prepared with a starting amount of 7 ng and amplified in 25 PCR cycles.

Amplified libraries were purified by running an 8% TBE gel and size-selected for 15–35 nt.

Libraries were profiled on a high-sensitivity DNA chip on a 2100 Bioanalyzer (Agilent Technologies) and quantified using the Qubit dsDNA HS Assay Kit, in a Qubit 2.0 Fluorometer (Life Technologies).

All samples were pooled in an equimolar ratio and sequenced on 1 Highoutput NextSeq 500/550 Flowcell, SR for 1× 84 cycles plus 7 cycles for the index read.

Bioinformatic analyses

The quality of raw sequenced reads was accessed with FastQC, Illumina adapters were then removed with cutadapt (-O 5 -m 28 -M 45), reads with low-quality calls were filtered out with fastq quality_filter (-q 20 -p 100 -Q 33). Using information from unique molecule identifiers (UMIs) added during library preparation, reads with the same sequence (including UMIs) were collapsed to remove putative PCR duplicates using a custom script. Prior to mapping, UMIs were trimmed (seqtk trimfq -b 4 -e 4) and library quality re-assessed with FastQC. Reads were aligned against the silkworm (*Bombyx mori*) genome assembly obtained from lepbase GCA_000151625.1 with bowtie v1.1.1 (-l 40 -n 2 -e 70 -m 1 –tryhard –best –strata –chunkmbs 256 –phred33-quals). The locations of repeat elements were also downloaded from lepbase, repeat masker scaffolds (ASM15162v1), converted to genomic location with rmsk2bed. These locations were used to select reads mapping to repeats by intersecting with bedtools intersect (-wa -wb -bed -f 1.0 -nonamecheck) with either the flags -s or -S to determine which small RNAs map sense or antisense, respectively, to the annotated repeats. After filtering, length profiles were obtained by summarizing the length of these reads. Sense/antisense bias was determined by calculating the ratio of reads mapping in the same or the opposite strand for each annotated repeat - repeats with 10 or fewer mapped reads were excluded. Nucleotide bias of piRNAs was determined by summarizing the number of times a base is present in any given piRNA (read sequence) position.

Mass-spectrometry

About 4×10^6 BmN4 cells were transfected with HA-tagged BmGtsf1L or with HA-eGFP, which served as a control to detect nonspecific binders. Cells were harvested 72 h post transfection and an anti-HA immunoprecipitation was performed (as described above) on 4 mg total protein lysate. The experiment was performed using two technical duplicates to perform quantitative mass-spectrometry based detection of unique peptides using stable dimethyl isotope labeling (Hsu *et al*, 2003).

Protein in-gel digestion

Proteins were separated briefly in a 10% NuPAGE Bis-Tris gel, stained with Coomassie blue, and cut into small gel cubes, followed by destaining in 50% ethanol/25 mM ammonium bicarbonate. Afterwards, proteins were reduced in 10 mM DTT at 56°C and alkylated by 50 mM iodoacetamide in the dark at room temperature. Enzymatic digestion of proteins was performed using trypsin (1 μg per sample) in 50 mM TEAB (triethylammonium bicarbonate) overnight at 37°C. Following peptide extraction sequentially using 30 and 100% acetonitrile, the sample volume was reduced in a centrifugal evaporator to remove residual acetonitrile. The sample volume was filled up to 100 μl by adding 100 mM TEAB.

Dimethyl-labeling

Dimethyl-labeling was performed as previously reported (Boersema *et al*, 2009). Briefly, the digested samples were labeled as "Light" or "Heavy" by adding formaldehyde or formaldehyde-d_2, respectively. This was followed by addition of $NaBH_3CN$. Thereafter, the samples were incubated at room temperature with orbital shaking for 1 h. The labeling reaction was quenched by adding ammonia solution. Next, peptides were acidified with formic acid to reach pH ~3. The paired-labeled samples were then combined. The resultant peptide solution was purified by solid-phase extraction in C_{18} StageTips (Rappsilber *et al*, 2003).

Liquid chromatography tandem mass spectrometry

Peptides were separated in an in-house packed 30-cm analytical column (inner diameter: 75 μm; ReproSil-Pur 120 C_{18}-AQ 1.9-μm beads, Dr. Maisch GmbH; heated at 40°C) by online reverse phase chromatography through a 105-min nonlinear gradient of 1.6–32% acetonitrile with 0.1% formic acid at a nanoflow rate of 225 nl/min. The eluted peptides were sprayed directly by electrospray ionization into a Q Exactive Plus Orbitrap mass spectrometer (Thermo Scientific). Mass spectrometry measurement was conducted in data-dependent acquisition mode using a top10 method with one full scan (mass range: 300–1,650 *m/z*; resolution: 70,000, target value: 3×10^6, maximum injection time: 20 ms) followed by 10 fragmentation scans via higher energy collision dissociation (HCD; normalized collision energy: 25%, resolution: 17,500, target value: 1×10^5, maximum injection time: 120 ms, isolation window: 1.8 *m/z*). Precursor ions of the unassigned or +1 charge state were rejected. Additionally, precursor ions already isolated for fragmentation were dynamically excluded for 20 s.

Mass spectrometry data processing and statistical analysis

Raw data files were processed by the MaxQuant software package (version 1.5.2.8) (Cox & Mann, 2008) using its built-in Andromeda search engine (Cox *et al*, 2011) and default settings. Spectral data were searched against a target-decoy database consisting of the forward and reverse sequences of the bait proteins (HA-eGFP and HA-BmGtsf1L), *Bombyx mori* proteomes (UniProt 18,382 entries; NCBI 29,282 entries) downloaded on 8th January 2018, a collection of self-cloned *Bombyx mori* genes (28 entries), and a list of 245 common contaminants. Corresponding labels were selected for "Light" (DimethLys0 and DimethNter0) and "Heavy" (DimethLys4 and

 © 2023 The Authors

DimethNter4) labels. A maximum of 3 labeled amino acids per peptide were considered. Trypsin/P specificity was assigned. Carbamidomethylation of cysteine was set as a fixed modification. Oxidation of methionine and acetylation of the protein N-terminus were chosen as variable modifications. A maximum of 2 missed cleavages were tolerated. The minimum peptide length was set to be 7 amino acids. The false discovery rate (FDR) was set to 1% for both peptide and protein identifications.

For protein quantification, minimum ratio count of two was required. Both the unique and razor peptides were used for quantification. The "re-quantify" function was switched on. The "advanced ratio estimation" option was also chosen. Downstream data analysis was performed in R statistical environment. Reverse hits, potential contaminants and protein groups "only identified by site" were filtered out. Protein groups with at least two peptides including at least one unique peptide were retained.

AlphaFold predictions

We used the following sequences for AlphaFold predictions:

BmGtsf1L:

MDDPFVSCPYNPIHRVPRSRLQRHIVKCEWINPTMIACPYNATHRYTQED

MKFHVLNCPSKTSIFPIEKPPKTVASITTPKIILQKEYLPETDPNHEIWDD

BmVreteno:

MSNHSRPQRRREWDPMRDDFNEHTYDVQYADDNAGEQVQLDHTKLYIINI

PRGLSEDGIRAAFSKHGKVLSARLSKNPNKRFAIVQFETASEAKLAMMKM

NGSEPLNLKISIAHKTIRKTQHDNKDRNYSTSRNGHCSRDEASSISSKGW

NMRNLDDVMNNDEIDEIDDMIHEDHDDNLDLELDMLTLKQLKIKEEQLMC

KRRLLLRHAEKRQVAPHSSAGRSVLPDGRIVVRNNANETDSAEVEPSFAG

AGSESLKTPGLERNASRQCVKCGAPADWYCSRCAITPYCSQTCQTRDWTE

RHKSVCHYLAPLKTAGGFEAEATSSKSVSNSTPMRSSHSPPTKQQRGEAD

ETDNKAKNIQEPRQNYHRPSNSGPNKNIPGKNQDPRRPATSREAIEEETE

ERGARNPKPAEATKDKHHPMNPVTFQRRQLKSNPVVDAQPAPREQQQPAA

TRAPEASPTEQRESTRRTLVPDRCLIDSLSEGDVVLVSVELKASECCTKQ

GGYVCLSMHEKYESDYQKLCEDYVLDCEADSDEYKIITGDTFSYLSPEDG

GWYRARALNTTMAALLDGSKVVYLRMNDKVKKLPAKYSGIPEFCCVLNAD

VEVGLNLKCSLLSKTPNGFKVTLENVETEANVGEGEITRWIPEVDYPPPV

KNVPVQRSVEIPEVPRPEIKNKSRVILVDATDVQRVFVRPADTRSQKAFD

NILQDVLLYGTTAEPLKEPPSKGQTVVSKYTDNLHYRALCKRTSVNKNKY

LLEYIEYGNIEITQLNRLYPCPEHLSVTSLASLTSHVQLDTTVGELTPRA

LEYIETIKEEEMILTLSSGGDTAQSGAALVNLTLVKNNDNVNKRIEELCT

PEWKKLELKGVDVIETERLMYGTALDYIELPAAPFDLQVLDEVGLDSGNI

SGCPTNSDYVRYVMTKLPARMREYCESEFGRQPYLPAAEELCIAQLPPSS

EWHRAVVLEQILGPGGGTARVLFVDHGNVAEVPVSSLRKMLAEFVTDLPA

VACQIVIEDFPKQATAEMLAKARRFMSGPDKARAAQLPVRGCDKQDVGIY

AIRVPELLEAMTE

We ran AlphaFold v2.2 (Jumper *et al*, 2021) for all monomeric protein predictions and AlphaFold-Multimer v2.2 (preprint: Evans *et al*, 2022) for all protein complex predictions with the following parameters:

--max_template_date=2020-05-14

--db_preset=full_dbs

--use_gpu_relax=False

For every AlphaFold run, 5 models were predicted with one seed per model by setting the following parameter:

--num_multimer_predictions_per_model=1

Out of the five models generated, we used only the model ranked_0 for further processing and interpretation. We defined two residues, one from each protein fragment, to be in contact with each other in predicted AlphaFold models if at least one heavy atom from one residue is less than 5 Å away from any heavy atom from the other residue. Distance measurements between heavy atoms were obtained using the function cmd.distance from PyMOL. The model confidence was extracted from the ranking_debug json file. The PAE matrix was extracted from the pickle file of the model. We used the software PyMOL (TM) Molecular Graphics System, Version 2.5.0. Copyright (c) Schrodinger, LLC., for the visualization and superimposition of AlphaFold models. The superimposition of the structural model involving AF-eTD1 and the last 5 residues of BmGtsf1L with the solved structure 3NTH was done using the cealign command, where AF-eTD1 was set as the mobile entity and the chain A of 3NTH as the target entity for superimposition. For the superimposition of AlphaFold-predicted BmGtsf1L with the solved structure 6X46, we extracted the two Zn fingers from AlphaFold-predicted BmGtsf1L (residue 8–34 and 35–64 for the two Zn fingers, respectively) and aligned them to the two Zn fingers from chain A of 6X46 (residue 14–41 and 48–75 for the two Zn fingers, respectively). The align command was used for the superimposition where AlphaFold-predicted BmGtsf1L Zn fingers were set as the mobile entities and the Zn fingers from the first ensemble state of 6X46 as the target entities.

IUPred predictions were obtained by submitting full-length sequences to the webserver of IUPred2A (Mészáros *et al*, 2018) and selecting the option IUPred2 long disorder (default) for disorder propensity predictions.

We used the Python libraries, pandas (McKinney, 2010) for data analysis, and Matplotlib (Hunter, 2007) and seaborn (Waskom, 2021) for data visualization.

Molecular dynamics simulations

We ran atomistic molecular dynamics simulations using the AlphaFold structural model involving AF-eTD1 of BmVreteno and the 10

© 2023 The Authors

last residues of BmGtsf1L. We used the Amber99SB*-ILDN-q protein force field (Hornak *et al*, 2006; Best & Hummer, 2009; Lindorff-Larsen *et al*, 2010; Best *et al*, 2012) and the TIP4P-D water model (Piana *et al*, 2015). Molecular dynamics simulations were run in GROMACS 2021 (www.gromacs.org) (Abraham *et al*, 2015).

The protein-peptide complex was simulated in a rhombic dodecahedron, with a minimum distance of 12 Å between protein atoms and box edges. One hundred and fifty millimolar NaCl were added to the solvated simulation system. The system was energy minimized and equilibrated for 1 ns in using the Berendsen thermostat and barostat at 300 K and 1 bar (Berendsen *et al*, 1984).

We run 10 independent simulations starting from the equilibrated starting structure, each with a different set of initial velocities. Each of the 10 simulations was run for 1 μs. The Bussi-Donadio-Parinello thermostat was used to maintain a simulation temperature of 300 K (Bussi *et al*, 2007). Parrinello-Rahman barostat was employed to keep pressure at 1 bar (Parrinello & Rahman, 1981). Electrostatics were described by the particle mesh Ewald method (PME). The cut-off for van der Waals interactions was 12 Å.

Simulations were analyzed with the MDAnalysis Python library (Michaud-Agrawal *et al*, 2011; Gowers *et al*, 2016). Two residues were deemed to be in contact if one pair of atoms was within 4.5 Å. The contacts maps from the 10 simulation runs, which were started from the same starting structured were averaged to produce a single contact map. Hydrogen bonds were quantified as described by Smith *et al* (2019).

Data availability

The datasets produced in this study are available in the following databases: (i) The accession number for the smRNA-seq data generated in this study is PRJNA940809 (https://www.ncbi.nlm.nih.gov/bioproject/PRJNA940809), (ii) Mass spectrometry proteomics data is available at https://massive.ucsd.edu under accession ID MSV000091404, (iii) All plasmids and reagents are available upon request.

Expanded View for this article is available online.

Acknowledgements

We thank all members of the Ketting Lab for fruitful discussions. We would like to thank Ramesh Pillai for providing plasmids, antibodies, and the BmN4 cell line. We are very grateful to the Siomi Lab for their generous supply of antibodies. The IMB Core Facility Microscopy (Sandra Ritz, Márton Gelléri, and Petri Turunen) is acknowledged for support with confocal microscopy. STELLARIS 8 Falcon Confocal was funded by Deutsche Forschungsgemeinschaft (DFG, German Research Foundation) project number 497669232. Support by the IMB Proteomics Core Facility is gratefully acknowledged (instrument funded by DFG INST 247/766-1 FUGG). We wish to thank Mario Dejung in particular, Proteomics Core Facility, for his continuous support and for extensive bioinformatic analysis. Support by the IMB Genomics Core Facility and the use of its NextSeq500 (funded by the DFG – INST 247/870-1 FUGG) are gratefully acknowledged. Support from IMB's IT department and especially help from Christian Diedrich for a local installation of AlphaFold is gratefully acknowledged. The GPU cluster on which all AlphaFold predictions were performed was funded by the Ministry of Science and Health (MWG), Rhineland Palatinate (funding id: TB-Nr.:3658/19). AWB was supported by the Peter und Traudl Engelhorn Foundation, by the Klaus Tschira Boost Fund and the German Scholars Organization (GSO/KT 14), as well as by an SPP-1935 start-up fund from the Deutsche Forschungsgemeinschaft (DFG, German Research Foundation). MC Siomi is supported by MEXT KAKENHI Grant Number JP19H05466. This project was funded by SFB1551 Project No. 464588647 of the DFG (Deutsche Forschungsgemeinschaft) for RFK, KL, and LS. This work was further supported by the DFG-funded project LU 2568/1-1 granted to KL. LSS acknowledges support from ReALity (Resilience, Adaptation, and Longevity), M^{3}ODEL, and the Forschungsinitiative des Landes Rheinland-Pfalz. Further, we gratefully acknowledge the advisory services offered and the computing time granted on the supercomputer Mogon II at Johannes Gutenberg University Mainz, which is a member of the AHRP (Alliance for High Performance Computing in Rhineland Palatinate) and the Gauss Alliance e.V. Open Access funding enabled and organized by Projekt DEAL.

Author contributions

Alfred W Bronkhorst: Conceptualization; data curation; supervision; funding acquisition; validation; investigation; visualization; writing – original draft; project administration; writing – review and editing. **Katja Luck:** Conceptualization; resources; software; supervision; funding acquisition; validation; visualization; writing – original draft. **Lukas Stelzl:** Data curation; software; formal analysis; validation; investigation; visualization; writing – original draft. **René F Ketting:** Conceptualization; resources; supervision; funding acquisition; validation; writing – original draft; writing – review and editing. **Shéraz Sadouki:** Investigation. **Martin M Möckel:** Investigation. **Sabine Ruegenberg:** Investigation. **Chop Y Lee:** Data curation; formal analysis; validation; investigation; visualization. **Antonio M de Jesus Domingues:** Data curation; formal analysis; visualization. **Tetsutaro Sumiyoshi:** Investigation; visualization. **Mikiko C Siomi:** Resources; supervision; validation. **Rossana Piccinno:** Data curation; validation; investigation; visualization.

Disclosure and competing interests statement

The authors declare that they have no conflict of interest.

References

Abraham MJ, Murtola T, Schulz R, Páll S, Smith JC, Hess B, Lindahl E (2015) GROMACS: high performance molecular simulations through multi-level parallelism from laptops to supercomputers. *SoftwareX* 1–2: 19–25

Akdel M, Pires DEV, Pardo EP, Jänes J, Zalevsky AO, Mészáros B, Bryant P, Good LL, Laskowski RA, Pozzati G *et al* (2022) A structural biology community assessment of AlphaFold2 applications. *Nat Struct Mol Biol* 29: 1056–1067

Arif A, Bailey S, Izumi N, Anzelon TA, Ozata DM, Andersson C, Gainetdinov I, MacRae IJ, Tomari Y, Zamore PD (2022) GTSF1 accelerates target RNA cleavage by PIWI-clade Argonaute proteins. *Nature* 608: 618–625

Berendsen HJC, Postma JPM, van Gunsteren WF, DiNola A, Haak JR (1984) Molecular dynamics with coupling to an external bath. *J Chem Phys* 81: 3684–3690

Best RB, Hummer G (2009) Optimized molecular dynamics force fields applied to the helix-coil transition of polypeptides. *J Phys Chem B* 113: 9004–9015

Best RB, de Sancho D, Mittal J (2012) Residue-specific α-helix propensities from molecular simulation. *Biophys J* 102: 1462–1467

Boersema PJ, Raijmakers R, Lemeer S, Mohammed S, Heck AJR (2009) Multiplex peptide stable isotope dimethyl labeling for quantitative proteomics. *Nat Protoc* 4: 484–494

 © 2023 The Authors

Brennecke J, Aravin AA, Stark A, Dus M, Kellis M, Sachidanandam R, Hannon GJ (2007) Discrete small RNA-generating loci as master regulators of transposon activity in *Drosophila*. *Cell* 128: 1089 – 1103

Brosh O, Fabian DK, Cogni R, Tolosana I, Day JP, Olivieri F, Merckx M, Akilli N, Szkuta P, Jiggins FM (2022) A novel transposable element-mediated mechanism causes antiviral resistance in *Drosophila* through truncating the Veneno protein. *Proc Natl Acad Sci USA* 119: e2122026119

Bussi G, Donadio D, Parrinello M (2007) Canonical sampling through velocity rescaling. *J Chem Phys* 126: 014101

Chen C, Nott TJ, Jin J, Pawson T (2011) Deciphering arginine methylation: Tudor tells the tale. *Nat Rev Mol Cell Biol* 12: 629 – 642

Chen K, Yu Y, Yang D, Yang X, Tang L, Liu Y, Luo X, Walters JR, Liu Z, Xu J *et al* (2020) Gtsf1 is essential for proper female sex determination and transposon silencing in the silkworm, *Bombyx mori*. *PLoS Genet* 16: e1009194

Cox J, Mann M (2008) MaxQuant enables high peptide identification rates, individualized p.p.b.-range mass accuracies and proteome-wide protein quantification. *Nat Biotechnol* 26: 1367 – 1372

Cox J, Neuhauser N, Michalski A, Scheltema RA, Olsen JV, Mann M (2011) Andromeda: a peptide search engine integrated into the MaxQuant environment. *J Proteome Res* 10: 1794 – 1805

Czech B, Munafò M, Ciabrelli F, Eastwood EL, Fabry MH, Kneuss E, Hannon GJ (2018) piRNA-guided genome defense: from biogenesis to silencing. *Annu Rev Genet* 52: 131 – 157

De Fazio S, Bartonicek N, Di Giacomo M, Abreu-Goodger C, Sankar A, Funaya C, Antony C, Moreira PN, Enright AJ, O'Carroll D (2011) The endonuclease activity of Mili fuels piRNA amplification that silences LINE1 elements. *Nature* 480: 259 – 263

Dönertas D, Sienski G, Brennecke J (2013) *Drosophila* Gtsf1 is an essential component of the Piwi-mediated transcriptional silencing complex. *Genes Dev* 27: 1693 – 1705

Evans R, O'Neill M, Pritzel A, Antropova N, Senior A, Green T, Žídek A, Bates R, Blackwell S, Yim J *et al* (2022) Protein complex prediction with AlphaFold-Multimer. *bioRxiv* https://doi.org/10.1101/2021.10.04.463034 [PREPRINT]

Friberg A, Corsini L, Mourão A, Sattler M (2009) Structure and ligand binding of the extended Tudor domain of D. melanogaster Tudor-SN. *J Mol Biol* 387: 921 – 934

Gainetdinov I, Colpan C, Arif A, Cecchini K, Zamore PD (2018) A single mechanism of biogenesis, initiated and directed by PIWI proteins, explains piRNA production in most animals. *Mol Cell* 71: 775 – 790

Ghildiyal M, Zamore PD (2009) Small silencing RNAs: an expanding universe. *Nat Rev Genet* 10: 94 – 108

Gowers R, Linke M, Barnoud J, Reddy T, Melo M, Seyler S, Domański J, Dotson D, Buchoux S, Kenney I *et al* (2016) MDAnalysis: a Python package for the rapid analysis of molecular dynamics simulations. pp 98–105

Gunawardane LS, Saito K, Nishida KM, Miyoshi K, Kawamura Y, Nagami T, Siomi H, Siomi MC (2007) A slicer-mediated mechanism for repeat-associated siRNA 5′ end formation in *Drosophila*. *Science* 315: 1587 – 1590

Han BW, Wang W, Li C, Weng Z, Zamore PD (2015) Noncoding RNA. piRNA-guided transposon cleavage initiates Zucchini-dependent, phased piRNA production. *Science* 348: 817 – 821

Handler D, Olivieri D, Novatchkova M, Gruber FS, Meixner K, Mechtler K, Stark A, Sachidanandam R, Brennecke J (2011) A systematic analysis of *Drosophila* TUDOR domain-containing proteins identifies Vreteno and the Tdrd12 family as essential primary piRNA pathway factors. *EMBO J* 30: 3977 – 3993

Hayashi R, Schnabl J, Handler D, Mohn F, Ameres SL, Brennecke J (2016) Genetic and mechanistic diversity of piRNA 3′-end formation. *Nature* 539: 588 – 592

Homolka D, Pandey RR, Goriaux C, Brasset E, Vaury C, Sachidanandam R, Fauvarque M-O, Pillai RS (2015) PIWI slicing and RNA elements in precursors instruct directional primary piRNA biogenesis. *Cell Rep* 12: 418 – 428

Hornak V, Abel R, Okur A, Strockbine B, Roitberg A, Simmerling C (2006) Comparison of multiple Amber force fields and development of improved protein backbone parameters. *Proteins* 65: 712 – 725

Horwich MD, Li C, Matranga C, Vagin V, Farley G, Wang P, Zamore PD (2007) The *Drosophila* RNA methyltransferase, DmHen1, modifies germline piRNAs and single-stranded siRNAs in RISC. *Curr Biol* 17: 1265 – 1272

Hsu J-L, Huang S-Y, Chow N-H, Chen S-H (2003) Stable-isotope dimethyl labeling for quantitative proteomics. *Anal Chem* 75: 6843 – 6852

Huang H-Y, Houwing S, Kaaij LJT, Meppelink A, Redl S, Gauci S, Vos H, Draper BW, Moens CB, Burgering BM *et al* (2011) Tdrd1 acts as a molecular scaffold for Piwi proteins and piRNA targets in zebrafish. *EMBO J* 30: 3298 – 3308

Huang X, Hu H, Webster A, Zou F, Du J, Patel DJ, Sachidanandam R, Toth KF, Aravin AA, Li S (2021) Binding of guide piRNA triggers methylation of the unstructured N-terminal region of Aub leading to assembly of the piRNA amplification complex. *Nat Commun* 12: 4061

Hunter JD (2007) Matplotlib: a 2D graphics environment. *Comput Sci Eng* 9: 90 – 95

Ipsaro JJ, Joshua-Tor L (2022) Developmental roles and molecular mechanisms of Asterix/GTSF1. *Wiley Interdiscip Rev RNA* 13: e1716

Ipsaro JJ, O'Brien PA, Bhattacharya S, Palmer AG, Joshua-Tor L (2021) Asterix/Gtsf1 links tRNAs and piRNA silencing of retrotransposons. *Cell Rep* 34: 108914

Izumi N, Shoji K, Sakaguchi Y, Honda S, Kirino Y, Suzuki T, Katsuma S, Tomari Y (2016) Identification and functional analysis of the pre-piRNA 3′ trimmer in silkworms. *Cell* 164: 962 – 973

Izumi N, Shoji K, Suzuki Y, Katsuma S, Tomari Y (2020) Zucchini consensus motifs determine the mechanism of pre-piRNA production. *Nature* 578: 311 – 316

Izumi N, Shoji K, Kiuchi T, Katsuma S, Tomari Y (2022) The two Gtsf paralogs in silkworms orthogonally activate their partner PIWI proteins for target cleavage. *RNA* 29: 18 – 29

Joosten J, Miesen P, Taşköprü E, Pennings B, Jansen PWTC, Huynen MA, Vermeulen M, Van Rij RP (2019) The Tudor protein Veneno assembles the ping-pong amplification complex that produces viral piRNAs in Aedes mosquitoes. *Nucleic Acids Res* 47: 2546 – 2559

Jumper J, Evans R, Pritzel A, Green T, Figurnov M, Ronneberger O, Tunyasuvunakool K, Bates R, Žídek A, Potapenko A *et al* (2021) Highly accurate protein structure prediction with AlphaFold. *Nature* 596: 583 – 589

Kawaoka S, Hayashi N, Suzuki Y, Abe H, Sugano S, Tomari Y, Shimada T, Katsuma S (2009) The Bombyx ovary-derived cell line endogenously expresses PIWI/PIWI-interacting RNA complexes. *RNA* 15: 1258 – 1264

Kawaoka S, Izumi N, Katsuma S, Tomari Y (2011) 3′ end formation of PIWI-interacting RNAs *in vitro*. *Mol Cell* 43: 1015 – 1022

Kirino Y, Vourekas A, Kim N, de Lima AF, Rappsilber J, Klein PS, Jongens TA, Mourelatos Z (2010) Arginine methylation of vasa protein is conserved across phyla. *J Biol Chem* 285: 8148 – 8154

Kiuchi T, Koga H, Kawamoto M, Shoji K, Sakai H, Arai Y, Ishihara G, Kawaoka S, Sugano S, Shimada T *et al* (2014) A single female-specific piRNA is the primary determiner of sex in the silkworm. *Nature* 509: 633 – 636

Larkin MA, Blackshields G, Brown NP, Chenna R, McGettigan PA, McWilliam H, Valentin F, Wallace IM, Wilm A, Lopez R *et al* (2007) Clustal W and Clustal X version 2.0. *Bioinformatics* 23: 2947 – 2948

Letunic I, Khedkar S, Bork P (2021) SMART: recent updates, new developments and status in 2020. *Nucleic Acids Res* 49: D458 – D460

Lindorff-Larsen K, Piana S, Palmo K, Maragakis P, Klepeis JL, Dror RO, Shaw DE (2010) Improved side-chain torsion potentials for the Amber ff99SB protein force field. *Proteins* 78: 1950 – 1958

© 2023 The Authors

Liu H, Wang J-YS, Huang Y, Li Z, Gong W, Lehmann R, Xu R-M (2010) Structural basis for methylarginine-dependent recognition of Aubergine by Tudor. *Genes Dev* 24: 1876 – 1881
McKinney W (2010) Data structures for statistical computing in Python. In *Proceedings of the 9th* Python in Science Conference, van der Walt S, Millman J (eds), pp 56 – 61. Austin, TX: SciPy
Mészáros B, Erdős G, Dosztányi Z (2018) IUPred2A: context-dependent prediction of protein disorder as a function of redox state and protein binding. *Nucleic Acids Res* 46: W329 – W337
Michaud-Agrawal N, Denning EJ, Woolf TB, Beckstein O (2011) MDAnalysis: a toolkit for the analysis of molecular dynamics simulations. *J Comput Chem* 32: 2319 – 2327
Mistry J, Chuguransky S, Williams L, Qureshi M, Salazar GA, Sonnhammer ELL, Tosatto SCE, Paladin L, Raj S, Richardson LJ *et al* (2021) Pfam: the protein families database in 2021. *Nucleic Acids Res* 49: D412 – D419
Mohn F, Handler D, Brennecke J (2015) Noncoding RNA. piRNA-guided slicing specifies transcripts for Zucchini-dependent, phased piRNA biogenesis. *Science* 348: 812 – 817
Murakami R, Sumiyoshi T, Negishi L, Siomi MC (2021) DEAD-box polypeptide 43 facilitates piRNA amplification by actively liberating RNA from Ago3-piRISC. *EMBO Rep* 22: e51313
Nishida KM, Iwasaki YW, Murota Y, Nagao A, Mannen T, Kato Y, Siomi H, Siomi MC (2015) Respective functions of two distinct Siwi complexes assembled during PIWI-interacting RNA biogenesis in Bombyx germ cells. *Cell Rep* 10: 193 – 203
Nishida KM, Sakakibara K, Sumiyoshi T, Yamazaki H, Mannen T, Kawamura T, Kodama T, Siomi MC (2020) Siwi levels reversibly regulate secondary piRISC biogenesis by affecting Ago3 body morphology in *Bombyx mori*. *EMBO J* 39: e105130
Nott TJ, Petsalaki E, Farber P, Jervis D, Fussner E, Plochowietz A, Craggs TD, Bazett-Jones DP, Pawson T, Forman-Kay JD *et al* (2015) Phase transition of a disordered nuage protein generates environmentally responsive membraneless organelles. *Mol Cell* 57: 936 – 947
Ohtani H, Iwasaki YW, Shibuya A, Siomi H, Siomi MC, Saito K (2013) DmGTSF1 is necessary for Piwi-piRISC-mediated transcriptional transposon silencing in the *Drosophila* ovary. *Genes Dev* 27: 1656 – 1661
Ozata DM, Gainetdinov I, Zoch A, O'Carroll D, Zamore PD (2019) PIWI-interacting RNAs: small RNAs with big functions. *Nat Rev Genet* 20: 89 – 108
Parrinello M, Rahman A (1981) Polymorphic transitions in single crystals: a new molecular dynamics method. *J Appl Phys* 52: 7182 – 7190
Piana S, Donchev AG, Robustelli P, Shaw DE (2015) Water dispersion interactions strongly influence simulated structural properties of disordered protein states. *J Phys Chem B* 119: 5113 – 5123
Podvalnaya N, Bronkhorst AW, Lichtenberger R, Hellmann S, Nischwitz E, Falk T, Karaulanov E, Butter F, Falk S, Ketting RF (2023) piRNA processing by a trimeric Schlafen-domain nuclease. *Nature* 622: 402 – 409
Rappsilber J, Ishihama Y, Mann M (2003) Stop and go extraction tips for matrix-assisted laser desorption/ionization, nanoelectrospray, and LC/MS sample pretreatment in proteomics. *Anal Chem* 75: 663 – 670
Reuter M, Chuma S, Tanaka T, Franz T, Stark A, Pillai RS (2009) Loss of the Mili-interacting Tudor domain-containing protein-1 activates transposons and alters the Mili-associated small RNA profile. *Nat Struct Mol Biol* 16: 639 – 646
Sato K, Iwasaki YW, Shibuya A, Carninci P, Tsuchizawa Y, Ishizu H, Siomi MC, Siomi H (2015) Krimper enforces an antisense bias on piRNA pools by binding AGO3 in the *Drosophila* germline. *Mol Cell* 59: 553 – 563
Schindelin J, Arganda-Carreras I, Frise E, Kaynig V, Longair M, Pietzsch T, Preibisch S, Rueden C, Saalfeld S, Schmid B *et al* (2012) Fiji: an open-source platform for biological-image analysis. *Nat Methods* 9: 676 – 682
Siomi MC, Mannen T, Siomi H (2010) How does the royal family of Tudor rule the PIWI-interacting RNA pathway? *Genes Dev* 24: 636 – 646
Siomi MC, Sato K, Pezic D, Aravin AA (2011) PIWI-interacting small RNAs: the vanguard of genome defence. *Nat Rev Mol Cell Biol* 12: 246 – 258
Smith P, Ziolek RM, Gazzarrini E, Owen DM, Lorenz CD (2019) On the interaction of hyaluronic acid with synovial fluid lipid membranes. *Phys Chem Chem Phys* 21: 9845 – 9857
Tsaban T, Varga JK, Avraham O, Ben-Aharon Z, Khramushin A, Schueler-Furman O (2022) Harnessing protein folding neural networks for peptide-protein docking. *Nat Commun* 13: 176
Vagin VV, Wohlschlegel J, Qu J, Jonsson Z, Huang X, Chuma S, Girard A, Sachidanandam R, Hannon GJ, Aravin AA (2009) Proteomic analysis of murine Piwi proteins reveals a role for arginine methylation in specifying interaction with Tudor family members. *Genes Dev* 23: 1749 – 1762
Van Roey K, Uyar B, Weatheritt RJ, Dinkel H, Seiler M, Budd A, Gibson TJ, Davey NE (2014) Short linear motifs: ubiquitous and functionally diverse protein interaction modules directing cell regulation. *Chem Rev* 114: 6733 – 6778
Waskom ML (2021) seaborn: statistical data visualization. *J Open Source Softw* 6: 3021
Waterhouse AM, Procter JB, Martin DMA, Clamp M, Barton GJ (2009) Jalview Version 2—a multiple sequence alignment editor and analysis workbench. *Bioinformatics* 25: 1189 – 1191
Webster A, Li S, Hur JK, Wachsmuth M, Bois JS, Perkins EM, Patel DJ, Aravin AA (2015) Aub and Ago3 are recruited to nuage through two mechanisms to form a Ping-Pong complex assembled by Krimper. *Mol Cell* 59: 564 – 575
Xiol J, Cora E, Koglgruber R, Chuma S, Subramanian S, Hosokawa M, Reuter M, Yang Z, Berninger P, Palencia A *et al* (2012) A role for Fkbp6 and the chaperone machinery in piRNA amplification and transposon silencing. *Mol Cell* 47: 970 – 979
Xiol J, Spinelli P, Laussmann MA, Homolka D, Yang Z, Cora E, Couté Y, Conn S, Kadlec J, Sachidanandam R *et al* (2014) RNA clamping by Vasa assembles a piRNA amplifier complex on transposon transcripts. *Cell* 157: 1698 – 1711
Yoshimura T, Watanabe T, Kuramochi-Miyagawa S, Takemoto N, Shiromoto Y, Kudo A, Kanai-Azuma M, Tashiro F, Miyazaki S, Katanaya A *et al* (2018) Mouse GTSF1 is an essential factor for secondary piRNA biogenesis. *EMBO Rep* 19: e42054
Zamparini AL, Davis MY, Malone CD, Vieira E, Zavadil J, Sachidanandam R, Hannon GJ, Lehmann R (2011) Vreteno, a gonad-specific protein, is essential for germline development and primary piRNA biogenesis in *Drosophila*. *Development* 138: 4039 – 4050
Zhang Z, Xu J, Koppetsch BS, Wang J, Tipping C, Ma S, Weng Z, Theurkauf WE, Zamore PD (2011) Heterotypic piRNA Ping-Pong requires qin, a protein with both E3 ligase and Tudor domains. *Mol Cell* 44: 572 – 584
Zhang Z, Koppetsch BS, Wang J, Tipping C, Weng Z, Theurkauf WE, Zamore PD (2014) Antisense piRNA amplification, but not piRNA production or nuage assembly, requires the Tudor-domain protein Qin. *EMBO J* 33: 536 – 539
Zhang I, Rufa DA, Pulido I, Henry MM, Rosen LE, Hauser K, Singh S, Chodera JD (2023) Identifying and overcoming the sampling challenges in relative binding free energy calculations of a model protein:protein complex. *bioRxiv* https://doi.org/10.1101/2023.03.07.530278 [PREPRINT]

License: This is an open access article under the terms of the Creative Commons Attribution License, which permits use, distribution and reproduction in any medium, provided the original work is properly cited.

 © 2023 The Authors

Expanded View Figures

Figure EV1. BmGtsf1L and BmVreteno both interact with BmAgo3.

A Domain organization (top) and ClustalW alignment of GTSF proteins from different species. The alignment and conservation scores are depicted using the Jalview software. Residues that are highlighted in blue reveal a 20% identity threshold

B Western blot detection using the mouse monoclonal anti-BmGtsf1L antibody on naïve BmN4 cell extracts or on BmN4 cells that were transfected with FLAG-BmGtsf1L.

C Validation of stable integration of FLAG-Siwi, FLAG-BmAgo3, or FLAG-eGFP expression cassettes into BmN4 cells after extensive puromycin selection by Western blot using the indicated antibodies. Anti-actin probing served as a loading control.

D Control or GFP (BmGtsf1L) immunoprecipitation on BmN4 cell extracts from FLAG-PIWI stable cells that were co-transfected with BmGtsf1L-eGFP. Western blot was performed using the indicated antibodies, and anti-actin probing as well as Ponceau S staining served as loading controls.

E Nucleotide composition of small RNAs that were sequenced from input samples or from anti-HA immunoprecipitated samples.

F Pfam/SMART-based domain organization of the BmVreteno-Long and BmVreteno-Short isoforms, showing the RNA-recognition motif (RRM), Myeloid translocation protein 8, Nervy and DEAF-1 (MYND) domain, and two C-terminal Tudor domains (TD).

G Western blot using the rabbit polyclonal anti-BmVreteno antibody on cell extracts from BmN4 cells that were either untransfected or transfected with HA-BmVreteno (L)-FL. Anti-tubulin probing served as a loading control.

H Validation of anti-BmVreteno antibody specificity on cell extracts from BmN4 cells that were transfected four consecutive times with dsRNA against Luciferase (Luc) or against BmVreteno.

I IgG or anti-BmVreteno immunoprecipitation on naïve BmN4 cells, followed by Western blot detection of endogenous BmVreteno and BmAgo3.

J Reciprocal IP on BmN4 cell extracts using non-immune (n.i.) serum or anti-BmAgo3 antibodies as well as endogenous BmVreteno antibodies for Western blot detection of retrieved proteins.

© 2023 The Authors

© 2023 The Authors

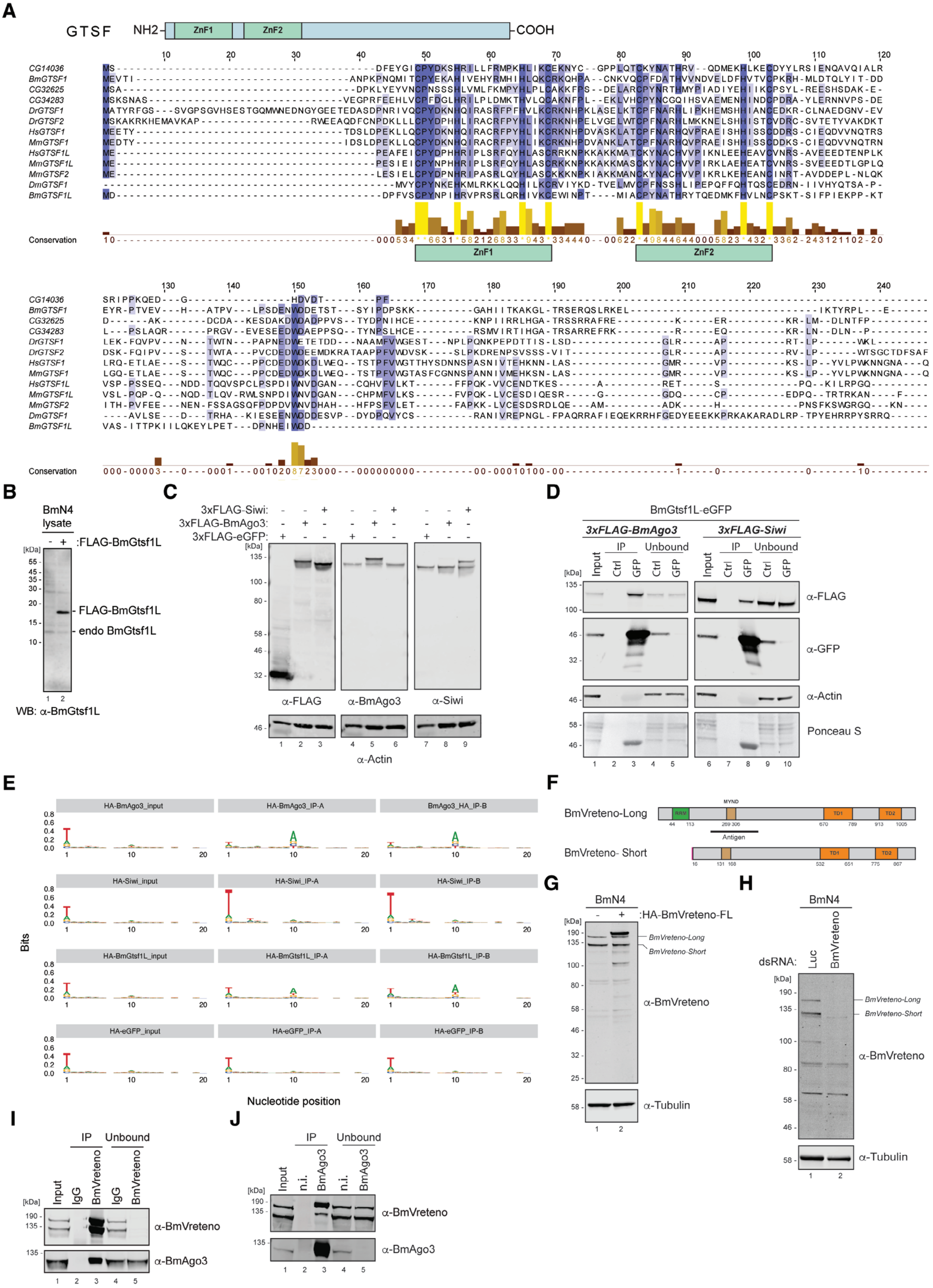

Figure EV1.

 © 2023 The Authors

Figure EV2. BmAgo3 interacts with BmVreteno eTD1 via methylated arginine residues.

A Fluorescence lifetime imaging of BmN4 cells (using all channels) transfected with either eGFP-BmVreteno, BmGtsf1L- mOrange2, or mCardinal-BmAgo3. Inset shows the zoom-in of the boxed area. Two representative images of two biological experiments are shown. Scale bars: 10 μm. Plots on the right show the normalized intensity (gray values) for each channel of the line that has been drawn in the inset frame. Quantification data can be found in Dataset EV4.

B Multiple sequence alignments of Tudor domains expressed in BmVreteno and its orthologue in *Drosophila* (DmVret). In addition, eTudor11 from the *Drosophila* Tudor protein as well as the eTudor domain of *Drosophila* Tudor-SN (p100), for which crystal structures have been resolved (PDB: 3NTH and 2WAC, respectively), were included. Alignments were performed using Clustal Omega and processed using Jalview software. Aromatic cage residues are depicted as green boxes, and the asparagine residue that is involved in directly binding to the methylated arginine residue (sDMA) is highlighted in yellow. Identical residues (*), conserved substitutions (:) or substitutions by weakly similar residues (.) are indicated below the alignment.

C Anti-FLAG immunoprecipitation of FLAG-BmAgo3 variants that were transiently expressed in the HA-BmGtsf1L-eGFP stable BmN4 cell line. The transfection of 3xFLAG-mCherry served as a control. Immunoprecipitated proteins were analyzed by Western blot using the indicated antibodies, whereas Ponceau S staining served as a loading control.

© 2023 The Authors

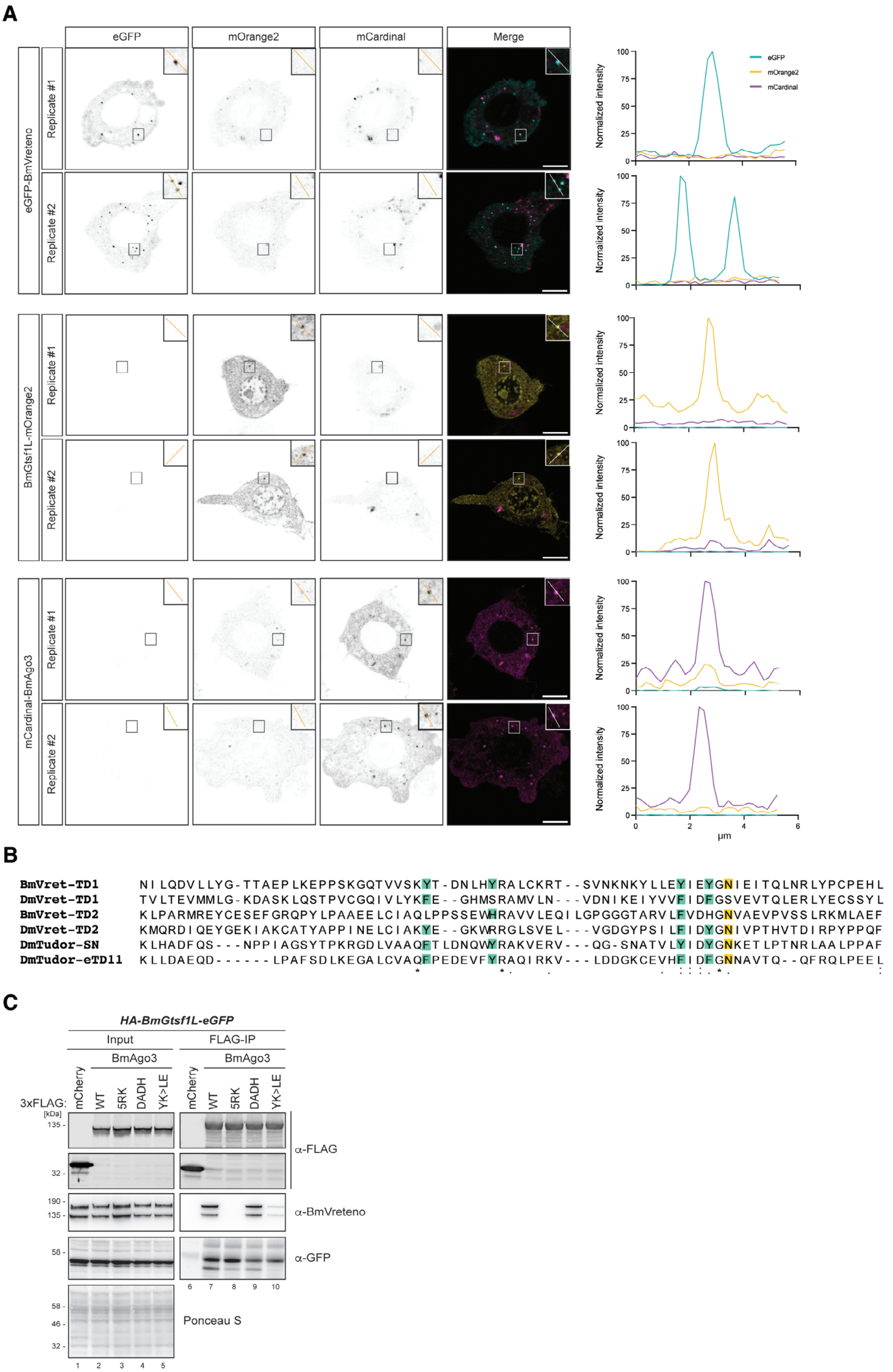

Figure EV2.

© 2023 The Authors

Figure EV3. The BmGtsf1L C-terminus establishes an interaction with BmVreteno.

A Outlined strategy for the purification of recombinant GST-tagged BmVreteno-S, showing the profiles from the size-exclusion column (left) and the peak fractions that were analyzed by SDS-PAGE followed by Coomassie staining (right).
B Similar to panel (A), but for GST-BmVreteno-L.
C Size-exclusion profiles of BmVreteno-S (left) and BmVreteno-L (right) to compare the shift in molecular weight before (green line) and after (blue line) 3C-mediated cleavage of the GST tag.
D Pfam/SMART-based domain organization of the BmVreteno-Long isoform, showing the RNA-recognition motif (RRM), Myeloid translocation protein 8, Nervy and DEAF-1 (MYND) domain, and two C-terminal Tudor domains (TD). In addition to the full-length (FL) BmVreteno, the C-and N-terminal truncation variants are depicted and are used in panel (E) to analyze the interaction between BmGtsf1L and BmVreteno variants.
E Co-transfection of BmGtsf1L-eGFP with HA-BmVreteno truncation variants in which different domains were omitted. The transfection of HA-LacZ served as a control. BmGtsf1L was retrieved by GFP-IP, and input and elution fractions were analyzed by SDS-PAGE, followed by Western blot using the indicated antibodies. Anti-actin immunodetection served as a loading control.

© 2023 The Authors

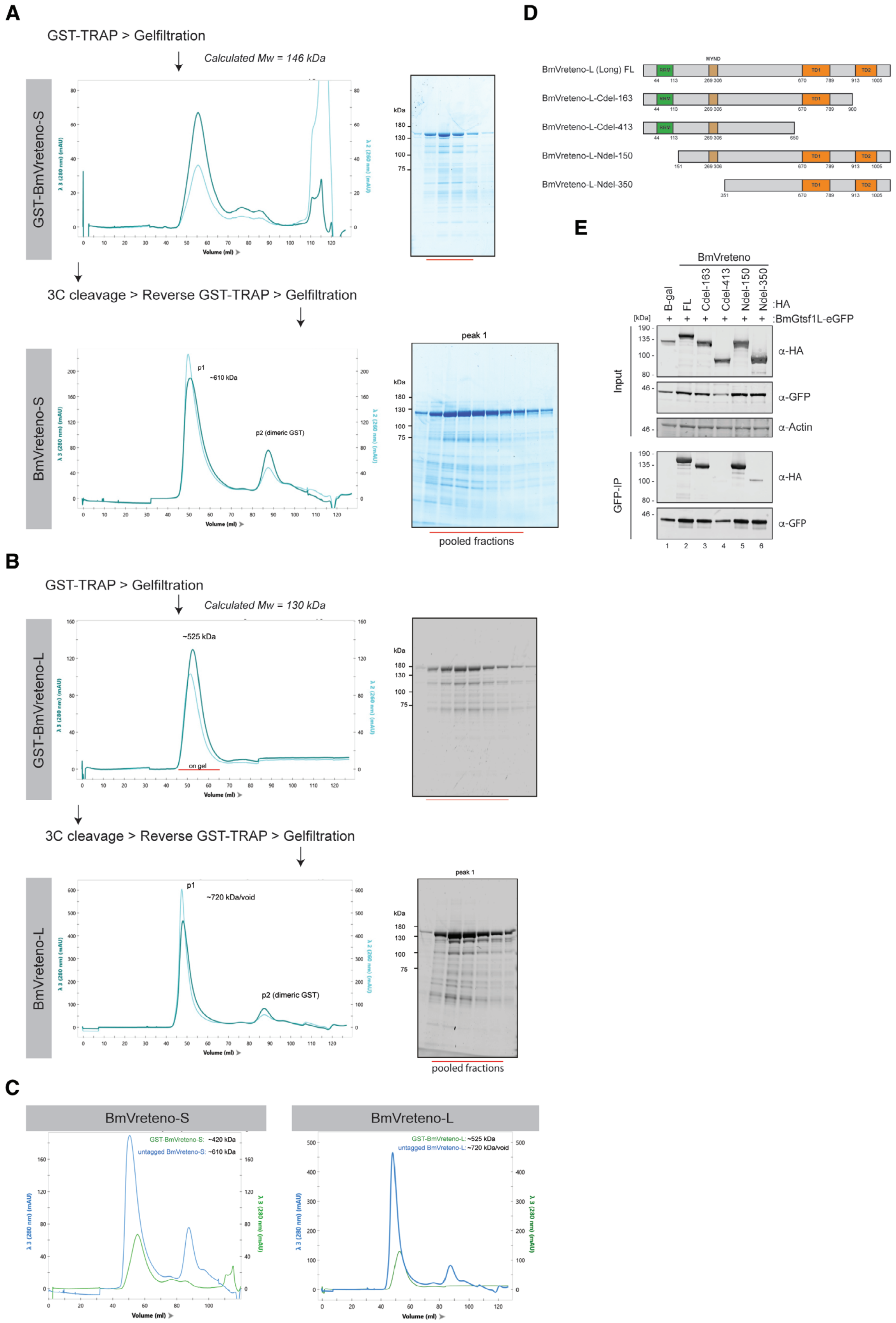

Figure EV3.

© 2023 The Authors

Figure EV4. The hydrophobic binding pocket is unique to BmVreteno AF-eTD1 and facilitates BmGtsf1L binding.

A Superimposition of the AlphaFold structural models of all three BmVreteno AF-eTD domains. The central inset (closed circle) shows the side view of the aromatic cage, which is only present in AF eTD1 and indicated with a dashed circle. The top inset (closed circle) shows a top view of the novel interface, which is unique to AF eTD1. The hydrophobic pocket is indicated with a dashed circle, and the enlarged view additionally shows the docked C-terminal motif of BmGtsf1L.

B Snapshot on the novel hydrophobic binding pocket of BmVreteno AF-eTD1 (blue) and contacts between the residues R742, S744, K749, and I762 (shown as sticks) with BmGtsf1L C-terminal 10-AA residues (shown as yellow sticks). This snapshot displays the BmVreteno S744 residue that forms a hydrogen bond with the backbone carbonyl of BmGtsf1L I98.

C Plots showing the distances between the atoms forming the four most important inter-chain hydrogen bonds of the side chain of BmVreteno S744 in two out of the ten 1 μs simulation runs. The run presented in the upper panel displays the hydrogen bond between the side chain of S744 and the backbone carbonyl of BmGtsf1L I98. The simulation presented in the bottom panel reveals that S744 is engaged in different interactions with BmGtsf1L residues I98 and D100. Overall, the simulations reveal that some of the hydrogen bonds are transiently formed and broken.

D Single-plane confocal micrographs of BmN4 cells co-transfected with different eGFP-BmVreteno constructs (upper panel) and BmGtsf1L-mCherry (middle panel). Yellow triangles indicate a formed granule. Scale bars: 4 μm.

E Transfection of BmN4 cells with BmGtsf1L-eGFP together with HA-BmVreteno-FL. The transfection of HA-LacZ served as a control. A GFP (BmGtsf1L) immunoprecipitation was performed on BmN4 lysates, and input as well as elution samples were resolved by SDS-PAGE. Proteins were detected by Western blot using the indicated antibodies, and anti-actin probing as well as Ponceau S staining served as a loading control.

© 2023 The Authors

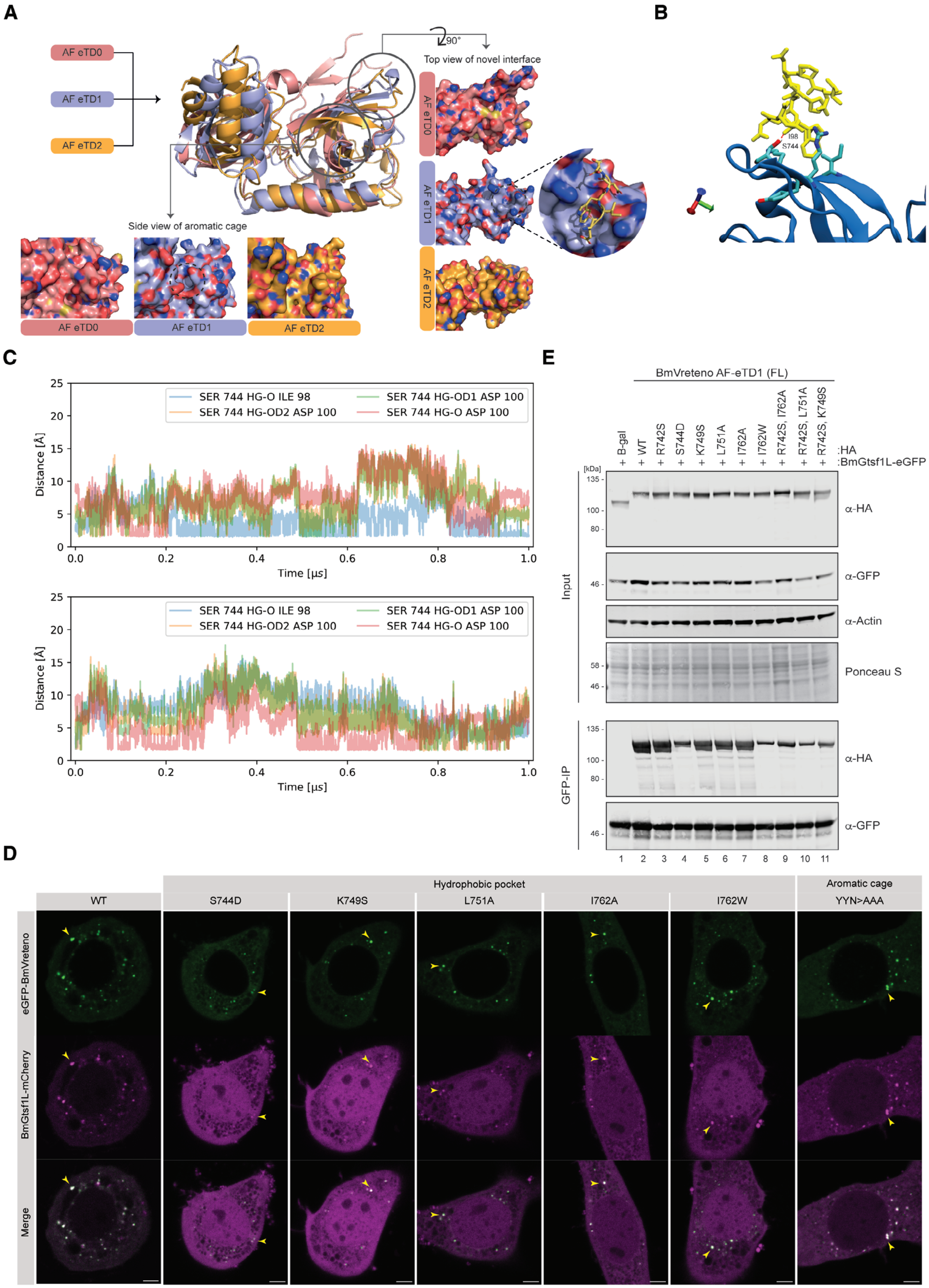

Figure EV4.

 © 2023 The Authors
