# Supplementary material for: An extended Tudor domain within Vreteno interconnects Gtsf1L and Ago3 for piRNA biogenesis in Bombyx mori
Source: EMBO J. 2023 Nov 20;42(24):e114072. doi: 10.15252/embj.2023114072 (PMC10711660; doi:10.15252/embj.2023114072)
Supplement: Supplementary file 9 — Source Data for Figure 1 [file EMBJ-42-e114072-s005.zip › Figure 1/1K/ReadMe_Stellaris.rtf]

Confocal imaging was performed using a STELLARIS 8 FALCON microscope (Leica Microsystems, Mannheim, Germany) equipped with a White Light Laser (WLL). Images (512 x 512 pixel format, pixel size 180 nm) were acquired with a 63x/1.40NA oil immersion objective, using:Channel 0: 488 nm excitation line and the emission band ranging from 500 nm to 540 nm using a detector HyD X2; Channel 1: 548 nm excitation line and the emission band ranging from 560 nm to 590 nm using a detector HyD X4; Channel 2: 600 nm excitation line and the emission band ranging from 620 nm to 750 nm using a detector HyD R5. Sequential scan was performed line by line and accumulation mode was set to 300 to have enough photon counts for fluorescence lifetime imagining.Raw images were exported as OME Tiff, fixed range of “Intensity Counts”, per Gray Level: 1 
