# Supplementary figures and images for: An extended Tudor domain within Vreteno interconnects Gtsf1L and Ago3 for piRNA biogenesis in Bombyx mori
Source: EMBO J. 2023 Nov 20;42(24):e114072. doi: 10.15252/embj.2023114072 (PMC10711660; doi:10.15252/embj.2023114072)

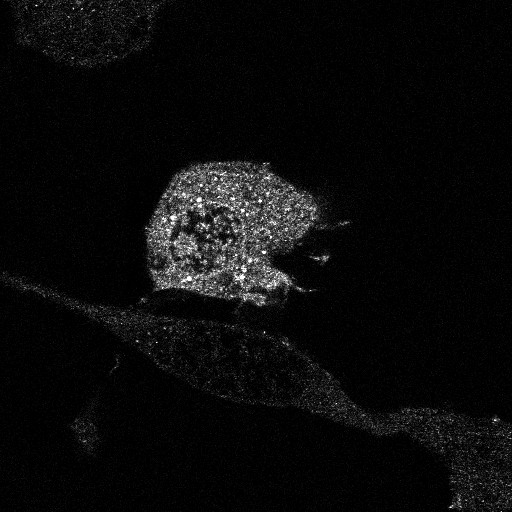

Supplement: Supplementary file 9 — Source Data for Figure 1 [file EMBJ-42-e114072-s005.zip › Figure 1/1K/triple9_ch1.ome.tif]

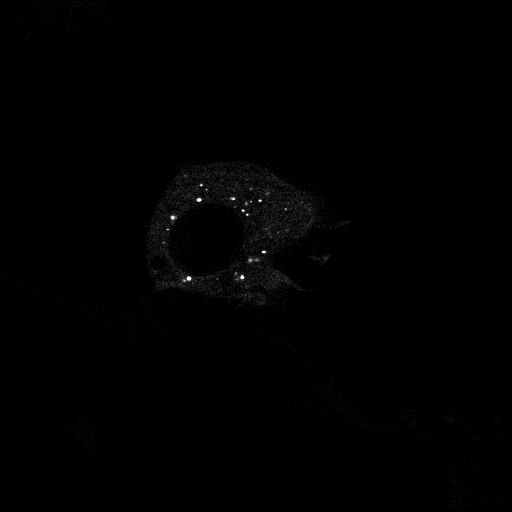

Supplement: Supplementary file 9 — Source Data for Figure 1 [file EMBJ-42-e114072-s005.zip › Figure 1/1K/triple9_ch0.ome.tif]

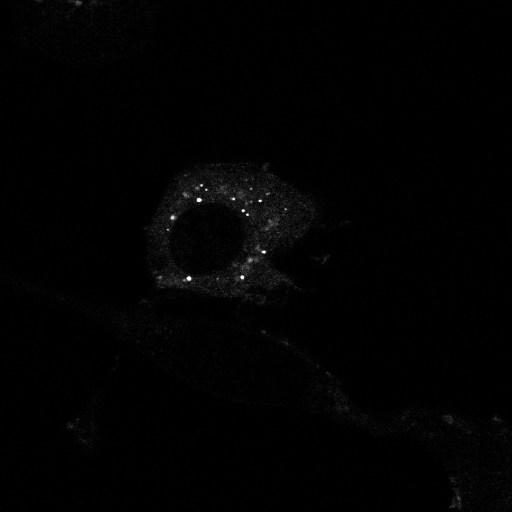

Supplement: Supplementary file 9 — Source Data for Figure 1 [file EMBJ-42-e114072-s005.zip › Figure 1/1K/triple9_ch2.ome.tif]

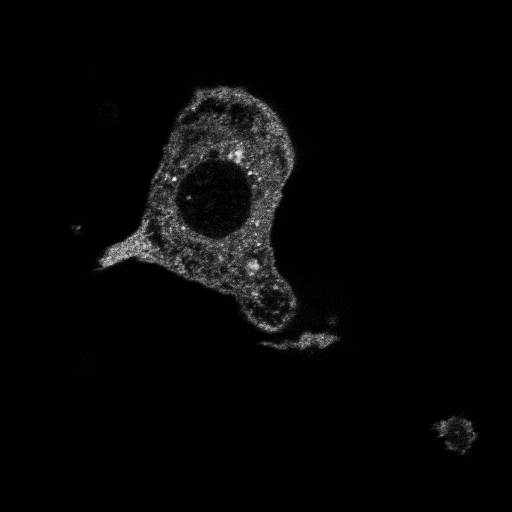

Supplement: Supplementary file 9 — Source Data for Figure 1 [file EMBJ-42-e114072-s005.zip › Figure 1/1K/triple7_ch2.ome.tif]

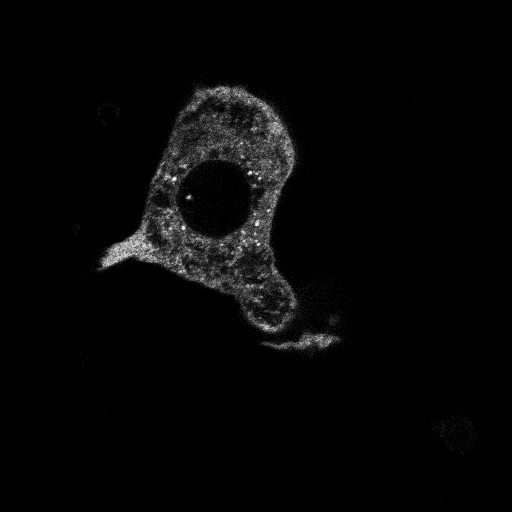

Supplement: Supplementary file 9 — Source Data for Figure 1 [file EMBJ-42-e114072-s005.zip › Figure 1/1K/triple7_ch0.ome.tif]

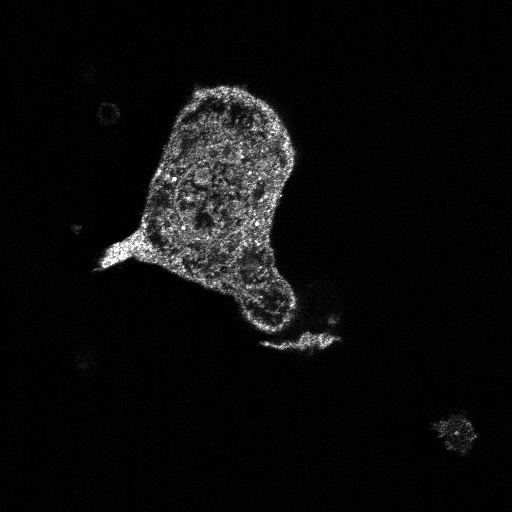

Supplement: Supplementary file 9 — Source Data for Figure 1 [file EMBJ-42-e114072-s005.zip › Figure 1/1K/triple7_ch1.ome.tif]
