# Supplementary material for: An extended Tudor domain within Vreteno interconnects Gtsf1L and Ago3 for piRNA biogenesis in Bombyx mori
Source: EMBO J. 2023 Nov 20;42(24):e114072. doi: 10.15252/embj.2023114072 (PMC10711660; doi:10.15252/embj.2023114072)
Supplement: Supplementary file 14 [file EMBJ-42-e114072-s006.pdf]

THE EMBO JOURNAL

An extended Tudor domain within Vreteno connects Gtsf1L and Ago3 for piRNA biogenesis in Bombyx mori

Alfred Willem Bronkhorst, Katja Luck, Lukas Stelzl, Rene Ketting, Shéraz Sadouki, Martin Moeckel, Sabine Ruegenberg, Chop Yan Lee, Antonio de Jesus Domingues, Tetsutar Sumiyoshi, Mikiko Siomi, and Rossana Piccinno
DOI: 10.15252/embj.2023114072

Corresponding authors: Alfred Willem Bronkhorst (awbronkhorst@gmail.com) , Rene Ketting (r.ketting@imb-mainz.de), Katja Luck (k.luck@imb-mainz.de)

Review Timeline:

Submission Date:	22nd Mar 23
Editorial Decision:	19th Apr 23
Revision Received:	30th Sep 23
Editorial Decision:	11th Oct 23
Revision Received:	25th Oct 23
Accepted:	30th Oct 23

Editor: Ieva Gailite

Transaction Report:

(Note: With the exception of the correction of typographical or spelling errors that could be a source of ambiguity, letters and reports are not edited. Depending on transfer agreements, referee reports obtained elsewhere may or may not be included in this compilation. Referee reports are anonymous unless the Referee chooses to sign their reports.)

1st Editorial Decision 19th Apr 2023

Dear Dr. Bronkhorst,

Thank you for submitting your manuscript for consideration by the EMBO Journal. We have now received comments from two reviewers, which are included below for your information.

As you will see from the reports, while reviewer #1 is more positive in their assessment, reviewer #2 raises several concerns regarding the data analysis and interpretation, and most importantly indicates that evidence for the relevance of the reported interactions for piRISC activity and transposable element silencing would be needed, especially keeping in mind earlier studies reporting the interaction between Gstf1L and Ago3 and its role in promoting Ago3 activity. From my side, I find these points reasonable and important to address in the revision. I think it would be helpful to discuss the revision in more detail via email or phone/videoconferencing. I should also add that it is The EMBO Journal policy to allow only a single major round of revision and that it is therefore important to resolve the main concerns at this stage.

We generally allow three months as standard revision time. As a matter of policy, competing manuscripts published during this period will not negatively impact on our assessment of the conceptual advance presented by your study. However, please contact me as soon as possible upon publication of any related work to discuss the appropriate course of action. Should you foresee a problem in meeting this three-month deadline, please let us know in advance to discuss an extension.

When preparing your letter of response to the referees' comments, please bear in mind that this will form part of the Review Process File and will therefore be available online to the community. For more details on our Transparent Editorial Process, please visit our website: https://www.embopress.org/page/journal/14602075/authorguide#transparentprocess. Please also see the attached instructions for further guidelines on preparation of the revised manuscript.

Please feel free to contact me if you have any further questions regarding the revision. Thank you for the opportunity to consider your work for publication. I look forward to discussing your revision.

With best regards,

Ieva

Ieva Gailite, PhD
Senior Scientific Editor
The EMBO Journal
Meyerhofstrasse 1
D-69117 Heidelberg
Tel: +4962218891309
i.gailite@embojournal.org

Instructions for preparing your revised manuscript:

Please make sure you upload a letter of response to the referees' comments together with the revised manuscript.

Please also check that the title and abstract of the manuscript are brief, yet explicit, even to non-specialists.

When assembling figures, please refer to our figure preparation guideline in order to ensure proper formatting and readability in print as well as on screen:
https://bit.ly/EMBOPressFigurePreparationGuideline
See also guidelines for figure legends: https://www.embopress.org/page/journal/14602075/authorguide#figureformat

At EMBO Press we ask authors to provide source data for the main manuscript figures. Our source data coordinator will contact you to discuss which figure panels we would need source data for and will also provide you with helpful tips on how to upload and organize the files.

IMPORTANT: When you send the revision we will require
- a point-by-point response to the referees' comments, with a detailed description of the changes made (as a word file).
- a word file of the manuscript text.
- individual production quality figure files (one file per figure)
- a complete author checklist, which you can download from our author guidelines (https://www.embopress.org/page/journal/14602075/authorguide).

- Expanded View files (replacing Supplementary Information)
Please see out instructions to authors
https://www.embopress.org/page/journal/14602075/authorguide#expandedview

Please remember: Digital image enhancement is acceptable practice, as long as it accurately represents the original data and conforms to community standards. If a figure has been subjected to significant electronic manipulation, this must be noted in the figure legend or in the 'Materials and Methods' section. The editors reserve the right to request original versions of figures and the original images that were used to assemble the figure.

Further information is available in our Guide For Authors: https://www.embopress.org/page/journal/14602075/authorguide

We realize that it is difficult to revise to a specific deadline. In the interest of protecting the conceptual advance provided by the work, we recommend a revision within 3 months (18th Jul 2023). Please discuss the revision progress ahead of this time with the editor if you require more time to complete the revisions.

Referee #1:

PIWI proteins and their associated PIWI-interacting RNAs identify target RNAs for silencing. One mechanism is the piRNA-guided slicer endonuclease activity of PIWI that cleaves the target RNA. Recently, GTSF1 was identified as a protein that stimulates slicer activity of PIWI proteins, but their association is weak, requiring very high molar excess of GTSF1 over the PIWI protein for the stimulatory activity in vitro. Can this interaction be functional in vivo where their concentrations are much lower? Or is their interaction facilitated by a third party? This is what this beautiful manuscript from Bronkhorst et al. deals with. In this manuscript the authors identify a role for a Tudor domain protein Vreteno in bridging the PIWI protein (Ago3) and GTSF1. They show that a homolog GTSF1L in silkworm cell line BmN4 associates with several piRNA pathway factors, including Ago3 and the tudor domain protein Vreteno. The three proteins co-localize in cytoplasmic granules called nuage. Interestingly, GTSF1L is also nuclear, as also previously shown for Drosophila GTSF1L. The authors find that a C-terminal region of GTSF1L interacts with Vreteno and this interaction is direct, as show using recombinant proteins. It is shown that GTSF1L interacts with one (eTD1) of the three extended Tudor domains predicted (AlphaFold) to be present in Vreteno. Curiously, their experiments reveal that the first tudor domain can bind both Ago3 and GTSF1L, but using different regions. This study makes clever use of cell biology, protein biochemistry and structure predictions that are then validated by clean experiments. This work brings out a very important information regarding Tudor domain proteins. We already knew that Tudor domains can interact with PIWI proteins: modified arginines on the PIWI binding to the pocket in the Tudor domain. Now we learn that, by providing an additional binding site on the same Tudor domain, the Tudor protein (Vreteno) has an additional role in bringing the cleavage stimulatory factor GTSF1L into close proximity to Ago3. This highlights the regulatory role Tudor domains can play in controlling the slicer activity of PIWI proteins. The results in this manuscript is of great significance for the piRNA field.

I support its publication after they have addressed the following minor concerns.

1. Page 5, Line 27: Cite Izumi et al. (2022) in this part to introduce BmGtsf1L.
2. Page 5, Line 28: The hypothesis/interest in this question is interesting. In the mouse GTSF1/MIWI context, it is clear that GTSF1 relaxes pairing rules (in vitro). Therefore, I like the idea that with the involvement of a Tudor intermediary, we might go back to more selection and stringency. Interesting. Not relevant for this manuscript, but the field should check this in vitro, using purified components.
3. What is the purpose of change the loading control between actin and tubulin? And why is Actin being recovered in the IP fractions in Figure 2A? If it is not actin (maybe antibody chain?), then mark it appropriately.
4. Page 16, Line 4: This is not the correct reference because Arif et al does not characterize BmGtsf1L function. Or is there something in the supplement of that study?
5. Some sentences are too long and confusing.
6. Consistently put protein sizes in WB and IP figures (present partially in Figure 1, but not in Figure 2, and present again in Figure 3,4, and 5).
7. The authors sometimes pull down Siwi with BmGtsf1L and sometimes not (Figure 1B, D-F). They could discuss these results in the discussion section where they comment on the recruitment of Siwi into the Vreteno complex (page 16, line 15 onwards).

Referee #2:

General comments

The manuscript by Bronkhorst et al describes how silkworm Gtsf1L, a Gtsf1 paralog, that binds piRNA-loaded BmAgo3 and BmVreteno. Those interactions were identified firstly by quantitative mass-spectrometry and verified by IP with the tagged BmGtsf1L and the antibodies against BmAgo3 and BmVreteno. Direct interaction was also examined using those purified proteins in vitro, in addition to the localization of these proteins in BmN4 cells. The domain analysis revealed that the C terminal part of BmGtsf1L is essential for binding to BmAgo3 and BmVreteno. Detailed AlphaFold predictions and optimization of the structure by dynamics simulation of BmVreteno revealed that the newly identified eTudor domain and the amino residues which were responsible for binding to BmGtsf1L.

The predictions of protein structure were conducted in a proper manner, and their validation was properly performed. However, most of the results focus on the protein-protein interaction. This study remains in biochemical aspects of Gtsf1L in silkworm and fails to fully demonstrate the biological significance of Gtsf1L function in piRNA pathway. As rightly but shortly documented in Introduction, Tomari's group already reported that Gtsf1 predominantly bound to Siwi, and Gtsf1L did so to Ago3 in last year (doi: 10.1261/rna.079380.122). They also showed that Gtsf1 and Gtsf1L promoted cleavages of piRNA precursors by Siwi and Ago3. Efficiencies of cleavage promoted by Gtsf1 and Gtsf1L were correlated with their binding capabilities to Piwi and Ago3, suggesting that Gtsf1 and Gtsf1L specifically interact with Siwi and Ago3 to promote their cleavage activity. Importantly, they showed Gtsf1 and Gtsf1L functioned for piRNA production and ping-pong amplification not only in BmN4 but also in vivo.

In order to further advance understanding in this field, the molecular mechanism how the proper interaction of Gtsf1L with Vreteno and Ago3 functions for in ping-pong cycle should be presented. This study needs address how the disruption of interaction of these proteins affects the activity of piRiSC- BmAgo3, for the cleavage and ping-pong during piRNA biogenesis as well as TE repression in vitro (or in BmN4) and in vivo. This manuscript would be suitable for the more specific journal, rather EMBO Journal, since biochemical analysis and back-to-back approach between prediction and validation are well presented.

Specific major concerns

1. In P7_L29 -P8_L2, the authors claim that BmGtsf1L- associated small RNA profiles resembled those of BmAgo3 bound small RNAs: a strong sense-bias and enrichment for adenine at the 10th position and conclude that BmGtsf1L associates with piRNA-loaded BmAgo3. However, the score does not show so strong a sense-bias, but relatively milder. In addition, there are enrichment not only for adenine at the 10th position but also T at the first position. This result implies a contribution of not only sense but also antisense strand piRNAs on the complex with Ago3, albeit to lesser extent than that with Siwi. Is it due to background or unspecific binding to the antibody and/or beads which can be seen in GFP alone control? How do the authors interpret such strong binding of piRNAs and/or the complexes in GFP control? Would it be possible to subtract those populations from Siwi- and Ago3- bound piRNAs?

2. The stoichiometry of the interaction and localization of the three proteins, BmGtsf1L, BmAgo3, and BmVreteno are not so clear. Fig1F, I, and J show a relatively large amount of BmGstf1 binds to the BmAgo3 and BmVreteno, but the Fig1K shows substantial amount of BmGstf1in the nucleus even in the presence of BmAgo3. Does the cytoplasmic population also interact with each other beside of in the granules? The merged images should be better to be convincing.

3. Co-expression of BmAgo3 and BmVreteno make more granules structure, but the other combinations did not. Also, it is hard to say that BmGtsf1L makes granules dominantly with BmAgo3 and BmVreteno due to the frequency of granule and their dispersed cytoplasmic distribution. To make this point clear, the authors should examine all three proteins together for their co-localization.

4. Although the authors claim the interaction between BmGtsf1 and BmAgo3 under BmVreteno-KD, the signal with anti-GFP for Gtsf1 in Ago3 IP (Fig2E) is quite faint.

5. As mentioned in the general comments, the biological importance of these protein interaction needs to be shown. The authors could examine the activity for these complex and relevancy of the interaction for piRNA processing, i.e. the activity of the proper or malformed piRiSC, and TE repression. Is proper interaction between Gtsf1L and Vreteno required for Ago3 activity but not Siwi's? How their complex formation and piRNA loading are affected? Is it also possible to examine this point by checking their localization as well?

6. In Fig3DE: the morphology and size of the cells expressing BmGstf1 w99A differ from the wild type. The viability and healthiness of the cell line upon expressing Gstf1 w99A should be shown. It is needed for SupFigS4 in the series of the mutants of the hydrophobic pocket.

Minor concerns

1. Fig1B experiment appearently uses s-BmVreteno, and RNase treatment did not affect the interaction with Gtsf1L. I wonder how is that with L-BmVreteno containing RRM domain.
2. Change the capital for the figure legend to be consistent with Fig a→A.
3. In Fig5A heatmap (bottom), it should be BmGtsf1, not BmGtsf2.
4. Use the serial numbers for the lines: each page starts with line1 and it's confusing.

We thank the reviewers for their time in evaluating our manuscript and for providing constructive comments. We address their comments below. We hope that these revisions address the comments of the reviewers in a satisfactory manner.

Referee #1:

PIWI proteins and their associated PIWI-interacting RNAs identify target RNAs for silencing. One mechanism is the piRNA-guided slicer endonuclease activity of PIWI that cleaves the target RNA. Recently, GTSF1 was identified as a protein that stimulates slicer activity of PIWI proteins, but their association is weak, requiring very high molar excess of GTSF1 over the PIWI protein for the stimulatory activity in vitro. Can this interaction be functional in vivo where their concentrations are much lower? Or is their interaction facilitated by a third party? This is what this beautiful manuscript from Bronkhorst et al. deals with. In this manuscript the authors identify a role for a Tudor domain protein Vreteno in bridging the PIWI protein (Ago3) and GTSF1. They show that a homolog GTSF1L in silkworm cell line BmN4 associates with several piRNA pathway factors, including Ago3 and the tudor domain protein Vreteno. The three proteins co-localize in cytoplasmic granules called nuage. Interestingly, GTSF1L is also nuclear, as also previously shown for Drosophila GTSF1L. The authors find that a C-terminal region of GTSF1L interacts with Vreteno and this interaction is direct, as show using recombinant proteins. It is shown that GTSF1L interacts with one (eTD1) of the three extended Tudor domains predicted (AlphaFold) to be present in Vreteno. Curiously, their experiments reveal that the first tudor domain can bind both Ago3 and GTSF1L, but using different regions. This study makes clever use of cell biology, protein biochemistry and structure predictions that are then validated by clean experiments. This work brings out a very important information regarding Tudor domain proteins. We already knew that Tudor domains can interact with PIWI proteins: modified arginines on the PIWI binding to the pocket in the Tudor domain. Now we learn that, by providing an additional binding site on the same Tudor domain, the Tudor protein (Vreteno) has an additional role in bringing the cleavage stimulatory factor GTSF1L into close proximity to Ago3. This highlights the regulatory role Tudor domains can play in controlling the slicer activity of PIWI proteins. The results in this manuscript is of great significance for the piRNA field.

I support its publication after they have addressed the following minor concerns.

1. Page 5, Line 27: Cite Izumi et al. (2022) in this part to introduce BmGtsf1L.
We have now included this reference.

2. Page 5, Line 28: The hypothesis/interest in this question is interesting. In the mouse GTSF1/MIWI context, it is clear that GTSF1 relaxes pairing rules (in vitro). Therefore, I like the idea that with the involvement of a Tudor intermediary, we might go back to more selection and stringency. Interesting. Not relevant for this manuscript, but the field should check this in vitro, using purified components.
We agree with the reviewer. Unfortunately, to our knowledge no lab managed to generate recombinant Ago3 needed to test this idea.

3. What is the purpose of change the loading control between actin and tubulin? And

why is Actin being recovered in the IP fractions in Figure 2A? If it is not actin (maybe antibody chain?), then mark it appropriately.
There is no specific reason why either actin or tubulin is used as a loading control. Regarding Figure 2A: Actin is not recovered in the IP lanes, but this is indeed the light chain of the anti-BmVreteno antibody that was also raised in rabbits. We now changed this in the figure panel and indicated the antibody light-chain with an asterisk (as well as for panel 2B and 2E).

4. Page 16, Line 4: This is not the correct reference because Arif et al does not characterize BmGtsf1L function. Or is there something in the supplement of that study?
This paper did in fact address whether BmGtsf1L can enhance slicing activity of BmAgo3 (immunopurified from BmN4 cells and thus loaded with endogenous piRNAs), as can be seen in Fig. S6A of Arif et al., (Nature 2022). In this study, BmGtsf1 shows a slight increase in Siwi slicing activity (but not for BmAgo3) and there is a very minor effect of **BmGtsf1L** on BmAgo3-piRISC activity on two different targets. Moreover, Izumi et al., also show a very small stimulatory effect of BmGtsf1L on BmAgo3 target cleavage (Izumi et al., RNA 2022). We now also included this reference.

5. Some sentences are too long and confusing.
We re-wrote some of the sentences and hope it will be more concise and clearer.

6. Consistently put protein sizes in WB and IP figures (present partially in Figure 1, but not in Figure 2, and present again in Figure 3,4, and 5).
We now indicated protein sizes for all relevant figure panels.

7. The authors sometimes pull down Siwi with BmGtsf1L and sometimes not (Figure 1B, D-F). They could discuss these results in the discussion section where they comment on the recruitment of Siwi into the Vreteno complex (page 16, line 15 onwards).
We believe we retrieve Siwi as a tertiary interaction, which will most likely be empty Siwi. It is known that BmAgo3 can also co-precipitate Siwi (Figure 1E). However, in overexpression settings we clearly retrieve more Siwi. We now discussed the retrieval of Siwi from BmGtsf1L IPs also in the discussion section, as suggested by the reviewer.

Referee #2:

General comments
The manuscript by Bronkhorst et al describes how silkworm Gtsf1L, a Gtsf1 paralog, that binds piRNA-loaded BmAgo3 and BmVreteno. Those interactions were identified firstly by quantitative mass-spectrometry and verified by IP with the tagged BmGtsf1L and the antibodies against BmAgo3 and BmVreteno. Direct interaction was also examined using those purified proteins in vitro, in addition to the localization of these proteins in BmN4 cells. The domain analysis revealed that the C terminal part of

BmGtsf1L is essential for binding to BmAgo3 and BmVreteno. Detailed AlphaFold predictions and optimization of the structure by dynamics simulation of BmVreteno revealed that the newly identified eTudor domain and the amino residues which were responsible for binding to BmGtsf1L.

The predictions of protein structure were conducted in a proper manner, and their validation was properly performed. However, most of the results focus on the protein-protein interaction. This study remains in biochemical aspects of Gtsf1L in silkworm and fails to fully demonstrate the biological significance of Gtsf1L function in piRNA pathway. As rightly but shortly documented in Introduction, Tomari's group already reported that Gtsf1 predominantly bound to Siwi, and Gtsf1L did so to Ago3 in last year (doi: 10.1261/rna.079380.122). They also showed that Gtsf1 and Gtsf1L promoted cleavages of piRNA precursors by Siwi and Ago3. Efficiencies of cleavage promoted by Gtsf1 and Gtsf1L were correlated with their binding capabilities to Piwi and Ago3, suggesting that Gtsf1 and Gtsf1L specifically interact with Siwi and Ago3 to promote their cleavage activity. Importantly, they showed Gtsf1 and Gtsf1L functioned for piRNA production and ping-pong amplification not only in BmN4 but also in vivo.

In order to further advance understanding in this field, the molecular mechanism how the proper interaction of Gtsf1L with Vreteno and Ago3 functions for in ping-pong cycle should be presented. This study needs address how the disruption of interaction of these proteins affects the activity of piRiSC- BmAgo3, for the cleavage and ping-pong during piRNA biogenesis as well as TE repression in vitro (or in BmN4) and in vivo. This manuscript would be suitable for the more specific journal, rather EMBO Journal, since biochemical analysis and back-to-back approach between prediction and validation are well presented.

Specific major concerns

1. In P7_L29 -P8_L2, the authors claim that BmGtsf1L- associated small RNA profiles resembled those of BmAgo3 bound small RNAs: a strong sense-bias and enrichment for adenine at the 10th position and conclude that BmGtsf1L associates with piRNA-loaded BmAgo3. However, the score does not show so strong a sense-bias, but relatively milder. In addition, there are enrichment not only for adenine at the 10th position but also T at the first position. This result implies a contribution of not only sense but also antisense strand piRNAs on the complex with Ago3, albeit to lesser extent than that with Siwi. Is it due to background or unspecific binding to the antibody and/or beads which can be seen in GFP alone control? How do the authors interpret such strong binding of piRNAs and/or the complexes in GFP control? Would it be possible to subtract those populations from Siwi- and Ago3- bound piRNAs? ,
The weaker enrichment of BmAgo3 characteristics in the BmGtsf1L IP and the observed piRNAs in the GFP control are likely linked issues. The GFP IP control basically shows the same as the input, and results from the fact that our washes were not 100% removing non-specifically bound proteins. In general, it is not possible to subtract such control signals from the experimental IPs because in these workflows the available sequence space will simply be filled by whatever is present. Hence, absolute quantification between libraries is impossible with our approach, which lacks spike-ins. This control simply shows what sort of background we are

dealing with. In this case the background will be predominantly Siwi-bound piRNAs, as they dominate the input.
Then, when we compare BmAgo3 with BmGtsf1L IPs, it should be noted that in general not all BmAgo3 comes down in BmGtsf1L IPs. Therefore, in BmGtsf1L IPs the background will be stronger than in BmAgo3 IPs, resulting in more Siwi-like signal (i.e. 1U and 10A) compared to the BmAgo3 IP.
Finally, the BmAgo3 and Siwi IPs were done in higher salt concentrations, because of known co-IP of the two PIWI proteins. For BmGtsfL this was not possible due to disruption of interactions. We have provided this information now also in the Materials and Methods section.
Overall, we believe that we can maintain our conclusion that BmGtsf1L IPs retrieve mainly loaded BmAgo3, and not loaded Siwi.

2. The stoichiometry of the interaction and localization of the three proteins, BmGtsf1L, BmAgo3, and BmVreteno are not so clear. Fig1F, I, and J show a relatively large amount of BmGstf1 binds to the BmAgo3 and BmVreteno, but the Fig1K shows substantial amount of BmGstf1in the nucleus even in the presence of BmAgo3. Does the cytoplasmic population also interact with each other beside of in the granules? The merged images should be better to be convincing.
We would like to note that the immunoprecipitations were performed on cell lysates (using ~ 1-4 mg of total protein lysate), whereas the microscopy images represent an individual cell, and therefore one cannot make statements on stoichiometry
While overexpressed BmAgo3, BmVreteno and BmGtsf1L co-localize to granules, a substantial fraction is indeed also dispersed cytoplasmic. Clearly, such a diffuse population cannot be analyzed for interaction using co-localization. We now replaced Figure 1K, with new images that were acquired using fluorescent life-time imaging microscopy (see comment to point 3) where we also depict a zoom-in of the imaged granules.

3. Co-expression of BmAgo3 and BmVreteno make more granules structure, but the other combinations did not. Also, it is hard to say that BmGtsf1L makes granules dominantly with BmAgo3 and BmVreteno due to the frequency of granule and their dispersed cytoplasmic distribution. To make this point clear, the authors should examine all three proteins together for their co-localization.
To meet the reviewers' concern, we now analyzed the co-localization of BmGtsf1L, BmVreteno and BmAgo3 using fluorescent lifetime imaging microscopy (FLIM). With this technique we were now able to show that all three components co-localize in granules, further supporting our initial results. We exchanged this new data with the former data from Figure 1K.

4. Although the authors claim the interaction between BmGtsf1 and BmAgo3 under BmVreteno-KD, the signal with anti-GFP for Gtsf1 in Ago3 IP (Fig2E) is quite faint.
This is correct. However, our controls demonstrate the signal is not simply background, and hence is specific (also see Figure 1E). Probably only a small

fraction of BmGtsf1L is present in actively processing BmAgo3 bodies and most of the BmGtsf1L resides in the nucleocytoplasmic population (see reviewers' comment on point 2). A similar observation has been made for Drosophila Gtsf, were multiple Piwi IPs were pooled for the detection endogenous DmGtsf1 (Dönertas et al., Genes Dev 2013).

5. As mentioned in the general comments, the biological importance of these protein interaction needs to be shown. The authors could examine the activity for these complex and relevancy of the interaction for piRNA processing, i.e. the activity of the proper or malformed piRiSC, and TE repression. Is proper interaction between Gtsf1L and Vreteno required for Ago3 activity but not Siwi's? How their complex formation and piRNA loading are affected? Is it also possible to examine this point by checking their localization as well? \
The reviewer raises a good point, but also one that is very hard to address. The only way to really address this point would require the use recombinant BmAgo3 programmed with a specific piRNA that can recognize and subsequently cleave target RNA. Then one could probe the effect of BmGtsf1L and BmVreteno and be sure that one would detect also relatively small kinetic effects. Unfortunately, the field has thus far not been able to purify piRNA-loaded BmAgo3 to perform kinetic measurements like the experiments performed in Arif et al. (Nature 2022).
Nevertheless, we tried to set up an in vitro cleavage assay using immunopurified BmAgo3 from BmN4 cell lysates, similar to the experiment described by Izumi et al (RNA 2022). They showed that target RNA which was incubated with BmAgo3 immunoprecipitate alone was already sufficient for target RNA cleavage, which was modestly increased by BmGtsf1L, even though BmGtsf1L was added in high molar excess. Hence, we wanted to test whether the addition of recombinant BmVreteno AF eTD1 could further stimulate the cleavage activity of BmAgo3 piRISC in this assay. Unfortunately, we failed to set up such a cleavage assay.

Concretely, we have conducted the following experiments (Figure R1).

a) We set up the same experiment as described in Izumi et al (RNA 2022) using our FLAG-BmAgo3 stable cell line and the very same BmAgo3 target RNA sequence. However, we did not detect any 135-nt sized cleavage product, also not when recombinant BmGtsf1L and BmVreteno AF-eTD1 were added.
b) We designed eight additional BmAgo3 target RNAs, based on piRNA abundance of our own BmN4 cells, and also tested these in the slicer assay. Here, one target RNA (# 5) was degraded after the addition of immunopurified BmAgo3 and all other targets again did not yield the 32-P labeled 5'-end cleavage product of ~ 135-nt.
c) We finally tested four different RNA targets and incubated these with anti-BmAgo3 immunoprecipitants from naïve BmN4 cells to exclude the possibility that the triple FLAG-tag from the FLAG-BmAgo3 stable cell line could interfere with slicer activity or that (poly)clonal expansion had affected the piRNA

repertoire. Unfortunately, we could again not detect any cleavage product of the expected size, with or without GTSF1L and Vreteno.

Figure R1

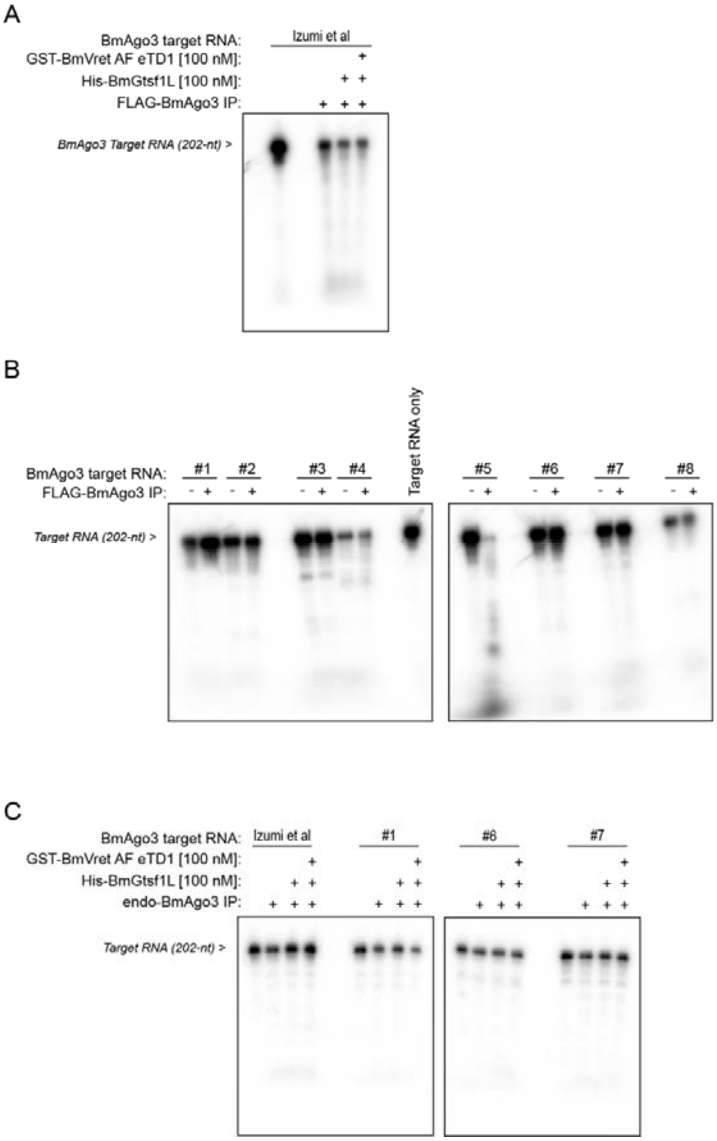

To conclude, we were unable to study how the BmGtsf1L-BmVreteno-BmAgo3 interaction affects piRISC activity because the in vitro cleavage assay was not functional in our hands. However, we note that Izumi et al (RNA 2022) already described (minor) effects of BmGtsf1L knockdown on total mature piRNA levels and a concomitant reduction in the strength of the ping-pong signature, fully in line with what we propose.

- Finally, the reviewer asks to use localization as a means to address this question. Nishida et al. (EMBO J 2020) already described that BmAgo3 bodies cannot be formed upon BmVreteno knockdown and that BmAgo3 remains dispersed cytosolic.

BmVreteno also lost its granularity upon BmAgo3 depletion, indicating that both components are required for BmAgo3 body formation. Unfortunately, the GTSF1L antibody did not work in immune stainings, prohibiting a meaningful analysis of BmGtsf1L localization in absence of BmVreteno. Note, however, that we did show that overexpressed BmGtsf1L mostly localizes to perinuclear granules when also BmVreteno is overexpressed (Figure 1K) indicating that the two depend on each other.

6. In Fig3DE: the morphology and size of the cells expressing BmGstf1 w99A differ from the wild type. The viability and healthiness of the cell line upon expressing Gstf1 w99A should be shown. It is needed for SupFigS4 in the series of the mutants of the hydrophobic pocket.
Naïve BmN4 cells show a wide variety of shapes and morphologies, and many vacuoles are present. It has also been reported that this cell line grows as a dual layer with cells of different morphology (Xiol et al., Cell 2014).
We acknowledge that meddling with the piRNA pathway could affect the cell morphology. Likewise, transfection as well as cell density can also affect piRNA biology (Honda et al., Sci Rep 2017). However, we do not think that these aspects are relevant for this study. The point that we want to make here is that the BmGtsf1L-W99A mutant fails to interact with BmVreteno and does not co-localize with BmVreteno-BmAgo3 marked granules.

Minor concerns
1. Fig1B experiment appearently uses s-BmVreteno, and RNase treatment did not affect the interaction with Gtsf1L. I wonder how is that with L-BmVreteno containing RRM domain.
The BmVreteno-L isoform has been used for this experiment. We now changed the labeling in the figure panel to clarify this better.
2. Change the capital for the figure legend to be consistent with Fig a→A.
We adjusted this throughout the manuscript.
3. In Fig5A heatmap (bottom), it should be BmGtsf1, not BmGtsf2.
We changed the labeling of this figure panel.
4. Use the serial numbers for the lines: each page starts with line1 and it's confusing.
We feel that this is a matter of personal style/preference and prefer to keep this like it is.

1st Revision - Editorial Decision **11th Oct 2023**

Dear Walter,

Thank you for submitting a revised version of your manuscript. Your study has now been seen by one of the original referees, who finds that most of their previous concerns have been addressed and is now broadly in favour of the acceptance of the manuscript.

There now remain only a few, mainly editorial, points that have to be addressed before I can extend acceptance of the manuscript:

1. Please address the remaining minor points of referee #2.
2. Please check that the funding information is correct and identical both in the manuscript and our online system. The following grant information is currently missing from our online system: Deutsche Forschungsgemeinschaft (DFG, German Research Foundation) project number 497669232; IMB Proteomics Core Facility is gratefully acknowledged (instrument funded by DFG INST 247/766-1 FUGG); IMB Genomics Core Facility and the use of its NextSeq500 (funded by the DFG - INST 247/870-1 FUGG); Ministry of Science and Health (MWG), Rhineland Palatinate (funding id: TB-Nr.:3658/19).
3. Please check the email provided for Lukas Stelzl (l.stelzl@imb-main.de) - emails sent to this address do not reach the addressee.
4. CRediT has replaced the traditional author contributions section because it offers a systematic, machine-readable author contributions format that allows for more effective research assessment. Please remove the Authors Contributions from the manuscript and use the free text boxes beneath each contributing author's name in our online submission system to add specific details on the author's contribution. More information is available in our guide to authors.
5. Please rename "Conflict of interest" section into "Disclosure and competing interests statement" (further info: https://www.embopress.org/page/journal/14602075/authorguide#conflictsofinterest).
6. Table S1 and S3 should be renamed to Dataset EV2-EV3 with the legend included as a separate sheet in each Excel file and corresponding callouts. Table S2 should be renamed to Table EV1 with the appropriate callout.
7. Please rename the movie into Movie EV1 and update the callouts accordingly. The legend should be zipped with the movie file. Further information is available here: https://www.embopress.org/page/journal/14602075/authorguide#expandedview
8. Our data editors have flagged the following issues in figure legends that need correcting:
- In the legend of figure 1h, the box plots need to be defined in terms of minima, maxima, centre, bounds of box and whiskers, and percentile.
- Please define the number and the nature of the replicates (e.g., biological or technical) in the legends of figure 1h.
- The measure of centre for the error bars needs to be defined in the legend of figure 4g.
9. Please assemble source data into one folder per figure and upload as .zip files. For example, the Source data files for figure 1 need to be saved in a single folder and this needs to be zipped and then uploaded as "SD figure 1.zip" file.
10. In source data for Figure 1B, HA-IP anti-Flag, please check that the 32 kDA bands are correctly marked, as the image is different between the figure and source data.
11. The source data for anti-actin staining in figure 2C does not appear to fit the main panel.

Please let me know if you have any questions regarding any of these points. You can use the link below to upload the revised files.

Thank you again for giving us the chance to consider your manuscript for The EMBO Journal. I look forward to receiving the final version.

With best wishes,

Ieva

Ieva Gailite, PhD
Senior Scientific Editor
The EMBO Journal
Meyerhofstrasse 1
D-69117 Heidelberg
Tel: +4962218891309
i.gailite@embojournal.org

We realize that it is difficult to revise to a specific deadline. In the interest of protecting the conceptual advance provided by the work, we recommend a revision within 3 months (9th Jan 2024). Please discuss the revision progress ahead of this time with the editor if you require more time to complete the revisions.

Referee #2:

The authors provided satisfactory responses to most of the issues raised by the reviewer. However, there are some remaining issues.

1. As to new data of co-localization taken by FLIM, the reviewer appreciates the authors' efforts for obtaining it. However, it needs some quantification. For instance, what the % of Gtsf1 overlaps with BmAgo3-BmVreteno granules?
2. Fig3DE: the point that we want to make here is that the BmGtsf1L- W99A mutant fails to interact with BmVreteno and does not co-localize with BmVreteno-BmAgo3 marked granules.
OK, but magnified image of the granules should be shown.
3. It is very unfortunate that the cleavage assay failed, though the other group successfully conducted. It was the most critical issue among those pointed by the reviewer. The authors could argue the issue in Discussion, highlighting its importance to be examined in future study.

Referee #2:

The authors provided satisfactory responses to most of the issues raised by the reviewer. However, there are some remaining issues.

1. As to new data of co-localization taken by FLIM, the reviewer appreciates the authors' efforts for obtaining it. However, it needs some quantification. For instance, what the % of Gtsf1 overlaps with BmAgo3-BmVreteno granules?

We have quantified these numbers using the mean intensity values of both the granule and the overall cell region and could estimate that a factor 5.46 and 9.5 more signal is present in the granules for replicate #1 and #2, respectively (Fig 1K). At the same time, this also reveals that there is variation between individual cells that we attributed to our overexpression system.

We therefore decided to show a line plot, representing the intensity gray values for each fluorophore, showing that the vast majority of BmGtsf1L is present within the granule and a small fraction of BmGtsf1L is detected outside the granule (Fig 1K, right panel). Furthermore, we now also represent these line plots in Figure EV2A and uploaded the quantification of the data as Dataset EV4.

2. Fig3DE: the point that we want to make here is that the BmGtsf1L- W99A mutant fails to interact with BmVreteno and does not co-localize with BmVreteno-BmAgo3 marked granules.

OK, but magnified image of the granules should be shown.

We now show a magnified image of the boxed-area (inset) for Fig 3D and 3E..

3. It is very unfortunate that the cleavage assay failed, though the other group successfully conducted. It was the most critical issue among those pointed by the reviewer. The authors could argue the issue in Discussion, highlighting its importance to be examined in future study.

We agree with the reviewer and we are disappointed that we did not manage to establish a functional cleavage assay. We have now added a small section to the discussion, describing that piRISC kinetic studies would need to be addressed in vitro.

2nd Revision - Editorial Decision 30th Oct 2023

Dear Walter,

Thank you for addressing most of the final editorial issues. I am now pleased to inform you that your manuscript has been accepted for publication.

Before we forward your manuscript to our publishers, there remain a couple of points that need addressing:
1. I am afraid that the source data panel for Figure 2C, anti-actin still does not fit to the figure. You can send me the correct file via email.
2. I would like to propose a couple of minor changes in the article abstract and synopsis. I have also written a short blurb that will accompany the title of your manuscript in our online table of contents. Please take a look at the text below and in the attached manuscript text file and let me know if any corrections are necessary.

Blurb:
BmVreteno serves as a molecular scaffold to interconnect BmGtsf1L and piRISC-BmAgo3 complex for efficient piRNA amplification.

Synopsis:
In the silkworm Bombyx mori, piRNA amplification requires BmVreteno, which enhances re-loading of the empty Siwi protein after piRNA-target cleavage. Here, a single eTudor domain within BmVreteno is shown to interconnect piRISC-BmAgo3 with BmGtsf1L, a protein known to enhance BmAgo3 activity, to ensure efficient piRNA amplification.

• BmGtsf1L associates with piRNA-loaded BmAgo3 and resides in BmAgo3 bodies.
• BmVreteno stimulates the interaction between BmGtsf1L and BmAgo3-containing piRISC.
• The C-terminal tail of BmGtsf1L directly interacts with the eTD1 domain of BmVreteno via a newly identified hydrophobic pocket.
• BmGtsf1L and BmAgo3 bind to the same eTudor domain of BmVreteno.

Please note that it is EMBO Journal policy for the transcript of the editorial process (containing referee reports and your response letter) to be published as an online supplement to each paper. If you do NOT want this, you will need to inform the Editorial Office via email immediately. More information is available here: https://www.embopress.org/page/journal/14602075/authorguide#transparentprocess

Your manuscript will be processed for publication in the journal by EMBO Press. Manuscripts in the PDF and electronic editions of The EMBO Journal will be copy edited, and you will be provided with page proofs prior to publication. Please note that supplementary information is not included in the proofs.

You will be contacted by Wiley Author Services to complete licensing and payment information. The required 'Page Charges Authorization Form' is available here: https://www.embopress.org/pb-assets/embo-site/tej_apc.pdf - please download and complete the form and return to embopressproduction@wiley.com

EMBO Press participates in many Publish and Read agreements that allow authors to publish Open Access with reduced/no publication charges. Check your eligibility: https://authorservices.wiley.com/author-resources/Journal-Authors/open-access/affiliation-policies-payments/index.html

Should you be planning a Press Release on your article, please get in contact with embojournal@wiley.com as early as possible, in order to coordinate publication and release dates.

If you have any questions, please do not hesitate to call or email the Editorial Office. Thank you again for this contribution to The EMBO Journal and congratulations on a successful publication!

Best regards,

Ieva

Ieva Gailite, PhD
Senior Scientific Editor
The EMBO Journal
Meyerhofstrasse 1
D-69117 Heidelberg
Tel: +4962218891309

i.gailite@embojournal.org

EMBO Press Author Checklist

Corresponding Author Name: Alfred Willem Bronkhorst	
Journal Submitted to: EMBO J	
Manuscript Number: 114072	

USEFUL LINKS FOR COMPLETING THIS FORM
The EMBO Journal - Author Guidelines
EMBO Reports - Author Guidelines
Molecular Systems Biology - Author Guidelines
EMBO Molecular Medicine - Author Guidelines

Reporting Checklist for Life Science Articles (updated January
This checklist is adapted from Materials Design Analysis Reporting (MDAR) Checklist for Authors. MDAR establishes a minimum set of requirements in transparent reporting in the life sciences (see Statement of Task: 10.31222/osf.io/9sm4x). Please follow the journal's guidelines in preparing your
Please note that a copy of this checklist will be published alongside your article.

Abridged guidelines for figures
1. Data
The data shown in figures should satisfy the following conditions:
- the data were obtained and processed according to the field's best practice and are presented to reflect the results of the experiments in an accurate and unbiased manner.
- ideally, figure panels should include only measurements that are directly comparable to each other and obtained with the same assay.
- plots include clearly labeled error bars for independent experiments and sample sizes. Unless justified, error bars should not be shown for technical
- if n<5, the individual data points from each experiment should be plotted. Any statistical test employed should be justified.
- Source Data should be included to report the data underlying figures according to the guidelines set out in the authorship guidelines on Data

2. Captions
Each figure caption should contain the following information, for each panel where they are relevant:
- a specification of the experimental system investigated (eg cell line, species name).
- the assay(s) and method(s) used to carry out the reported observations and measurements.
- an explicit mention of the biological and chemical entity(ies) that are being measured.
- an explicit mention of the biological and chemical entity(ies) that are altered/varied/perturbed in a controlled manner.
- the exact sample size (n) for each experimental group/condition, given as a number, not a range;
- a description of the sample collection allowing the reader to understand whether the samples represent technical or biological replicates (including how many animals, litters, cultures, etc.).
- a statement of how many times the experiment shown was independently replicated in the laboratory.
- definitions of statistical methods and measures:
  - common tests, such as t-test (please specify whether paired vs. unpaired), simple χ2 tests, Wilcoxon and Mann-Whitney tests, can be unambiguously identified by name only, but more complex techniques should be described in the methods section;
  - are tests one-sided or two-sided?
  - are there adjustments for multiple comparisons?
  - exact statistical test results, e.g., P values = x but not P values < x;
  - definition of 'center values' as median or average;
  - definition of error bars as s.d. or s.e.m.

Please complete ALL of the questions below.
Select "Not Applicable" only when the requested information is not relevant for your study.

Materials

Newly Created Materials	Information included in the manuscript?	In which section is the information available? (Reagents and Tools Table, Materials and Methods, Figures, Data Availability Section)
New materials and reagents need to be available; do any restrictions apply?	Yes	Materials and Methods section

Antibodies	Information included in the manuscript?	In which section is the information available? (Reagents and Tools Table, Materials and Methods, Figures, Data Availability Section)
For **antibodies** provide the following information:<br>- Commercial antibodies: RRID (if possible) or supplier name, catalogue number and or/clone number<br>- Non-commercial: RRID or citation	Yes	Materials and Methods (Antibodies)

DNA and RNA sequences	Information included in the manuscript?	In which section is the information available? (Reagents and Tools Table, Materials and Methods, Figures, Data Availability Section)
**Short novel DNA or RNA including primers, probes**: provide the sequences.	Yes	Table S3

Cell materials	Information included in the manuscript?	In which section is the information available? (Reagents and Tools Table, Materials and Methods, Figures, Data Availability Section)
**Cell lines:** Provide species information, strain. Provide accession number in repository **OR** supplier name, catalog number, clone number, and/**OR** RRID.	Yes	Materials and Methods (BmN4 cell culture and transfection)
**Primary cultures:** Provide species, strain, sex of origin, genetic modification status.	Not Applicable	
Report if the cell lines were recently **authenticated** (e.g., by STR profiling) and tested for mycoplasma contamination.	Yes	Materials and Methods (BmN4 cell culture and transfection)

Experimental animals	Information included in the manuscript?	In which section is the information available? (Reagents and Tools Table, Materials and Methods, Figures, Data Availability Section)
**Laboratory animals or Model organisms:** Provide species, strain, sex, age, genetic modification status. Provide accession number in repository **OR** supplier name, catalog number, clone number, **OR** RRID.	Not Applicable	
**Animal observed in or captured from the field:** Provide species, sex, and age where possible**.**	Not Applicable	
Please detail **housing and husbandry conditions**.	Not Applicable	

Plants and microbes	Information included in the manuscript?	In which section is the information available? (Reagents and Tools Table, Materials and Methods, Figures, Data Availability Section)
**Plants:** provide species and strain, ecotype and cultivar where relevant, unique accession number if available, and source (including location for collected wild specimens).	Not Applicable	
**Microbes:** provide species and strain, unique accession number if available, and source.	Not Applicable	

Human research participants	Information included in the manuscript?	In which section is the information available? (Reagents and Tools Table, Materials and Methods, Figures, Data Availability Section)
If collected and within the bounds of privacy constraints report on age, sex and gender or ethnicity for all study participants.	Not Applicable	

Core facilities	Information included in the manuscript?	In which section is the information available? (Reagents and Tools Table, Materials and Methods, Figures, Data Availability Section)
If your work benefited from core facilities, was their service mentioned in the acknowledgments section?	Yes	Acknowledgements & co-authorship

Design

Study protocol	Information included in the manuscript?	In which section is the information available? (Reagents and Tools Table, Materials and Methods, Figures, Data Availability Section)
If study protocol has been **pre-registered, provide DOI in the manuscript**. For clinical trials, provide the trial registration number **OR** cite DOI.	Not Applicable	
Report the **clinical trial registration number** (at ClinicalTrials.gov or equivalent), where applicable.	Not Applicable	

Laboratory protocol	Information included in the manuscript?	In which section is the information available? (Reagents and Tools Table, Materials and Methods, Figures, Data Availability Section)
Provide DOI OR other citation details if **external detailed step-by-step protocols** are available.	Not Applicable	

Experimental study design and statistics	Information included in the manuscript?	In which section is the information available? (Reagents and Tools Table, Materials and Methods, Figures, Data Availability Section)
Include a statement about **sample size** estimate even if no statistical methods were used.	Not Applicable	
Were any steps taken to minimize the effects of subjective bias when allocating animals/samples to treatment (e.g. **randomization procedure**)? If yes, have they been described?	Not Applicable	
Include a statement about **blinding** even if no blinding was done.	Not Applicable	
Describe **inclusion/exclusion criteria** if samples or animals were excluded from the analysis. Were the criteria pre-established?<br><br>If sample or data points were omitted from analysis, report if this was due to attrition or intentional exclusion and provide justification.	Not Applicable	
For every figure, are **statistical tests** justified as appropriate? Do the data meet the assumptions of the tests (e.g., normal distribution)? Describe any methods used to assess it. Is there an estimate of variation within each group of data? Is the variance similar between the groups that are being statistically compared?	Not Applicable	

Sample definition and in-laboratory replication	Information included in the manuscript?	In which section is the information available? (Reagents and Tools Table, Materials and Methods, Figures, Data Availability Section)
In the figure legends: state number of times the experiment was **replicated** in laboratory.	Yes	Figure legend
In the figure legends: define whether data describe **technical or biological replicates**.	Yes	Figure legend

Ethics

Ethics	Information included in the manuscript?	In which section is the information available? (Reagents and Tools Table, Materials and Methods, Figures, Data Availability Section)
Studies involving **human participants**: State details of **authority granting ethics approval** (IRB or equivalent committee(s), provide reference number for approval.	Not Applicable	
Studies involving **human participants**: Include a statement confirming that **informed consent** was obtained from all subjects and that the experiments conformed to the principles set out in the WMA Declaration of Helsinki and the Department of Health and Human Services Belmont Report.	Not Applicable	
Studies involving **human participants: For** publication of **patient photos**, include a statement confirming that consent to publish was obtained.	Not Applicable	
Studies involving experimental **animals**: State details of **authority granting ethics approval** (IRB or equivalent committee(s), provide reference number for approval. Include a statement of compliance with ethical regulations.	Not Applicable	
Studies involving **specimen and field samples:** State if relevant **permits** obtained, provide details of authority approving study; if none were required, explain why.	Not Applicable	

Dual Use Research of Concern (DURC)	Information included in the manuscript?	In which section is the information available? (Reagents and Tools Table, Materials and Methods, Figures, Data Availability Section)
Could your study fall under dual use research restrictions? Please check biosecurity documents and list of **select agents and toxins** (CDC): https://www.selectagents.gov/sat/list.htm	Not Applicable	
If you used a select agent, is the security level of the lab appropriate and reported in the manuscript?	Not Applicable	
If a study is subject to dual use research of concern regulations, is the name of the **authority granting approval and reference number** for the regulatory approval provided in the manuscript?	Not Applicable	

Reporting

The MDAR framework recommends adoption of discipline-specific guidelines, established and endorsed through community initiatives. Journals have their own policy about requiring specific guidelines and recommendations to complement MDAR.

Adherence to community standards	Information included in the manuscript?	In which section is the information available? (Reagents and Tools Table, Materials and Methods, Figures, Data Availability Section)
State if relevant guidelines or checklists (e.g., **ICMJE, MIBBI, ARRIVE, PRISMA**) have been followed or provided.	Not Applicable	
For **tumor marker prognostic studies,** we recommend that you follow the **REMARK** reporting guidelines (see link list at top right). See author guidelines, under 'Reporting Guidelines'. Please confirm you have followed these guidelines.	Not Applicable	
For **phase II and III randomized controlled trials**, please refer to the **CONSORT** flow diagram (see link list at top right) and submit the CONSORT checklist (see link list at top right) with your submission. See author guidelines, under 'Reporting Guidelines'. Please confirm you have submitted this list.	Not Applicable	

Data Availability

Data availability	Information included in the manuscript?	In which section is the information available? (Reagents and Tools Table, Materials and Methods, Figures, Data Availability Section)
Have **primary datasets** been deposited according to the journal's guidelines (see 'Data Deposition' section) and the respective accession numbers provided in the Data Availability Section?	Yes	Data and material availability
Were **human clinical and genomic datasets** deposited in a public access-controlled repository in accordance to ethical obligations to the patients and to the applicable consent agreement?	Not Applicable	
Are **computational models** that are central and integral to a study available without restrictions in a machine-readable form? Were the relevant accession numbers or links provided?	Yes	MD simulation data related to Fig 5C, 5D, 5E and EV4B, EV4C. 10.5281/zenodo.8388721
If publicly available data were reused, provide the respective **data citations in the reference list**.	Not Applicable	
